# Supplementary material for: The hepatic compensatory response to elevated systemic sulfide promotes diabetes
Source: Cell Rep. 2021 Nov 9;37(6):109958. doi: 10.1016/j.celrep.2021.109958 (PMC8595646; doi:10.1016/j.celrep.2021.109958)
Supplement: Document S1. Figures S1–S7 and Tables S1, S2, and S4–S9 [file mmc1.pdf]

Cell Reports, Volume *37*

Supplemental information

The hepatic compensatory response

to elevated systemic sulfide promotes diabetes

Roderick N. Carter, Matthew T.G. Gibbins, Martin E. Barrios-Llerena, Stephen E. Wilkie, Peter L. Freddolino, Marouane Libiad, Victor Vitvitsky, Barry Emerson, Thierry Le Bihan, Madara Brice, Huizhong Su, Scott G. Denham, Natalie Z.M. Homer, Clare Mc Fadden, Anne Tailleux, Nourdine Faresse, Thierry Sulpice, Francois Briand, Tom Gillingwater, Kyo Han Ahn, Subhankar Singha, Claire McMaster, Richard C. Hartley, Bart Staels, Gillian A. Gray, Andrew J. Finch, Colin Selman, Ruma Banerjee, and Nicholas M. Morton

Figure S1. *Tst* mRNA tissue expression profile in C57BL/6J mice and the metabolic fates of $^{13}C_3$ pyruvate in hepatocytes from *Tst*$^{-/-}$ mice. Related to Figure 1

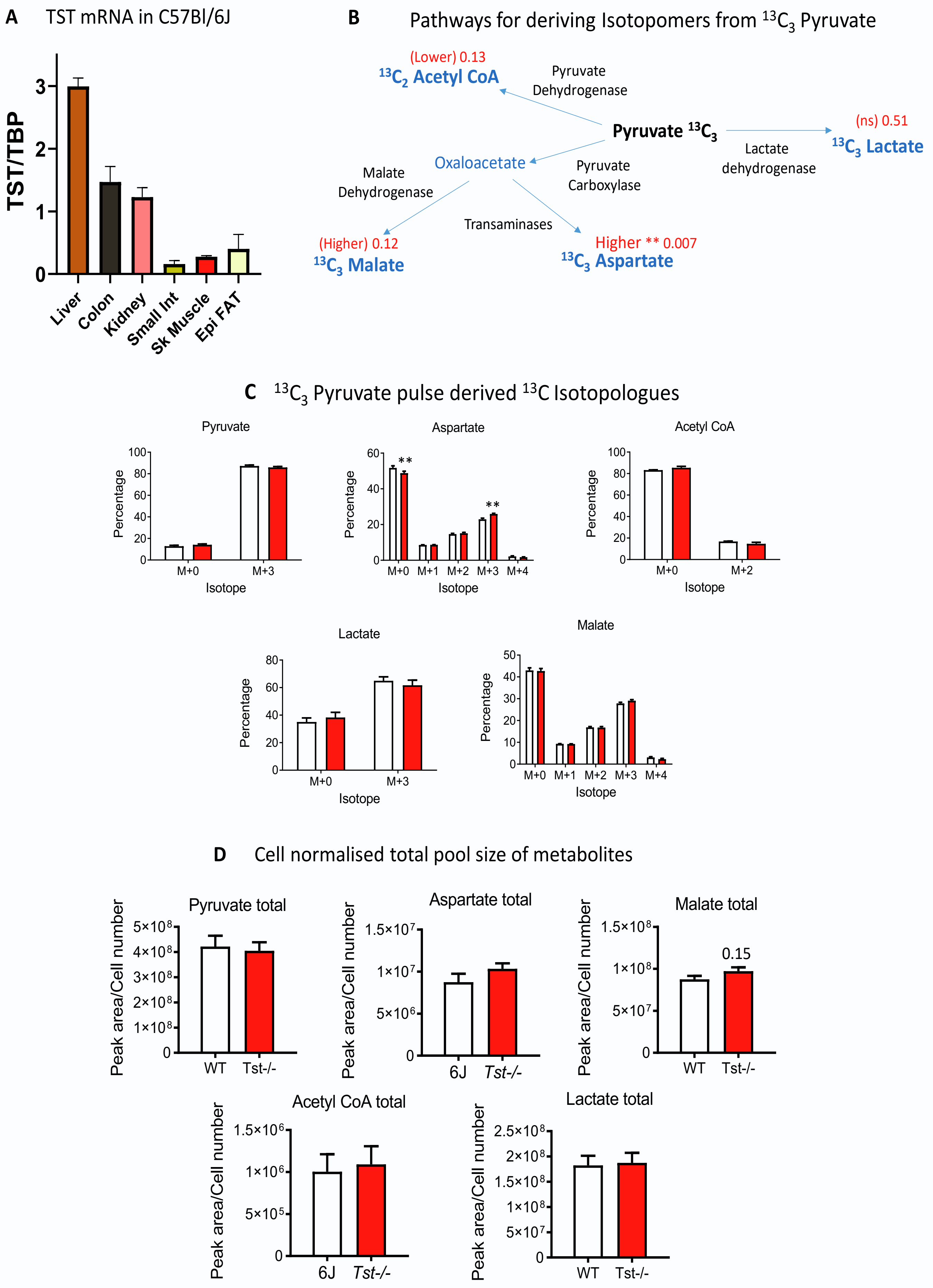

Figure S1. *Tst* mRNA tissue expression profile in C57BL/6J mice and the metabolic fates of $^{13}C_3$ pyruvate in hepatocytes from *Tst*$^{-/-}$ mice. Related to Figure 1 **(A)** Histogram showing *Tst* mRNA level across liver, colon, kidney, small intestine, skeletal muscle and epididymal fat from male C57BL/6J mice measured by realtime-PCR and normalised to *Tbp* mRNA **(B)** Diagram representing metabolites derived from pyruvate. Oxaloacetate was not detected but is indicated as an intermediate to production of malate or aspartate. The isotopologue shown on the diagram represents to most abundant detected following pulse with $^{13}C_3$ pyruvate. In red is the direction of change in hepatocytes of *Tst*$^{-/-}$ mice, with significance or P-values (when less than 0.2) from t-tests**.** (**C**) Histogram showing isotopologues derived from $^{13}C_3$ pyruvate from C57Bl/6J (white bars, n = 5) and *Tst*$^{-/-}$ (red bars n = 4) cultured hepatocytes. Data represents the amount of isotopologues detected by mass spectrometry, as a percentage of the total detected metabolite (total includes unlabelled ^{12}C and all detected ^{13}C isotopologues). Counts were first normalised to cell number. **(D)** Histograms showing the total pool size of each metabolite. Data is cell normalised mass spec counts from all isotopologues of the given metabolite, including the relevant unlabelled ^{12}C species. Data are represented as mean ± SEM. Each metabolite was analysed using a t-test, ** indicates that $P < 0.01$. P-values less than 0.2 are also indicated for showing potential trends.

Figure S2. Insulin–regulated metabolic parameters in liver and plasma of *Tst*$^{-/-}$ mice. Related to Figure 1.

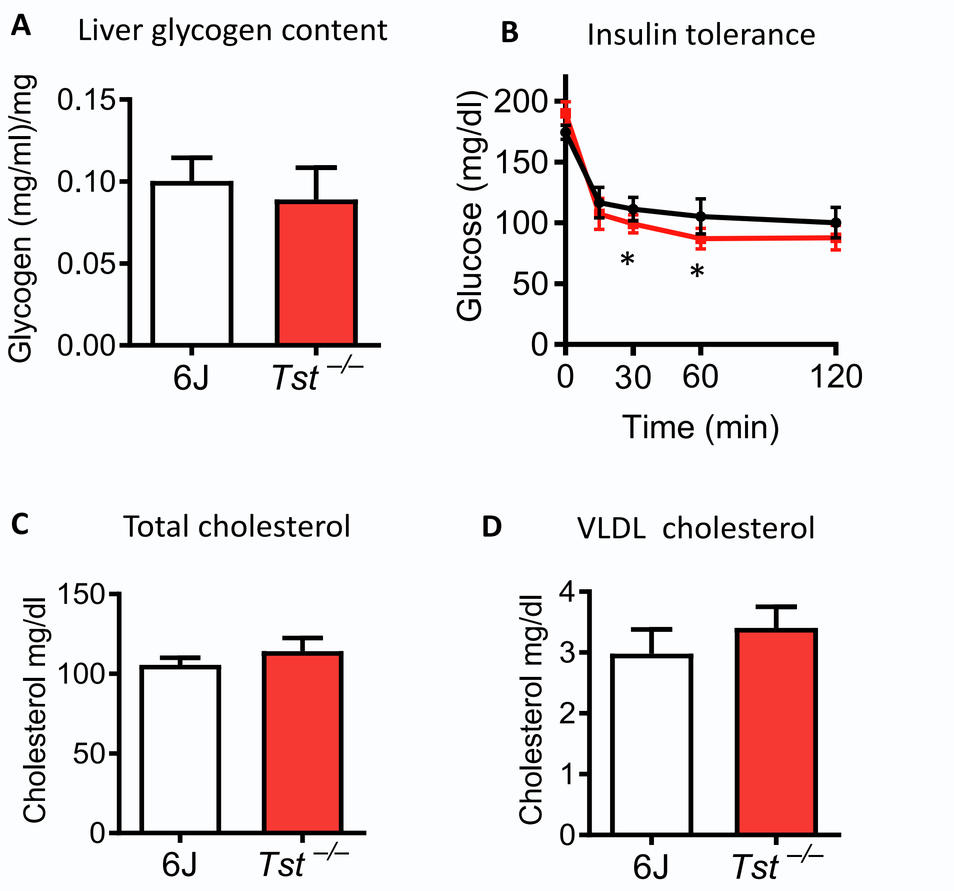

Figure S2. Insulin–regulated metabolic parameters in liver and plasma of *Tst*$^{-/-}$ mice. Related to Figure 1. (**A**) Glycogen measured from whole liver from normal diet-fed 4 hour fasted C57Bl/6J (6J; white bar, n = 5), and *Tst*$^{-/-}$ (red bar, n = 5) mice. Data are represented as mean ±SEM. (**B**) Plasma glucose (mg/dl), over 120 minutes following insulin administration (i.p., 1mU/g) in normal diet-fed 4 hour fasted C57Bl/6J (black line, n = 8) and *Tst*$^{-/-}$ (red line, n = 7) mice. (**C**) HPLC quantified total plasma cholesterol in normal diet-fed 4 hour fasted C57Bl/6J (white bar, n = 6) and *Tst*$^{-/-}$ (red bar, n = 6) mice. (**D**) HPLC quantified VLDL plasma cholesterol in normal diet-fed 4 hour fasted C57Bl/6J (white bar, n = 6), and *Tst*$^{-/-}$ (red bar, n = 6) mice. For (**B**) a Repeated Measures analysis demonstrated a significant effect of time (****) and an interaction between time and genotype (*). T-tests revealed that the decrement of glucose from baseline at 30 and 60 minutes after insulin was greater in the *Tst*$^{-/-}$ (*).

Figure S3. Hepatocytes from *Tst*$^{-/-}$ mice resist hydrogen peroxide induced mitochondrial reactive species accumulation. Related to Figure 2 and Table 1

A

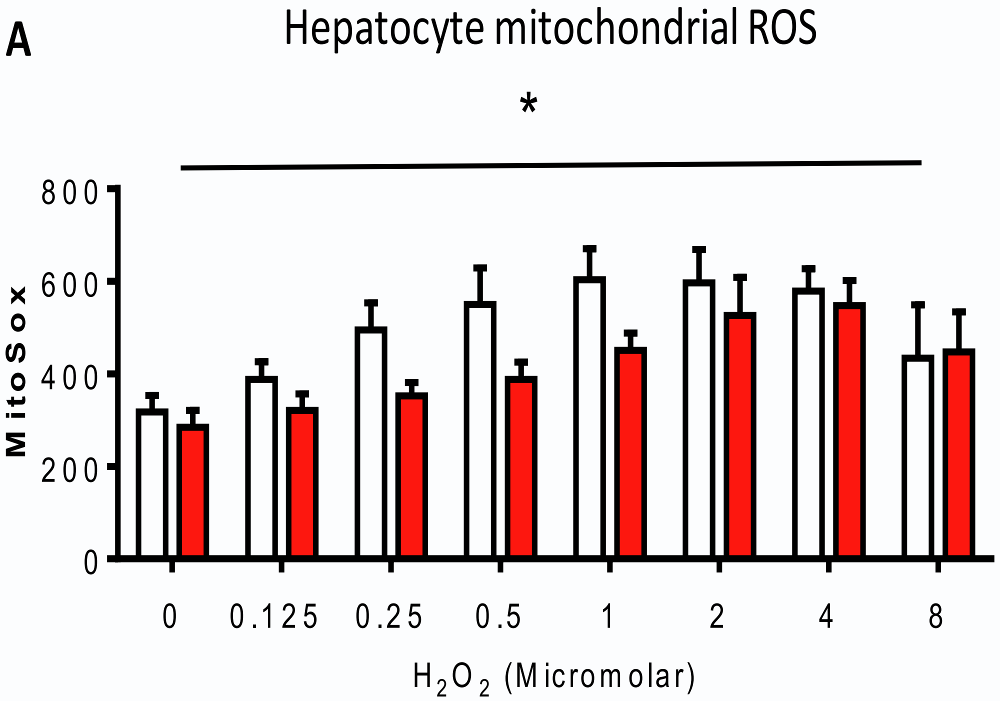

Figure S3. Hepatocytes from *Tst*$^{-/-}$ mice resist hydrogen peroxide induced mitochondrial reactive species accumulation. Related to Figure 2 and Table 1 **(A)** Mitochondrial reactive oxygen species measured from primary hepatocytes by MitoSox fluorescence from C57Bl/6J (white bars, n = 7) and *Tst*$^{-/-}$ (red bars, n = 7). Cells were exposed to a range of doses of H_2O_2 prior to MitoSox incubation and fluorescent detection. Data are represented as mean ±SEM. Significance was calculated using 2-WAY ANOVA for H_2O_2 dose and genotype. A significant effect of genotype is represented above the histogram with a *. H_2O_2 was significant to $P < 0.001$ (not represented on the histogram).

Figure S4. Persulfidation in the gluconeogenesis pathway is significantly different to global persulfidation patterns in the liver of the *Tst*$^{-/-}$ mice. Related to Figure 2 and Table 3.

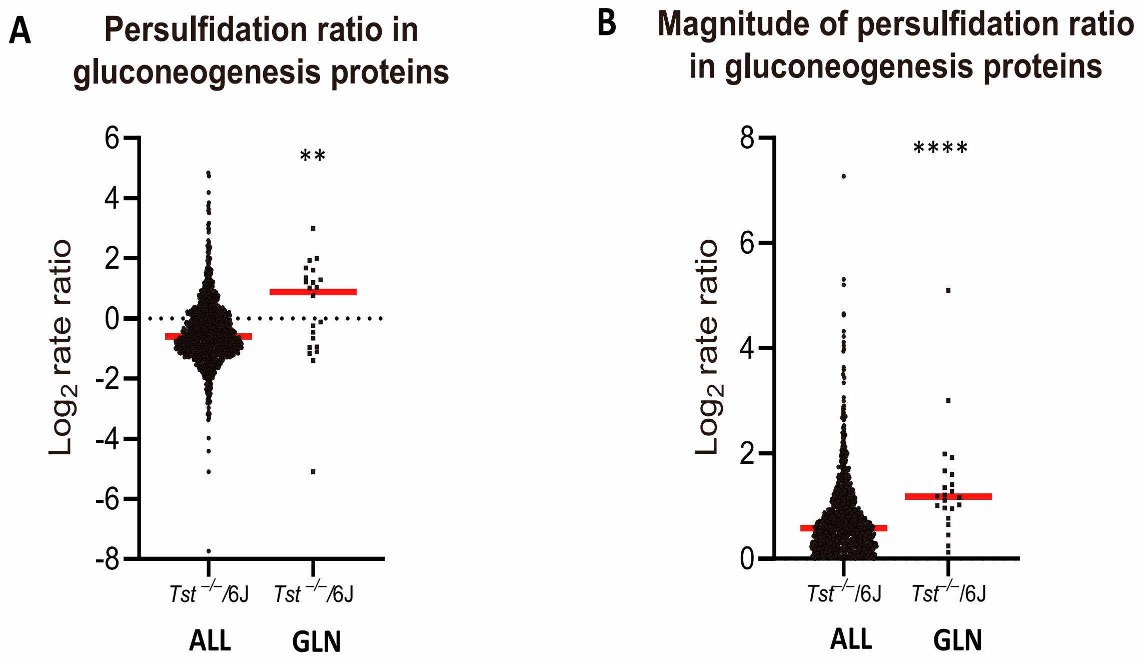

Figure S4. Persulfidation in the gluconeogenesis pathway is significantly different to global persulfidation patterns in the liver of the *Tst*$^{-/-}$ mice. Related to Figure 1, Figure 2 and Table 3. (**A**) Beeswarm plots showing the persulfidation log_2 rate ratio (*Tst*$^{-/-}$ divided by 6J) for peptides in the entire data set (ALL), alongside the log_2 rate ratio for peptides corresponding to proteins of gluconeogenesis (GLN). (**B**) Beeswarm plots showing the magnitude of the log_2 rate ratio (independent to direction of change), for peptides in the entire data set (ALL), alongside the log_2 rate ratios for peptides corresponding to proteins of gluconeogenesis (GLN). Data are represented as individual peptide log_2 rate ratio values, with the median represented as a red line. Significance was calculated using the Mann-Whitney U non parametric T-test. ** $P < 0.01$, **** $P < 0.0001$.

Figure S5. Validation of proteomic profiles by select western blot is exemplified by increased mitochondrial MPST. Related to Figure 3 and Table 3.

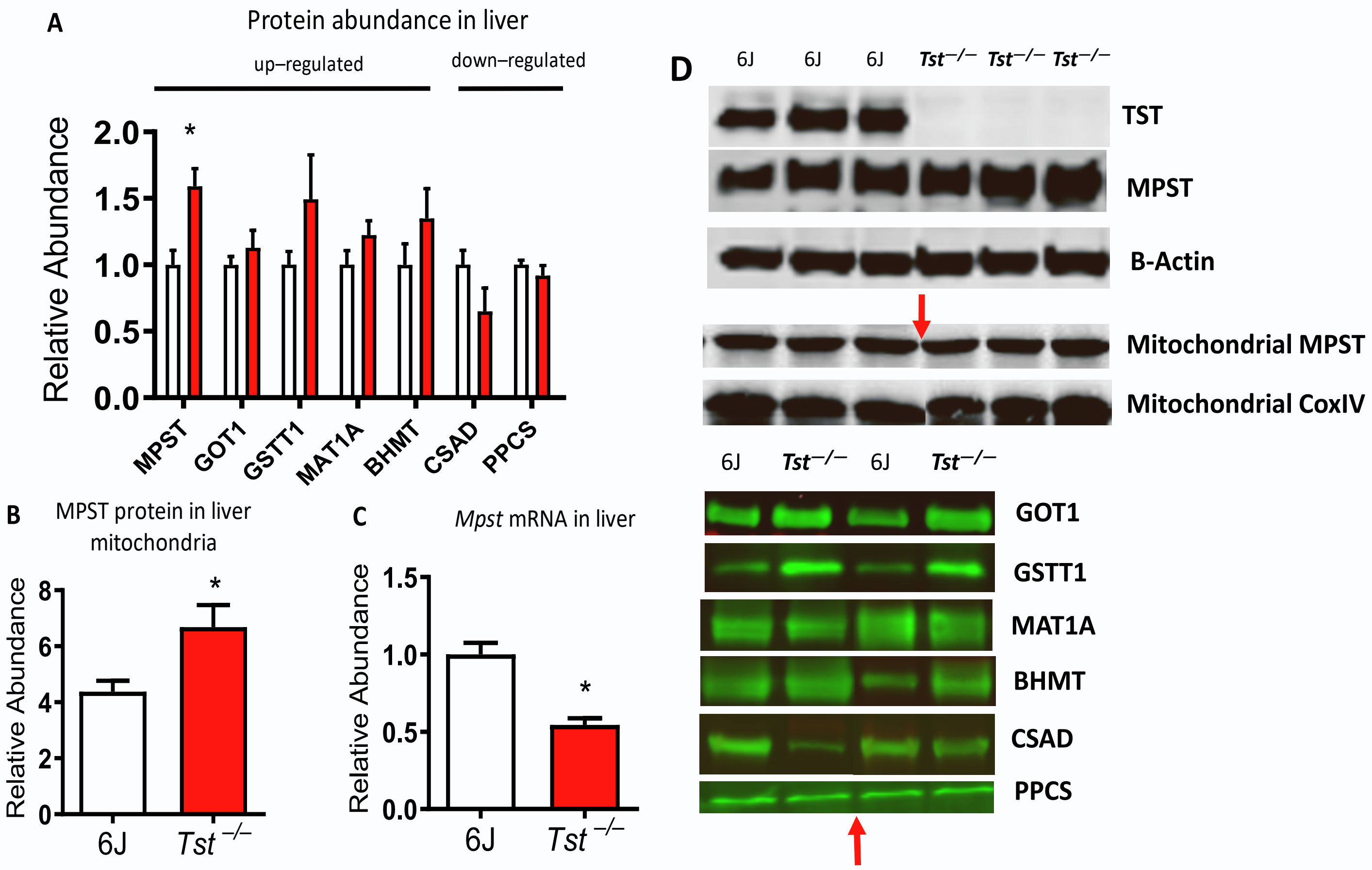

Figure S5. Validation of proteomic profiles by select western blot is exemplified by increased mitochondrial MPST. Related to Figure 2 and Table 3. (**A**) Quantification of western blots for a range of proteins found significantly up or down-regulated in the liver proteome of normal diet-fed 4 hour fasted C57Bl/6J (6J; white bar, n = 4-6) and *Tst*$^{-/-}$ (red bar, n = 4-6) mice. (**B**) Quantification of western blots for MPST from isolated liver mitochondria of normal diet-fed 4 hour fasted C57Bl/6J (white bar, n = 6), and *Tst*$^{-/-}$ (red bar, n = 6) mice. (**C**) *Mpst* mRNA quantified by real time PCR from liver of normal diet-fed C57Bl/6J (6J; white bar, n = 6) and *Tst*$^{-/-}$ (red bar, n = 6) mice. (**D**) Representative blots from LICOR imaging for the data quantified in (**A**). Red arrow indicates where superfluous lanes have been removed to simplify visualisation of genotype comparisons (GOT1, MAT1A, BHMT CSAD, mitochondrial MPST and COXIV). Data are represented as mean ±SEM. Significance was calculated using un-paired two-tailed student's t-test. * $P < 0.05$, ** $P < 0.01$, *** $P < 0.001$.

Figure S6. Hepatic proteins enriched in *Tst* $^{-/-}$ mice show under-representation of NRF2 promoter binding sites. Related to Figure 3 and Table 3.

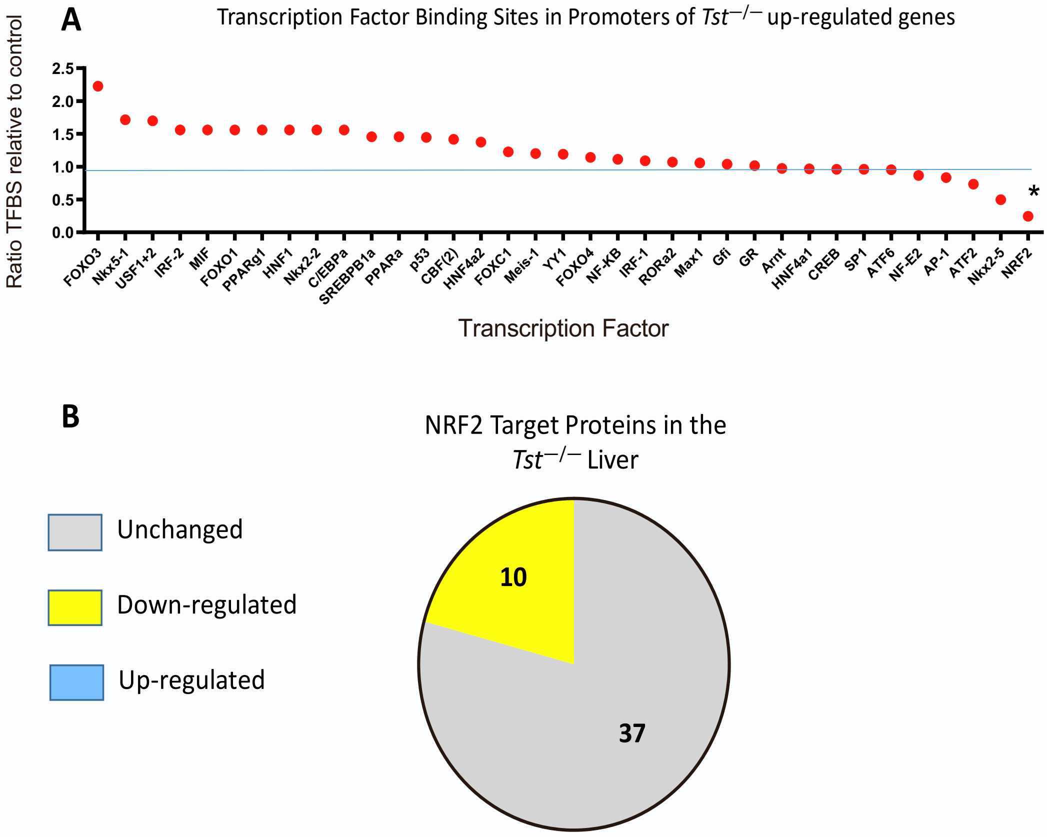

Figure S6. Hepatic proteins enriched in *Tst* $^{-/-}$ mice show under-representation of NRF2 promoter binding sites. Related to Figure 3 and Table 3. (**A**) abundance in *Tst*$^{-/-}$ liver compared to a control set of proteins that are unchanged between 6J and *Tst*$^{-/-}$. The proportion of genes containing a promoter binding site from proteins increased in *Tst*$^{-/-}$ was divided by the proportion of genes containing a binding site from a control set of genes. (**B**) Pie charts representing the number of NRF2-target proteins whose abundance is increased (blue), decreased (yellow) or unchanged (grey) in the Tst$^{-/-}$ liver. Significance of transcription factor enrichment analysis was calculated using a Fishers Exact test. * $P < 0.05$. Significance for NRF2 target abundance was performed with the Freeman-Halton Fishers Exact Test.

Figure S7. Hepatocyte respiration after high–fat feeding or after amino acid or pyruvate challenge is comparable between C57Bl/6J and *Tst*$^{-/-}$ mice *in vitro*. Related to Figure 4.

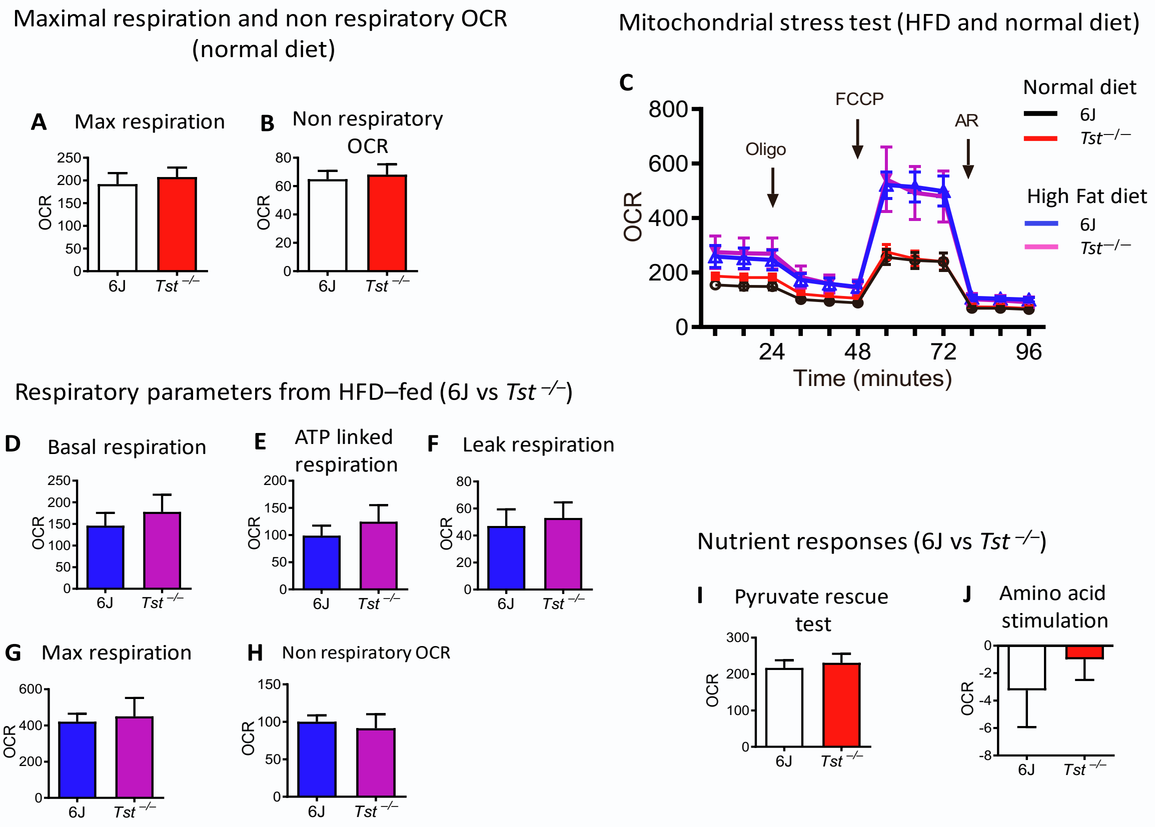

Figure S7. Hepatocyte respiration after high–fat feeding or after amino acid or pyruvate challenge is comparable between C57Bl/6J and *Tst*$^{-/-}$ mice *in vitro*. Related to Figure 4. (**A**) Maximal respiratory OCR elicited by uncoupling with FCCP, by hepatocytes from normal diet-fed C57Bl/6J (n = 6) or *Tst*$^{-/-}$ (n = 6) mice, calculated from Figure 3B. (**B**) Non-respiratory OCR remaining following the inhibition of respiration with antimycin and rotenone, by hepatocytes from normal diet-fed C57Bl/6J (n = 6) or *Tst*$^{-/-}$ (n = 6) mice, calculated from Figure 3B. (**C**) Seahorse trace representing the mean oxygen consumption rate (OCR), normalised to protein, by hepatocytes from normal diet-fed (n = 6/genotype), and high fat diet-fed (n = 4/genotype) C57Bl/6J and *Tst*$^{-/-}$mice during a mitochondrial stress test. (**D**)Basal respiratory OCR linked to ATP production (antimycin/rotenone sensitive) by hepatocytes from high fat diet-fed C57Bl/6J (n = 4) or *Tst*$^{-/-}$ (n = 4) mice, calculated from Figure S4C. (**E**) Respiratory OCR linked to ATP production (oligomycin sensitive) by hepatocytes from high fat diet-fed C57Bl/6J (n = 4) or *Tst*$^{-/-}$ (n = 4) mice, calculated from Figure S4C. (**F**) Respiratory OCR relating to proton leak (oligomycin insensitive) by hepatocytes from high fat diet-fed C57Bl/6J (n = 4) or *Tst*$^{-/-}$ (n = 4) mice, calculated from Figure S4C. (**G**) Maximal respiratory OCR elicited by uncoupling with FCCP, by hepatocytes from high fat diet-fed C57Bl/6J (n = 4) or *Tst*$^{-/-}$ (n = 4) mice, calculated from Figure S4C. (**H**) Non-respiratory OCR remaining following the inhibition of respiration with antimycin and rotenone, by hepatocytes high fat diet-fed C57Bl/6J (n = 4) or *Tst*$^{-/-}$ (n = 4) mice, calculated from Figure S4C. (**I**) Stimulation of maximal uncoupled respiration following addition of pyruvate (2mM), from normal diet-fed C57Bl/6J (n = 4) or *Tst*$^{-/-}$ (n = 4) mice. (**J**) Stimulation of maximal uncoupled respiration following addition of aspartate (1mM) and glutamax (1mM), by hepatocytes from normal diet-fed C57Bl/6J (n = 1) or *Tst*$^{-/-}$ (n = 1) mice. Data are represented as mean ±SEM. Significance was calculated using an unpaired two tailed, student's t-test. * $P < 0.05$.

Table S1. Parameters during the euglycemic hyperinsulinemic clamp

(A) Parameters during the basal (pre clamp) experiment (60-90 minutes post tracer)

Parameter	6J chow	*Tst*$^{-/-}$ chow	6J HFD	*Tst*$^{-/-}$ HFD	Genotype	Diet
Fasted Glucose 60 min (mg/dl)	116.35± 14.93	135.72± 7.22	146.50± 3.89	167.13± 9.68	*	**
Glycolysis (mg/kg/min)	11.63 ± 1.60	11.12 ± 0.62	12.89 ± 0.57	12.61 ± 0.62	ns	ns (0.09)
Glycogen synthesis (mg/kg/min)	21.48 ± 2.06	19.02 ± 2.04	15.08 ± 2.76	16.28 ± 2.50	ns	****

(B) Measurements and parameters during the clamp experiment (160-210 minutes post tracer)

Parameter	6J chow	*Tst*$^{-/-}$ chow	Genotype (chow)	6J HFD	*Tst*$^{-/-}$ HFD	Genotype (HFD)
Glucose 160 min (mg/dl)	108.0 ± 5.0	121.0 ± 10.1	ns	120.1± 3.7	125.6 ± 10.3	ns
Glucose 170 min (mg/dl)	120.3 ± 6.4	129.8 ± 5.8	ns	118.1 ± 4.1	143.9 ± 14.0	ns (0.08)
Glucose 180 min (mg/dl)	115.0 ± 5.5	125.5 ± 2.5	ns (0.08)	125.9 ± 2.9	126.7 ± 6.8	ns
Glucose 190 min (mg/dl)	124.0 ± 1.0	131.0 ± 4.6	ns	121.5 ± 4.7	116.1 ± 3.7	ns
Glucose 200 min (mg/dl)	121.0 ± 5.0	125.3 ± 5.8	ns	121.9 ± 3.0	115.7 ± 5.3	ns
Glucose 210 min (mg/dl)	113.0 ± 7.8	123.5 ± 4.6	ns	119.4 ± 4.0	116.9 ± 2.7	ns
Glucose IR 160-210 min (mg/kg/min)	84.9 ± 4.2	85.0 ± 2.6	ns	70.26 ± 4.83	69.95 ± 4.96	ns
Glucose IR 160 (mg/kg/min)	82.3 ± 5.1	87.1 ± 3.2	ns	68.8 ± 4.2	68.9 ± 5.1	ns
Glucose IR 170 (mg/kg/min)	85.2 ± 4.9	89.0 ± 3.0	ns	70.0 ± 5.1	74.6 ± 4.4	ns
Glucose IR 180 (mg/kg/min)	84.7 ± 4.4	83.5 ± 4.2	ns	70.6 ± 5.1	70.7 ± 6.2	ns
Glucose IR 190 (mg/kg/min)	85.2 ± 4.0	86.1 ± 2.1	ns	69.9 ± 4.8	69.1 ± 5.7	ns
Glucose IR 200 (mg/kg/min)	85.2 ± 4.0	84.8 ± 2.4	ns	70.4 ± 4.7	71.2 ± 4.5	ns
Glucose IR 210 (mg/kg/min)	85.2 ± 4.0	84.5 ± 2.4	ns	70.4 ± 4.7	71.2 ± 4.5	ns
Turnover (mg/kg/min)	85.97 ± 3.52	94.50 ± 3.87	ns	73.61 ± 5.07	63.08 ± 7.10	ns
Hepatic Glucose Prod. (mg/kg/min)	1.10 ± 5.31	9.92 ± 9.15	ns	3.326 ± 4.03	-6.83 ± 7.93	ns
Glycolysis (mg/kg/min)	45.90 ± 2.218	48.37 ± 2.05	ns	42.85 ± 1.48	36.42 ± 4.62	ns (0.19)
Glycogen synthesis (mg/kg/min)	40.06 ± 4.44	46.12 ± 2.68	ns	30.76 ± 5.36	26.66 ± 4.49	ns
Integral Glucose (dpm.min/mg)	$3.6e^{7} \pm 1.4e^{6}$	$2.95e^{7} \pm 1.2e^{6}$	*	$1.88e^{7} \pm 1.4e^{6}$	$1.7e^{7} \pm 1.6e^{6}$	ns
IWAT glucose utilization (ng/mg.min)	14.21 ± 4.05	18.62 ± 2.04	ns	4.53 ± 1.07	6.64 ± 1.00	ns
EWAT glucose utilization (ng/mg.min)	7.523 ± 4.39	7.21 ± 2.18	ns	2.94 ± 0.56	3.64 ± 0.39	ns
VL glucose utilization (ng/mg.min)	33.77 ± 2.98	38.66 ± 1.70	ns (0.17)	49.65 ± 9.19	47.42 ± 9.19	ns
EDL glucose utilization (ng/mg.min)	35.79 ± 11.09	40.86 ± 10.25	ns	68.79 ± 8.65	61.29 ± 11.80	ns
Soleus glucose utilization (ng/mg.min)	99.85 ± 12.38	123.60 ± 13.90	ns (0.13)	220.8 ± 24.45	198.5 ± 32.92	ns
Tibialis glucose utilization (ng/mg.min)	47.25 ± 7.22	53.67 ± 4.40	ns	74.39 ± 6.46	80.16 ± 6.09	ns
Heart glucose utilization (ng/mg.min)	161.40 ± 7.65	198.00 ± 14.09	ns (0.13)	226.6 ± 51.01	262.5 ± 23.29	ns
Liver glucose utilization (ng/mg.min)	3.53 ± 0.56	3.57 ± 0.63	ns	3.378 ± 0.39	3.58 ± 0.46	ns
End Clamp Insulin (µU/ml)	133.6 ± 7.03	126.4 ± 5.06	ns	147.2 ± 9.4	124.4 ± 10.9	ns

*** P < 0.05, ** P < 0.01, *** P < 0.001, **** P < 0.0001**

Table S1. Parameters during the euglycemic hyperinsulinemic clamp. Related to Figure 1. Metabolic parameters measured during continuous trace infusion but prior to clamp (**A**) and during maintenance of euglycemia and hyperinsulinemia (**B**) from C57Bl/6J (chow-fed, n = 3, hfd-fed, n = 8) and *Tst*$^{-/-}$ (chow-fed, n = 6, hfd-fed, n = 7) mice. Data are represented as mean ±SEM. Significance for the basal experiment (**A**) was

calculated using a 2-WAY ANOVA for *genotype* and *diet*. Significance for the clamp (**B**) was calculated for each diet separately using T-tests for *genotype*. * $P < 0.05$, ** $P < 0.01$, *** $P < 0.001$, **** $P < 0.0001$

Table S2. Hydrogen sulfide disposal by hepatocytes and mitochondria (Amperometry)			
nmoles/min/mg protein	**C57Bl/6J**	***Tst***$^{-/-}$	**Significance**
Hepatocytes	3.88 +/- 0.095	4.15 +/- 0.345	**ns**
Hepatocytes (Respiratory)	1.97 +/- 0.176	2.91 +/- 0.288	*
Hepatocytes (Non-respiratory)	1.91 +/- 0.181	1.24 +/- 0.117	*
Liver Mitochondria	0.65 +/- 0.095	1.23 +/- 0.129	*
Liver Mitochondria (Respiratory)	0.26 +/- 0.060	0.50 +/- 0.080	*

*** P < 0.05**

Table S2. *Tst* deletion results in increased respiratory H_2S disposal by hepatocytes. Related to Figure 2 and Table 1. H_2S disposal rates (measured by gas selective amperometry following addition of 10 µM Na_2S) of hepatocytes (n = 6/genotype), or isolated liver mitochondria (n = 7/genotype) of ND-fed C57Bl/6J and *Tst*$^{-/-}$mice. Rates of H_2S disposal were measured with and without respiratory inhibition following addition of Antimycin (2µM). Antimycin insensitive disposal rates are referred to as non-respiratory. The Antimycin sensitive disposal rates are referred to as respiratory. Data are represented as mean ±SEM. Significance was calculated using paired two-tailed student's t-test. * P < 0.05.

Table S4. Sulfide metabolism proteins in liver proteome ($Tst^{-/-}$ vs C57Bl/6J, ND-fed)				
**Feature ID**	**Name**		**Fold Change**	**Significance**
Q3UW66	MPST	Mercaptopyruvate sulfurtrasferase	1.27	**
Q8R086	SUOX	Sulfite Oxidase	1.06	Ns
Q3UDS4	SQOR	Sulfide quinone reductase-like	1.06	Ns
Q91WT9	CBS	Cystathionine beta-synthase	1.03	Ns
Q9DCM0	ETHE1	Ethylmalonic encephalopathy 1	-1.01	Ns
Q8VCNS	CTH	Cystathionine gamma-lyase	-1.02	Ns

*** Raw P < 0.05, ** Adjusted P < 0.05**

Table S4. *Tst* deletion selectively regulates MPST in the sulfide pathway of normal diet–fed mice. Related to Figure 3 and Table 3. Relative peptide abundance of proteins of the sulfide production and disposal pathway from the liver proteome of normal diet (ND) fed mice. 'Fold Change' indicates the relative abundance of the protein in $Tst^{-/-}$ relative to C57Bl/6J.

Table S5. GO terms - Nutrient metabolism; reduced in ND *Tst*$^{-/-}$ liver (*Tst*$^{-/-}$ vs C57Bl/6J liver, ND-fed)

GO-ID	Name	Genes	Significance
0006629	Lipid metabolic process	19	**
0006631	Fatty acid beta-oxidation	7	**
0003995	Acyl-CoA dehydrogenase activity	3	*
0047617	Acyl-CoA hydrolase activity	2	*

*** P < 0.05, ** P < 0.01**

Table S5. *Tst* Deletion results in reduction of selective fatty acid specific GO terms. Related to Figure 3 and Table 3. Significant GO terms (glucose or lipid related) represented by proteins that are less abundant in the ND-fed *Tst*$^{-/-}$ liver compared with ND-fed C57Bl/6J. 'Genes' indicates the number of genes in the *Tst*$^{-/-}$ that represent the changes driving the GO term.

Table S6 Insulin regulated proteins in *Tst*$^{-/-}$ and C57Bl/6J mice

(A) Abundance of peptides of insulin–induced proteins (*Tst*$^{-/-}$ vs C57Bl/6J, ND-fed)

Feature ID	Name	Fold change	Significance
Q3UGT1	CPT1A	1.03	Ns
P19096	FASN	-1.04	Ns
Q3UDA8	CPT2	-1.05	Ns
Q3V2G1	APOA1	-1.10	Ns
Q5SVI5	GCK	-1.14	*

(B) Abundance of peptides of insulin–suppressed proteins (*Tst*$^{-/-}$ vs C57Bl/6J, ND-fed)

Feature ID	Name	Fold change	Significance
QO5421	CYP2E1	1.13	**
Q9D6M3	SLC25AA2	-1.03	Ns
Q8CI37	PCK1	-1.09	Ns
Q3UJ70	HMGCS1	-1.09	Ns
O08601	MTTP	-1.20	**

(C) Abundance of peptides of insulin–induced proteins (*Tst*$^{-/-}$ vs C57Bl/6J, High Fat-fed)

Feature ID	Name	Fold change	Significance
Q3UGT1	CPT1A	1.03	Ns
P19096	FASN	-1.04	Ns
Q3UDA8	CPT2	-1.05	Ns
Q3V2G1	APOA1	-1.1	Ns
Q5SVI5	GCK	-1.14	*

(D) Abundance of peptides of insulin–suppressed proteins (*Tst*$^{-/-}$ vs C57Bl/6J, High Fat-fed)

Feature ID	Name	Fold change	Significance
QO5421	CYP2E1	-1.05	Ns
Q9D6M3	SLC25AA2	-1.01	Ns
Q8CI37	PCK1	1.09	Ns
Q3UJ70	HMGCS1	-1.04	Ns
O08601	MTTP	1.09	*

*** Raw P < 0.05, ** Adjusted P < 0.05**

Table S6. Proteins regulated by insulin are broadly comparable in expression between *Tst*$^{-/-}$ and C57Bl/6J. Related to Figure 3. Relative abundance in proteins that are known to be induced (A) or suppressed

(B) by insulin in the liver, from the liver proteome of normal diet fed mice. 'Fold Change' indicates the relative abundance of the protein in $Tst^{-/-}$ relative to C57Bl/6J. Relative abundance in proteins that are known to be induced (C) or suppressed (D) by insulin in the liver, from the liver proteome of high fat diet fed mice. 'Fold Change' indicates the relative abundance of the protein in $Tst^{-/-}$ relative to C57Bl/6J.

Table S7. KEGG pathways shared by high fat feeding and TST deletion			
**Entry**	**Name**	**Comparison**	**Significance**
**A. Shared up-regulated pathways**			
00260	Glycine, serine and threonine metabolism	*Tst*$^{-/-}$ vs 6J	**
		HFD vs ND	**
**B. Shared down-regulated pathways**			
00980	Metabolism of xenobiotics by cytochrome P450	*Tst*$^{-/-}$ vs 6J	****
		HFD vs ND	*
00982	Drug metabolism – cytochrome P450	*Tst*$^{-/-}$ vs 6J	****
		HFD vs ND	*
04142	Lysosome	*Tst*$^{-/-}$ vs 6J	**
		HFD vs ND	****
04390	Hippo signaling pathway	*Tst*$^{-/-}$ vs 6J	**
		HFD vs ND	****
05215	Prostate cancer	*Tst*$^{-/-}$ vs 6J	**
		HFD vs ND	*
04024	cAMP signaling pathway	*Tst*$^{-/-}$ vs 6J	*
		HFD vs 6J	*
04141	Protein processing endoplasmic reticulum	*Tst*$^{-/-}$ vs 6J	*
		HFD vs 6J	**
05211	Renal cell carcinoma	*Tst*$^{-/-}$ vs 6J	*
		HFD vs 6J	*
04722	Neurotrophin signaling pathway	*Tst*$^{-/-}$ vs 6J	*
		HFD vs 6J	*
04110	Cell cycle	*Tst*$^{-/-}$ vs 6J	*
		HFD vs 6J	**
04918	Thyroid hormone synthesis	*Tst*$^{-/-}$ vs 6J	*
		HFD vs 6J	***
04612	Antigen processing and presentation	*Tst*$^{-/-}$ vs 6J	*
		HFD vs 6J	**
*** P < 0.05, ** P < 0.01, *** P < 0.001 **** P < 0.0001**			

Table S7. KEGG Pathways shared by high fat feeding and TST deletion. Related to Figure 3. (**A**) KEGG pathways that are significantly up-regulated in the same direction by both high fat diet (HFD vs ND), and *Tst* deletion (*Tst*$^{-/-}$ vs C57Bl/6J). (**B**) KEGG pathways that are significantly down-regulated in the same direction by both high fat diet (HFD vs ND), and *Tst* deletion (*Tst*$^{-/-}$ vs C57Bl/6J). 'Comparison' indicates the two groups being compared.

Table S8. Effect of high fat feeding on sulfide pathway proteins (High fat diet vs ND-fed)					
**Feature ID**	**Name**	**Fold change in 6J**	**Significance**	**Fold change in *Tst*$^{-/-}$**	**Significance**
Q3UW66	MPST	1.35	**	1.15	*
Q8R086	SUOX	1.21	**	1.23	**
Q545S0	TST	1.19	Ns	n/a	n/a
Q8VCNS	CTH	-1.04	Ns	-1.06	Ns
Q9DCM0	ETHE1	-1.05	Ns	1.03	Ns
Q3UDS4	SQOR	-1.08	Ns	1.01	Ns
Q91WT9	CBS	-1.10	*	-1.06	Ns

*** Raw P < 0.05, ** Adjusted P < 0.05**

Table S8. Effect of high fat feeding on the sulfide pathway of C57Bl/6J and *Tst*$^{-/-}$ mice. Related to Figure 3. Protein abundances of the sulfide production and disposal pathway from the liver proteome of C57Bl/6J and *Tst*$^{-/-}$ mice. 'Fold Change' indicates the relative abundance of the protein in high fat diet fed mice relative to normal diet fed mice, shown separately for each genotype.

Table S9. Pathways in *Tst*$^{-/-}$ that are regulated oppositely to high fat feeding

Entry	Name	Comparison	Direction
**A**	**KEGG Pathways**		
00980	Metabolism of xenobiotics by cytochrome P450	*Tst*$^{-/-}$ vs 6J	Decreased
		HFD vs ND	Increased
00983	Drug metabolism – other enzymes	*Tst*$^{-/-}$ vs 6J	Decreased
		HFD vs ND	Increased
00053	Ascorbate and aldarate metabolism	*Tst*$^{-/-}$ vs 6J	Decreased
		HFD vs ND	Increased
00040	Pentose and glucoronate interconversions	*Tst*$^{-/-}$ vs 6J	Decreased
		HFD vs ND	Increased
00830	Retinol metabolism	*Tst*$^{-/-}$ vs 6J	Decreased
		HFD vs ND	Increased
**B**	**GO Terms**		
0006629	Lipid metabolic process	*Tst*$^{-/-}$ vs 6J	Decreased
		HFD vs ND	Increased
0006631	Fatty acid beta-oxidation	*Tst*$^{-/-}$ vs 6J	Decreased
		HFD vs ND	Increased
0003995	Acyl-CoA dehydrogenase activity	*Tst*$^{-/-}$ vs 6J	Decreased
		HFD vs ND	Increased
0047617	Acyl-CoA hydrolase activity	*Tst*$^{-/-}$ vs 6J	Decreased
		HFD vs ND	Increased

Table S9. KEGG pathways and GO terms that are regulated in the opposite direction by high fat feeding compared to Tst deletion. Related to Figure 3. (**A**) KEGG pathways that are regulated in the opposite direction by high fat diet (HFD-fed C57Bl/6J vs ND-fed C57Bl/6J), to *Tst* deletion (ND-fed *Tst*$^{-/-}$ vs C57Bl/6J). (**B**) GO terms that are regulated in the opposite direction by high fat diet (HFD-fed C57Bl/6J vs ND-fed C57Bl/6J), to *Tst* deletion (ND-fed *Tst*$^{-/-}$ vs C57Bl/6J). 'Comparison' Indicates the two groups being compared. 'Direction' indicates whether the protein abundance is decreased or increased in the first group relative to the second.
