# Supplementary material for: The hepatic compensatory response to elevated systemic sulfide promotes diabetes
Source: Cell Rep. 2021 Nov 9;37(6):109958. doi: 10.1016/j.celrep.2021.109958 (PMC8595646; doi:10.1016/j.celrep.2021.109958)
Supplement: Document S2. Article plus supplemental information [file mmc3.pdf]

Cell Reports

Article

The hepatic compensatory response to elevated systemic sulfide promotes diabetes

Graphical abstract

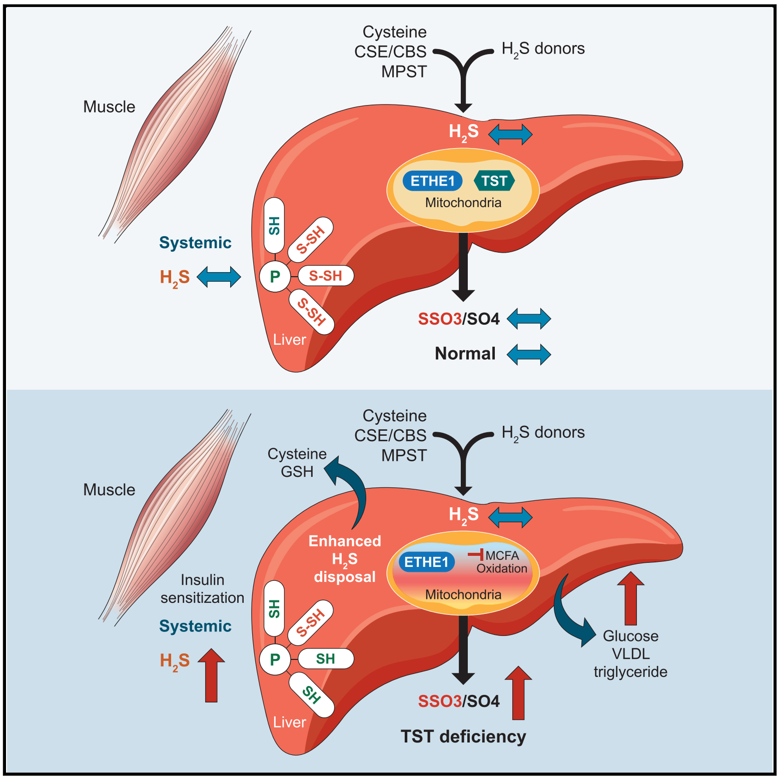

Authors

Roderick N. Carter, Matthew T.G. Gibbins, Martin E. Barrios-Llerena, ..., Colin Selman, Ruma Banerjee, Nicholas M. Morton

Correspondence

nik.morton@ed.ac.uk

In brief

Carter et al. show that mice lacking the mitochondrial sulfide oxidation pathway enzyme TST have high systemic sulfide levels that invoke an alternative hepatic sulfide disposal strategy. Consequently, hepatic metabolism is dominantly skewed toward a diabetogenic profile despite peripheral insulin sensitization. This has implications for sulfide donor therapeutic agents.

Highlights

- TST deficiency elevates sulfide, invoking exaggerated hepatic sulfide disposal
- Exaggerated sulfide disposal triggers global hepatic protein underpersulfidation
- Skewed persulfidation is associated with higher gluconeogenesis and impaired fat oxidation
- Diabetogenic hepatic metabolism dominates over apparent peripheral insulin sensitization

Carter et al., 2021, Cell Reports *37*, 109958
November 9, 2021 © 2021 The Authors.
https://doi.org/10.1016/j.celrep.2021.109958

CellPress

Cell Reports

CellPress
OPEN ACCESS

Article

The hepatic compensatory response to elevated systemic sulfide promotes diabetes

Roderick N. Carter,[1] Matthew T.G. Gibbins,[1,11] Martin E. Barrios-Llerena,[1,12] Stephen E. Wilkie,[1,2] Peter L. Freddolino,[3] Marouane Libiad,[3,13] Victor Vitvitsky,[3] Barry Emerson,[1,14] Thierry Le Bihan,[4,15] Madara Brice,[1] Huizhong Su,[5,16] Scott G. Denham,[1] Natalie Z.M. Homer,[1] Clare Mc Fadden,[1,17] Anne Tailleux,[6] Nourdine Faresse,[7,18] Thierry Sulpice,[7] Francois Briand,[7] Tom Gillingwater,[8] Kyo Han Ahn,[9] Subhankar Singha,[9,19] Claire McMaster,[10] Richard C. Hartley,[10] Bart Staels,[6] Gillian A. Gray,[1] Andrew J. Finch,[5,20] Colin Selman,[2] Ruma Banerjee,[3] and Nicholas M. Morton[1,21,*]

[1]University/British Heart Foundation Centre for Cardiovascular Science, University of Edinburgh, Queen's Medical Research Institute, Edinburgh EH16 4TJ, UK

[2]Glasgow Ageing Research Network (GARNER), Institute of Biodiversity, Animal Health and Comparative Medicine, University of Glasgow, Glasgow G12 8QQ, UK

[3]Department of Biological Chemistry, University of Michigan Medical School, Ann Arbor, MI 48109, USA

[4]SynthSys – Systems and Synthetic Biology, Edinburgh EH9 3JD, UK

[5]Cancer Research UK Edinburgh Centre, MRC Institute of Genetics & Molecular Medicine, University of Edinburgh, Western General Hospital, Edinburgh EH4 2XR, UK

[6]Université de Lille, INSERM, CHU Lille, Institut Pasteur de Lille, U101-EGID, 59000, Lille, France

[7]Physiogenex S.A.S, Prologue Biotech, 516 rue Pierre et Marie Curie, 31670 Labège, France

[8]College of Medicine & Veterinary Medicine, University of Edinburgh, Old Medical School (Anatomy), Teviot Place, Edinburgh EH8 9AG, UK

[9]Department of Chemistry, POSTECH, 77 Cheongam-Ro, Nam-Gu, Pohang, Gyungbuk 37673, South Korea

[10]School of Chemistry, Joseph Black Building, University of Glasgow, Glasgow G12 8QQ, UK

[11]Present address: Cambridge Biomedical Campus, Royal Papworth Hospital NHS Foundation Trust, Cardiology Department, Cambridge CB2 0AY, UK

[12]Present address: The International Clinical Research Centre, St. Anne's University Hospital, Brno 656 91, Czech Republic

[13]Present address: Institute for Integrative Biology of the Cell (I2BC), CEA, CNRS, Univ. Paris-Sud, Université Paris-Saclay, 91198 Gif-sur-Yvette Cedex, France

[14]Present address: BD Research Centre Ireland, Co. Limerick, Castletroy, Ireland

[15]Present address: Rapid Novor Inc., 44 Gaukel St., Kitchener, ON N2G 4P3, Canada

[16]Present address: Wellcome Trust Centre for Mitochondrial Research, Newcastle University, Newcastle NE2 4HH, UK

[17]Present address: Springer Nature Campus, London N1 9FN, UK

[18]Present address: D.I.V.A-expertise, 1 place Pierre Potier, 31100 Toulouse, France

[19]Present address: Institute of Advanced Studies and Research, JIS University, Kolkata 700091, India

[20]Present address: Cancer Research UK Barts Centre, London EC1M 6BQ, UK

[21]Lead contact

*Correspondence: nik.morton@ed.ac.uk

https://doi.org/10.1016/j.celrep.2021.109958

SUMMARY

Impaired hepatic glucose and lipid metabolism are hallmarks of type 2 diabetes. Increased sulfide production or sulfide donor compounds may beneficially regulate hepatic metabolism. Disposal of sulfide through the sulfide oxidation pathway (SOP) is critical for maintaining sulfide within a safe physiological range. We show that mice lacking the liver- enriched mitochondrial SOP enzyme thiosulfate sulfurtransferase (*Tst*$^{-/-}$ mice) exhibit high circulating sulfide, increased gluconeogenesis, hypertriglyceridemia, and fatty liver. Unexpectedly, hepatic sulfide levels are normal in *Tst*$^{-/-}$ mice because of exaggerated induction of sulfide disposal, with associated suppression of global protein persulfidation and nuclear respiratory factor 2 target protein levels. Hepatic proteomic and persulfidomic profiles converge on gluconeogenesis and lipid metabolism, revealing a selective deficit in medium-chain fatty acid oxidation in *Tst*$^{-/-}$ mice. We reveal a critical role of TST in hepatic metabolism that has implications for sulfide donor strategies in the context of metabolic disease.

INTRODUCTION

The prevalence of type 2 diabetes (T2D) continues to soar in parallel with that of obesity (World Health Organization, 2016). Increased hepatic glucose production and aberrant hepatic lipid metabolism are cardinal features of T2D (Consoli et al., 1989; Lewis et al., 2002). Dysregulation of hepatic nutrient metabolism in T2D is a promising area for therapeutic intervention because it precipitates the more severe liver pathologies that manifest along the spectrum of non-alcoholic fatty liver disease (NAFLD),

© 2021 The Authors.
This is an open access article under the CC BY license (http://creativecommons.org/licenses/by/4.0/).

steatosis, steatohepatitis, and hepatocellular carcinoma (Caron et al., 2011).

Hydrogen sulfide (hereafter referred to as sulfide), an endogenously produced gaseous signaling molecule (Abe and Kimura, 1996; Wang, 2012; Mishanina et al., 2015; Filipovic et al., 2017), has recently emerged as a modulator of nutrient metabolism (Desai et al., 2011; Szabo, 2011; Hine et al., 2015; Carter and Morton, 2016). Enzymatic sulfide production from sulfur amino acids is catalyzed by cystathionine beta synthase (CBS), cystathionine gamma lyase (CTH) (Chen et al., 2004; Singh et al., 2009), and 3-mercaptopyruvate sulfurtransferase (MPST) (Shibuya et al., 2009; Mikami et al., 2011; Yadav et al., 2013). Thioredoxin-mediated reduction of cysteine persulfides on proteins also regulates free sulfide and cysteine persulfide levels (Wedmann et al., 2016). Endogenously produced and exogenously administered sulfide specifically influences hepatic glucose and lipid metabolism (Mani et al., 2014; Pichette and Gagnon, 2016). Thus, *in vitro*, treatment of murine hepatocytes with sodium hydrosulfide (NaHS), or overexpression of rat *Cth* in HepG2 liver cells increased glucose production through increased gluconeogenesis and reduced glycogen storage (Zhang et al., 2013). Conversely, glucose production was lower in hepatocytes from *Cth* gene knockout ($Cth^{-/-}$) mice, which exhibit low sulfide production (Zhang et al., 2013). Elevation of sulfide with NaHS administration *in vivo* reduced cholesterol and triglyceride accumulation in the liver of high-fat diet (HFD)-fed mice (Wu et al., 2015). In contrast, inter-crossing of sulfide production-deficient $Cth^{-/-}$ mice with the hyperlipidemic $Apoe^{-/-}$ mouse strain ($Cth^{-/-}Apoe^{-/-}$) produced a phenotype of elevated plasma cholesterol following exposure to an atherogenic diet (Mani et al., 2013). Consistent with their higher cholesterol, $Cth^{-/-}Apoe^{-/-}$ mice developed fatty streak lesions earlier than $Apoe^{-/-}$ mice, and this effect was reversed by NaHS administration (Mani et al., 2013). Sulfide may also indirectly affect hepatic nutrient metabolism through its effect on hepatic artery vasorelaxation and, thus, liver perfusion (Fiorucci et al., 2005; Distrutti et al., 2008). The apparently beneficial effects of sulfide administration in multiple disease indications has led to a major drive toward development of targeted H_2S donor molecules as a therapeutic approach (Whiteman et al., 2011; Sestito et al., 2017). However, an often overlooked aspect of net sulfide exposure, key to the efficacy of therapeutic H_2S donors, is that it is regulated through its oxidative disposal. Thus, endogenous sulfide exposure is actively limited to prevent mitochondrial respiratory toxicity (Reiffenstein, 1992; Tiranti et al., 2009; Libiad et al., 2018). Sulfide is oxidized rapidly (Hildebrandt and Grieshaber, 2008; Norris et al., 2011) through the mitochondrial sulfide oxidation pathway (SOP), consisting of sulfide quinone oxidoreductase (SQOR), persulfide dioxygenase (ETHE1/PDO), and thiosulfate sulfurtransferase (TST; also known as rhodanese) (Hildebrandt and Grieshaber, 2008; Jackson et al., 2012; Libiad et al., 2014). The liver is highly abundant in SOP enzymes and is a major organ of whole-body sulfide disposal (Norris et al., 2011). Mice lacking the *Ethe1* gene ($Ethe1^{-/-}$) die of fatal sulfide toxicity (Tiranti et al., 2009), consistent with its critical role in sulfide oxidation and the severe pathological consequences of unchecked sulfide buildup in tissues. However, the importance of mitochondrial TST in the SOP *in vivo* remains obscure. In contrast to $Ethe1^{-/-}$ mice, $Tst^{-/-}$ mice were grossly normal despite exhibiting substantially elevated blood sulfide levels, as implied by qualitative measures (Morton et al., 2016). This revealed an important but distinct role of TST in the SOP *in vivo*. Nevertheless, $Tst^{-/-}$ mice showed an apparently diabetogenic impairment of glucose tolerance (Morton et al., 2016), consistent with the concept that increased sulfide promotes hepatic glucose production (Zhang et al., 2013). Because *Tst* deficiency is a model of chronic but viable sulfide elevation, determining the molecular mechanisms driving the aberrant metabolic profile can provide important insights into the optimal range for therapeutic sulfide exposure, particularly in light of the current interest in developing mitochondrially targeted sulfide donors (Gerő et al., 2016; Karwi et al., 2018). To this end, we sought to define the effect of *Tst* deficiency on the underlying molecular pathways that affect hepatic metabolism.

RESULTS

$Tst^{-/-}$ mice exhibit increased hepatic gluconeogenesis and dyslipidemia despite mild peripheral insulin sensitization

TST mRNA expression is highest in the liver (http://biogps.org/#goto=genereport&id=22117; tissue hierarchy of expression was validated in our own mouse substrain; Figure S1A). We therefore hypothesized that liver TST deficiency was the principal driver of the impaired glucose tolerance observed previously in $Tst^{-/-}$ mice (Morton et al., 2016). $Tst^{-/-}$ mice exhibited higher glucose levels than C57BL/6J controls in response to pyruvate challenge, consistent with higher hepatic glucose production (Figure 1A). We next tested phosphoenolpyruvate carboxykinase (PEPCK) activity, a key enzyme of *de novo* hepatic glucose synthesis, and found that it was higher in liver homogenates from $Tst^{-/-}$ mice (Figure 1B). Next we performed a 1-h $^{13}C_3$-pyruvate metabolite pulse incorporation experiment in isolated hepatocytes cultured in $^{12}C_3$-pyruvate-free medium. Hepatocytes from $Tst^{-/-}$ mice displayed ^{13}C labeling consistent with increased metabolism of pyruvate to oxaloacetate, a critical early step in gluconeogenesis. Specifically, aspartate, which is derived from pyruvate via oxaloacetate, was increased significantly in $Tst^{-/-}$ hepatocytes (Figure 1C). A trend toward higher $^{13}C_3$ malate and lower $^{13}C_2$ acetyl-coenzyme A (CoA) was also observed (Figures S1B and S1C). $^{13}C_3$ lactate was similar between genotypes, suggesting a similar activity of glycolytic disposal of pyruvate through lactate dehydrogenase (Figures S1B and S1C). Isotopologue distribution is shown in Figure S1C. Total pool sizes for all measured metabolites were similar between genotypes (Figure S1D). Although not a direct measure of glucose production, the data from *in vitro* hepatocytes suggested skewing of hepatocyte metabolism toward gluconeogenesis, and we therefore investigated this possibility. Indeed, consistent with increased endogenous glucose production in $Tst^{-/-}$ mice, fasting plasma glucose was higher in $Tst^{-/-}$ mice relative to 6J mice during the pre-clamp 3-^{3}H glucose tracer infusion phase (60–90 min after the tracer) of euglycemic, hyperinsulinemic (EH) clamp experiments (Figure 1D; Table S1A). Higher plasma glucose levels in $Tst^{-/-}$ mice under these conditions was not explained by lower glucose utilization in $Tst^{-/-}$ mice; glycogen synthesis and glycolysis were comparable between

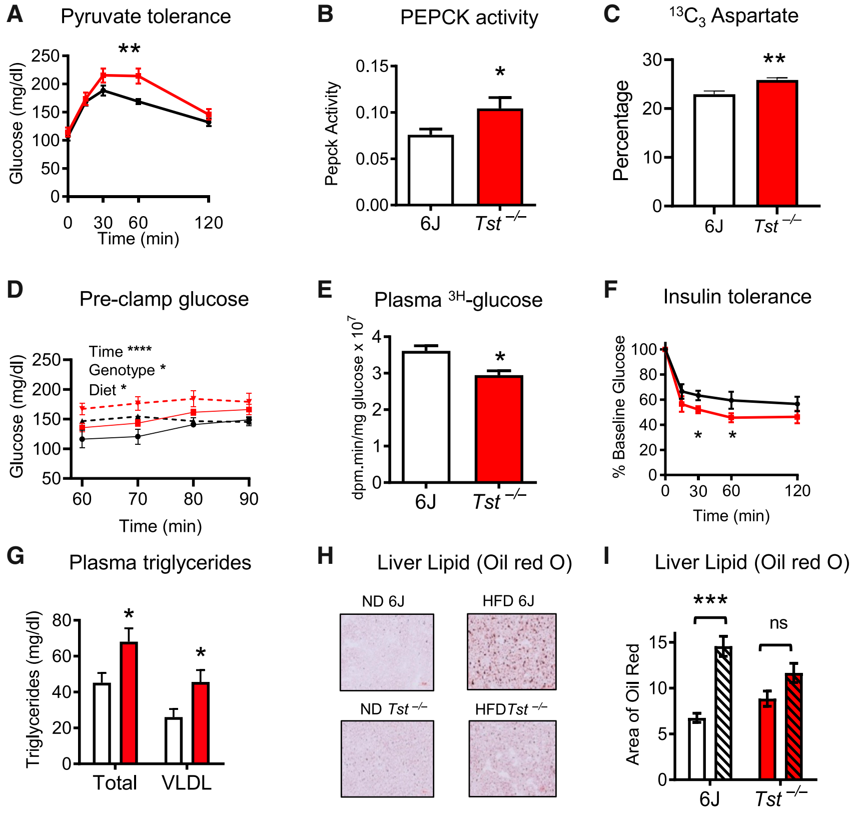

Figure 1. *Tst* deletion results in impaired glucose and lipid metabolism

(A) Plasma glucose over 120 min, following pyruvate (i.p., 1.5 mg/g) administration in overnight-fasted C57BL/6J (black line, n = 9) and $Tst^{-/-}$ (red line, n = 8) ND-fed mice.

(B) Extinction of NADH, measured by absorbance at 340 nm, coupled to PEPCK activity from liver homogenates taken from C57BL/6J (white bar, n = 6) and $Tst^{-/-}$ (red bar, n = 6) ND-fed mice.

(C) Production of ^{13}C (M+3) aspartate generated after a 1-h pulse of 1 mM 3-carbon labeled ^{13}C (M+3) pyruvate in ^{12}C pyruvate-free medium, expressed as a percentage of the total amount of detected metabolite, in primary hepatocytes from C57BL/6J (white bars, n = 6) and $Tst^{-/-}$ (red bars, n = 5) ND-fed mice.

(D) Blood glucose during the pre-clamp phase of the EH clamp from C57BL/6J (black lines) and $Tst^{-/-}$ (red lines) mice fed a control (ND, solid lines, n = 3, 6) or high-fat diet (HFD, broken lines, n = 6, 7).

(E) Mean integrated radioactive glucose (inversely related to whole-body glucose uptake) during a EH clamp from ND-fed C57BL/6J control (white, n = 3) and $Tst^{-/-}$ (red, n = 6) mice.

(F) Plasma glucose, expressed as percent of baseline glucose, over 120 min following insulin (i.p., 1 mU/g) administration in 4-h-fasted C57BL/6J (black line, n = 8) and $Tst^{-/-}$ (red line, n = 7) ND-fed mice.

(G) HPLC-quantified total and VLDL plasma triglyceride in 4-h-fasted C57BL/6J (white bar, n = 6) and $Tst^{-/-}$ (red bar, n = 6) ND-fed mice.

(H) Representative light microscopy images of liver sections stained with oil red O from normal diet (ND)-fed or HFD-fed C57BL/6J and $Tst^{-/-}$ mice. Magnification is 40X.

(I) Analysis of the area of red staining (oil red O) after thresholding, using ImageJ, from ND-fed (no pattern, n = 3–4/genotype) or HFD-fed (hatched pattern, n = 4–5/genotype) C57BL/6J (white bars) and $Tst^{-/-}$ (red bars) mice.

Data are represented as mean ± SEM. Significance was calculated using repeated-measures ANOVA (A and F), 2-way ANOVA (I), 3-way repeated-measures ANOVA (D), or unpaired two-tailed Student's t test (B, C, E, and G); *p < 0.05, **p < 0.01, ***p < 0.001, ****p < 0.0001. For (D), significant effects of time (****), diet (*) and genotype (*) were found. For (F), the analysis was performed on absolute glucose values and demonstrated a significant effect of time (****) and an interaction between time and genotype (*). t tests revealed that the decrement of glucose from baseline 30 and 60 min after insulin was greater in $Tst^{-/-}$ mice (*). For (I), no main genotype effect was found, but a significant effect of diet (***) and an interaction (*) were found. Post hoc analysis using Sidak's multiple comparison test shows an effect of diet on the 6J controls (***), whereas no effect of diet is found on $Tst^{-/-}$ mice. See also Figures S1 and S2 and Table S1.

genotypes across 60–90 min (Table S1A). Glucose turnover, a derived parameter used to infer glucose production, was also comparable between genotypes (Table S1A). However, derivation of glucose turnover requires that glucose levels are stable during the period in which it is calculated. In our pre-clamp baseline period, a highly significant effect of time (Figure 1D) indicated that this assumption was not met; thus, true endogenous glucose production cannot be inferred from the glucose turnover parameter in this instance. Combined with the pyruvate tolerance, PEPCK activity, and $^{13}C_3$-pyruvate pulse data, higher fasting glucose levels in $Tst^{-/-}$ mice, given comparable glucose utilization, are most likely due to higher endogenous glucose production.

We next wished to explore whether the changes to glucose metabolism were driven by insulin resistance. Liver glycogen, a marker of long-term carbohydrate storage typically impaired with insulin resistance, was comparable between $Tst^{-/-}$ and C57BL/6J control mice (Figure S2A). Despite unchanged steady-state markers of hepatic insulin sensitivity, impaired glucose tolerance, described previously in $Tst^{-/-}$ mice (Morton et al., 2016), suggested that whole-body, and usually hepatic, insulin resistance was present. We investigated this using the euglycemic clamp, where, unexpectedly, we observed whole-body insulin sensitization under these short-term steady-state conditions. During the clamp, when insulin was high and blood glucose levels were maintained constant, the glucose infusion rate was comparable between genotypes (Table S1B). However, an increase in whole-body glucose uptake (integral glucose) by tissues in $Tst^{-/-}$ mice was apparent (Figure 1E; Table S1B), supporting increased peripheral insulin sensitivity, with a directionally consistent trend for increased glucose uptake into several tissues. We confirmed this finding using standard insulin tolerance tests, where the glucose decrement in response to insulin was greater in $Tst^{-/-}$ mice (Figure 1F; Figure S2B). These data demonstrate a net increase in dynamic whole-body insulin sensitivity despite increased hepatic glucose output in $Tst^{-/-}$ mice. Finally, we assessed whole-body glucose homeostasis with the EH clamp method after chronic HFD feeding. Under these

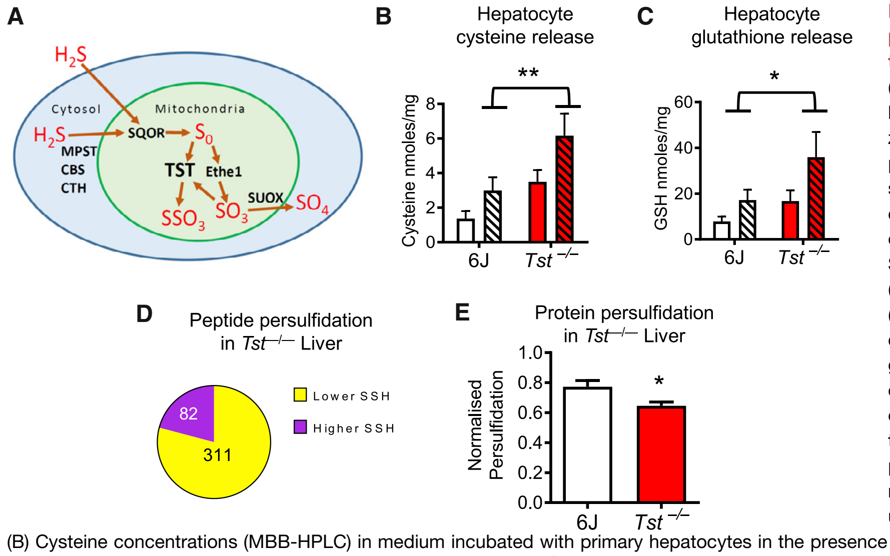

Figure 2. *Tst* deletion results in increased hepatic sulfur excretion and a reduction of protein persulfidation

(A) Schematic showing mammalian metabolism of hydrogen sulfide. The canonical production enzymes are shown in the cytosol. MPST, mercaptopyruvate sulfurtransferase; CBS, cystathionine beta synthase; CTH, cystathionine gamma lyase. Mitochondrial oxidation and disposal of hydrogen sulfide occurs through the SOP through the actions of SQOR (sulfide quinone oxidoreductase), ETHE1 (PDO), TST (thiosulfate sulfurtransferase), and SUOX (sulfite oxidase). These seven enzymes are major contributors to intracellular sulfide (and other inorganic sulfur) metabolism. For simplicity, the diagram does not include sulfide production, which can occur within mitochondria, or disposal pathways in the cytosol. The identity of oxidized sulfur species produced by SQOR remain disputed. The precise role of TST and other enzymes shown here remains under investigation.

(B) Cysteine concentrations (MBB-HPLC) in medium incubated with primary hepatocytes in the presence (hatched pattern) or absence (no pattern) of 1 mM methionine from C57BL/6J (white bars, n = 4/treatment) and $Tst^{-/-}$ (red bars, n = 4/treatment) mice.

(C) GSH concentrations (MBB-HPLC) in medium incubated with primary hepatocytes in the presence (hatched pattern) or absence (no pattern) of 1 mM methionine from C57BL/6J (white bars, n = 4/treatment) and $Tst^{-/-}$ (red bars, n = 4/treatment) mice.

(D) Pie chart depicting the proportion of liver peptides that are significantly higher (82 peptides, purple space) or lower (311 peptides, yellow space) in their persulfidation rate in $Tst^{-/-}$ (n = 3) relative to C57BL/6J (n = 3) mice.

(E) Total DTT-released cysteine-persulfidated liver protein as measured by REVERT total protein stain following western blotting, normalized to the total input protein of the sample from $Tst^{-/-}$ (red bar, n = 4) and C57BL/6J (white bar, n = 4) mice.

Data with error bars are represented as mean ± SEM. Significance was calculated using 2-way ANOVA (B and C) or Student's t test (E); *p < 0.05, **p < 0.01. For (B) and (C), the 2-way ANOVA reveals a main effect of genotype, indicated by * or ** on the histogram. A significant effect of methionine was also found for (B) and (C), not indicated on the histogram. For (D), peptides were selected as being significant at a P-diff of 0.95 or greater. See also Figure S3 and Table S2.

conditions, $Tst^{-/-}$ mice maintained increased hepatic glucose output (Figure 1D) but showed convergence of the insulin sensitivity profile with that of insulin-resistant C57BL/6J mice.

We also assessed whether *Tst* deficiency was associated with impaired lipid metabolism, another hallmark of diabetes. Fast protein liquid chromatography analysis of triglyceride levels and their lipoprotein distribution revealed significantly higher total plasma triglycerides in $Tst^{-/-}$ mice (Figure 1G). The higher triglyceride was selectively associated with an increased very low density lipoprotein (VLDL) triglyceride fraction (Figure 1G), consistent with a dominant liver-driven impairment in lipid metabolism (Mason, 1998). Total and distinct lipoprotein fraction plasma cholesterol levels were similar between genotypes (Figures S2C and S2D), suggestive of a triglyceride-selective effect of *Tst* deficiency on hepatic lipid efflux. HFD feeding significantly increased the liver lipid content of C57BL/6J mice but did not further increase the elevated lipid levels in the liver of $Tst^{-/-}$ mice (Figures 1H and 1I).

TST deficiency elicits compensatory hepatic sulfide disposal mechanisms that drive reduced global protein persulfidation

A role of TST in disposal of sulfide has been suggested by its participation in the SOP (Hildebrandt and Grieshaber, 2008; Libiad et al., 2014) and supported *in vivo* by the qualitatively higher blood sulfide of $Tst^{-/-}$ mice (Morton et al., 2016), shown schematically in Figure 2A. Here we quantified circulating sulfide, showing an approximately 10-fold elevation in the blood and plasma of $Tst^{-/-}$ mice (Table 1). Thiosulfate, an oxidized metabolite of sulfide (Vitvitsky et al., 2015, 2017) and a TST substrate (Banerjee et al., 2015), was approximately 20-fold higher in the plasma (Table 1) and profoundly higher (450-fold) in the urine (Table 1) of $Tst^{-/-}$ mice compared with C57BL/6J mice. Reduced glutathione (rGSH) levels were ~2-fold higher in the plasma of $Tst^{-/-}$ mice (Table 1). To determine any direct hepatic contribution to the elevated systemic sulfide *in vivo*, whole blood was sampled from the inferior vena cava (IVC) (Table 1). IVC sulfide levels tended to be higher in $Tst^{-/-}$ mice, but the magnitude of the increase (~3-fold) did not parallel that in trunk blood (~10-fold), suggesting that the liver was not a major source of the elevated circulating sulfide. Surprisingly, liver homogenate sulfide, thiosulfate, cysteine, and GSH levels were similar between $Tst^{-/-}$ and C57BL/6J mice (Table 1). Further, cultured hepatocytes from $Tst^{-/-}$ and C57BL/6J mice exhibited similar intracellular sulfide levels, as estimated using P3, a sulfide-selective fluorescent probe (Singha et al., 2015; Table 1). Mitochondrial sulfide levels in the liver, reported by MitoA/MitoN (Arndt et al., 2017), were similarly unchanged between genotypes (Table 1). The apparently unaltered hepatic steady-state sulfide levels, despite higher circulating sulfide, suggested that a profound homeostatic mechanism was invoked in the liver of $Tst^{-/-}$ mice. We assessed respiratory sulfide disposal (antimycin sensitive) and found that this was increased markedly in hepatocytes from $Tst^{-/-}$ mice, whereas antimycin-insensitive sulfide disposal was relatively reduced compared with hepatocytes from C57BL/6J mice (Table S2). Isolated liver mitochondria from $Tst^{-/-}$ hepatocytes also exhibited a higher sulfide disposal rate (Table S2). In addition, cysteine and GSH were excreted at higher

Table 1. Sulfur species in blood, urine, tissue, and cells

	C57BL/6J	$Tst^{-/-}$	$Tst^{-/-}$/6J ratio	Significance
Trunk blood (micromolar)[a]				
MBB-S (sulfide)	2.28 ± 0.43	22.18 ± 0.85	9.73	****
MBB-SSO3 (thiosulfate)	N/D	6.25 ± 3.17	n.c.	ns
Trunk plasma (micromolar)[b]				
MBB-S (sulfide)	1.88 ± 0.64	24.50 ± 2.02	13.03	****
MBB-SSO3 (thiosulfate)	3.99 ± 0.99	80.29 ± 13.6	20.12	**
MBB-GSH (reduced GSH)	48.0 ± 1.15	86.25 ± 6.27	1.80	***
Urine (micromoles/creatine/24 h)[c]				
MBB-SSO3 (thiosulfate)	4.99 ± 2.6	2374 ± 319	475.75	****
IVC (micromolar)[d]				
MBB-S (sulfide)	1.22 ± 0.20	3.58 ± 0.87	2.93	ns (0.08)
MBB-SSO3 (thiosulfate)	6.58 ± 4.51	88.3 ± 13.0	13.42	*
Liver (micromoles/kg wet liver)[e]				
MBB-S (sulfide)	13 ± 1	17 ± 3	1.31	ns
MBB-SSO3 (thiosulfate)	4 ± 1	15 ± 7	3.75	ns
DNFB-GSH (reduced GSH)	6,470 ± 380	6,850 ± 30	1.04	ns
DNFB-cysteine (cysteine)	82 ± 13	67 ± 11	0.82	ns
Sulfide P3 fluorescence (A510 nm/protein)[f]				
Hepatocyte	7.22 ± 1.00	7.89 ± 0.80	1.09	ns
Mitochondrial sulfide (MitoA)[g]				
Liver	0.78 ± 0.16	1.14 ± 0.45	1.46	ns

Tst deletion results in altered sulfur metabolites in blood and liver. Data are represented as mean ± SEM. Significance was calculated using unpaired two-tailed Student's t test. *$p < 0.05$, **$p < 0.01$, ***$p < 0.001$, ****$p < 0.0001$. MMB; monobromobimane.
[a]Sulfide dibimane and thiosulfate-MBB, measured by fluorescence detection following HPLC, from whole blood taken from trunk blood of ND-fed C57BL/6J (n = 4) and $Tst^{-/-}$ (n = 4) mice.
[b]Sulfide dibimane, thiosulfate-MBB, and rGSH-MBB, measured by fluorescence detection following HPLC, from EDTA-plasma of ND-fed C57BL/6J (n = 4) and $Tst^{-/-}$ (n = 4) mice.
[c]Thiosulfate-MBB corrected for creatinine from 24-h urine samples, taken from ND-fed C57Bl6/J (n = 4) and $Tst^{-/-}$ (n = 5) mice.
[d]Sulfide dibimane and thiosulfate-MBB from whole blood taken from the IVC downstream of the hepatic vein of ND-fed C57BL/6J (n = 3) and $Tst^{-/-}$ (n = 3) mice.
[e]Sulfide dibimane, thiosulfate-MBB, rGSH-MBB, and cysteine-MBB from whole liver (n = 4/genotype) of ND-fed C57BL/6J (n = 4) and $Tst^{-/-}$ (n = 4) mice.
[f]Fluorescence from cultured hepatocytes following incubation with P3 (sulfide reactive probe) from ND-fed C57BL/6J (n = 4) and $Tst^{-/-}$ (n = 4) mice.
[g]Ratio of MitoN/MitoA from the liver of ND-fed C57BL/6J (n = 5) and $Tst^{-/-}$ (n = 5) mice.

levels from $Tst^{-/-}$ hepatocytes under basal conditions and after stimulation of sulfur amino acid metabolism by addition of methionine (Figures 2B and 2C). Consistent with higher GSH turnover, hepatocytes from $Tst^{-/-}$ mice showed resistance to exogenous H_2O_2-mediated mitochondrial reactive oxygen species (ROS) production (Figure S3). We next determined the global hepatic protein persulfidation profile, the major post-translational modification mediated by sulfide (Krishnan et al., 2011; Kabil et al., 2014; Koike et al., 2017). Mass spectrometry analysis of maleimide-labeled liver peptides revealed a greater abundance of peptides with a lower persulfidation level (underpersulfidated) in the liver of $Tst^{-/-}$ mice (Figure 2D). We confirmed this using semiquantitative western blot analysis on pulled down maleimide-labeled proteins (Figure 2E). Gene Ontology (GO) analysis of underpersulfidated peptides (20 GO categories; Table 2) showed enrichment for "FAD-binding, methyl transferase, peroxisome, acyl-CoA dehydrogenase activity, and transaminase." Overpersulfidated peptides (8 GO categories; Table 2) were predominantly "nicotinamide metabolism." Pathway-specific peptide analysis showed a bias for over-persulfidation in gluconeogenesis proteins (Figure S4A) and a significantly higher magnitude of change (independent of direction of change) in persulfidation compared with global persulfidomic changes between C57BL/6J and $Tst^{-/-}$ mice (Figure S4B).

The hepatic proteome of $Tst^{-/-}$ mice reveals a distinct molecular signature of altered sulfur and mitochondrial nutrient metabolism

To gain molecular insight into the mechanisms underlying the apparently diabetogenic phenotype in $Tst^{-/-}$ mice, we compared hepatic proteomes of normal diet (ND)-fed mice. Kyoto Encyclopedia of Genes and Genomes (KEGG) analysis revealed 4 up-regulated pathways in the liver of $Tst^{-/-}$ mice related to amino acid metabolism, including sulfur amino acids, and

Table 2. *Tst* deletion results in differential persulfidation rate of liver proteins

GO ID	Name	Direction (*Tst*$^{-/-}$ versus 6J)	Genes
GO terms identified by log fold change			
0050660	FAD binding	decreased	12
0008168	methyltransferase activity	decreased	9
			9
0016741	transferase activity, transferring one-carbon groups	decreased	9
0008565	protein transporter activity	decreased	8
0008238	exopeptidase activity	decreased	7
0005777	peroxisome	decreased	7
0042579	microbody	decreased	7
0003995	acyl-CoA dehydrogenase activity	decreased	6
0008483	transaminase activity	decreased	6
0016769	transferase activity, transferring nitrogenous groups	decreased	6
0008757	S-adenosylmethionine-dependent methyltransferase activity	decreased	6
0016655	oxidoreductase activity, acting on NADH/NADPH, quinone	decreased	5
0004177	aminopeptidase activity	decreased	5
0000059	protein import into nucleus, docking	decreased	3
0005643	nuclear pore	decreased	3
0031965	nuclear membrane	decreased	3
0044453	nuclear membrane part	decreased	3
0046930	pore complex	decreased	3
0015629	actin cytoskeleton	decreased	3
0016652	oxidoreductase activity, NADH/NADPH, NAD/NADP acceptor	decreased	3
0050662	coenzyme binding	increased	5
			5
			4
			4
			4
			3
			3
0016651	oxidoreductase activity, NADH/NADPH,	increased	5
0003954	NADH dehydrogenase activity	increased	4
0008137	NADH dehydrogenase (ubiquinone) activity	increased	4
0050136	NADH dehydrogenase (quinone) activity	increased	4
0006739	NADP metabolism	increased	3
0006769	nicotinamide metabolism	increased	3
0006733	oxidoreduction coenzyme metabolism	increased	3

Shown are significant GO terms represented by peptides with different persulfidation rates in ND-fed *Tst*$^{-/-}$ mouse liver relative to C57BL/6J mice. "Direction" indicates whether persulfidation is decreased or increased in *Tst*$^{-/-}$ relative to C57BL/6J mice. "Genes" indicates the number of genes in *Tst*$^{-/-}$ mice that represent the changes driving the GO term.

sulfur metabolism (Table 3). GO analysis revealed 95 significantly up-regulated categories in the liver of *Tst*$^{-/-}$ mice (Table S3A). Among the top categories, 7 referred to amino acid metabolism and 1 referred to the organellar term "mitochondrion." KEGG analysis revealed 27 down-regulated pathways in the liver of *Tst*$^{-/-}$ mice (Table 3), including phase 1 and 2 detoxification pathways (cytochrome P450s, GSH, and glucuronidation) and "lysosome" and "protein processing in the endoplasmic reticulum" organellar terms. 213 GO terms were significantly down-regulated in *Tst*$^{-/-}$ mice (Table S4B). Among the most significant down-regulated terms were phase 2 detoxification "glutathione binding," "glutathione transferase activity," and "endoplasmic

Table 3. Protein abundance and persulfidation in ND-fed *Tst*$^{-/-}$ liver

Entry	Name	Genes	Significance
KEGG pathways increased in ND *Tst*$^{-/-}$ liver[a]			
00250	alanine, aspartate, and glutamate metabolism	6	***
00260	glycine, serine, and threonine metabolism	5	**
00270	cysteine and methionine metabolism	4	*
04122	sulfur relay system	2	*
KEGG pathways reduced in ND *Tst*$^{-/-}$ liver[b]			
00980	metabolism of xenobiotics by cytochrome P450	12	****
00982	drug metabolism – cytochrome P450	12	****
05204	chemical carcinogenesis	12	****
00480	glutathione metabolism	8	***
00040	pentose and glucoronate interconversions	5	**
04142	lysosome	6	**
04390	Hippo signaling pathway	4	**
00500	starch and sucrose metabolism	5	**
05215	prostate cancer	3	**
04024	cAMP signaling pathway	4	*
04141	protein processing in ER	9	*
05211	renal cell carcinoma	3	*
00830	retinol metabolism	6	*
00053	ascorbate and aldarate metabolism	4	*
00860	porphyrin and chlorophyll metabolism	4	*
04722	neurotrophin signaling pathway	3	*
04670	leukocyte transendothelial migration	4	*
04010	MAPK signaling pathway	4	*
04720	long-term potentiation	2	*
04914	progesterone-mediated oocyte maturation	2	*
04062	chemokine signaling pathway	3	*
04110	cell cycle	3	*
04015	Rap1 signaling pathway	4	*
00983	drug metabolism – other enzymes	5	*
04918	thyroid hormone synthesis	3	*
04612	antigen processing and presentation	3	*
05203	viral carcinogenesis	5	*
GO terms common to persulfidome and proteome in ND *Tst*$^{-/-}$ liver[c]			
GO ID	GO term	Persulfidation (Tst−/− versus 6J)	Abundance (Tst−/− versus 6J)
0008483	transaminase activity	decreased	increased
0016769	transferase activity, transferring nitrogenous groups	decreased	increased
0003995	acyl-CoA dehydrogenase activity	decreased	decreased
0005777	peroxisome	decreased	decreased
0042579	microbody	decreased	decreased

*p < 0.05, **p < 0.01, ***p < 0.001, ****p < 0.0001.
[a]Significant KEGG pathway terms represented by proteins that are more abundant in the liver of ND-fed *Tst*$^{-/-}$ compared with ND-fed C57BL/6J mice.
[b]Significant KEGG pathway terms represented by proteins that are less abundant in the liver of ND-fed *Tst*$^{-/-}$compared with ND-fed C57BL/6J mice. "Genes" indicates the number of genes in *Tst*$^{-/-}$ mice that represent the changes driving the KEGG pathway.
[c]GO terms that are significantly regulated at the level of cysteine persulfidation and protein abundance in the liver of ND-fed *Tst*$^{-/-}$compared with ND-fed C57BL/6J mice.

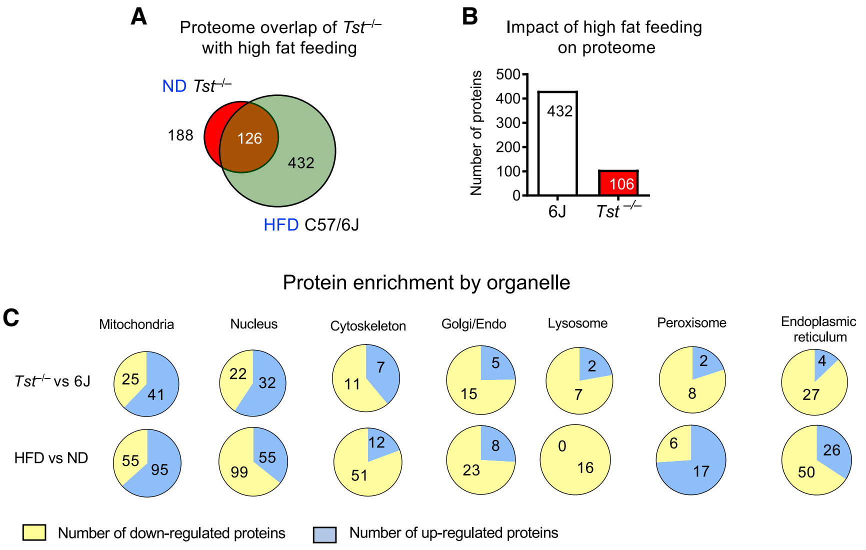

Figure 3. *Tst* deletion engenders a HFD feeding-like hepatic proteome with a distinct organellar signature

(A) Venn diagram representing the number of proteins significantly different (at $p < 0.01$) between ND-fed $Tst^{-/-}$ and C57BL/6J mice (red circle) and the number of regulated proteins between HFD-fed and ND-fed C57BL/6J (green circle) mice. The overlap (brown) represents proteins regulated in the same direction by both comparisons (n = 4/genotype).

(B) Number of proteins significantly different (at $p < 0.01$) between 58% HFD and ND in either C57BL/6J (white bar) or $Tst^{-/-}$ mice (red bar) (n = 4/genotype).

(C) Pie charts depicting the proportion of individual liver proteins that are upregulated (blue space) compared with downregulated (yellow space) after GO term categorization according to subcellular location. Top row: ND-fed $Tst^{-/-}$ relative to ND-fed C57BL/6J mice. Bottom row, HFD-fed C57BL/6J relative to ND-fed C57BL/6J mice. See also Table 3 and Figures S5 and S6.

reticulum'' categories. We validated the broadly consistent direction of change in a representative subset of proteins (Figures S5A and S5D). The most robust change we observed was increased MPST protein in whole liver (Figures S5A and S5D) and mitochondrial subfractions (Figures S5B and S5D). This change was remarkable because mRNA levels for *Mpst* were lower in $Tst^{-/-}$ mice (Figure S5C), likely as a result of loss of proximal *Mpst* promoter function; *Mpst* is a paralog of *Tst* (Nagahara, 2011) juxtaposed approximately 1 kb from the *Tst* gene. Protein levels for other sulfide-producing and disposal enzymes were comparable between genotypes (Table S4). A focused comparison of canonical proteins in glucose and lipid metabolism pathways (Table S5) revealed four GO categories that were down-regulated in $Tst^{-/-}$ mice: ''lipid metabolic process,'' ''fatty acid beta-oxidation,'' ''acyl-CoA dehydrogenase activity,'' and ''acyl-CoA hydrolase activity'' (Table S5). Canonical insulin-regulated proteins were largely comparable between genotypes (Table S6).

Hepatic protein expression in $Tst^{-/-}$ mice is consistent with lower NRF2 activation

We performed a transcription factor binding site (TFBS) enrichment analysis in the promoters of proteins that were up-regulated in the liver of $Tst^{-/-}$ mice to look for potential hub transcriptional drivers of the proteome profile (Figure S6A). This revealed a statistically significant under-representation of TFBS for the sulfide-responsive (Yang et al., 2013; Xie et al., 2016) NRF2 transcription factor (Figure S6A). Consistent with reduced hepatic NRF2 activation, 10 of 47 known NRF2-regulated proteins were lower in the liver of ND-fed $Tst^{-/-}$ mice compared with C57BL/6J mice (Figure S6B).

The proteome of TST deficiency versus HFD response in C57BL/6J mice reveals distinct regulation of lipid metabolism, sulfide metabolism, and detoxification pathways

We examined mechanistic commonalities between the diabetogenic hepatic phenotype of $Tst^{-/-}$ mice and that induced by the diabetogenic HFD feeding regimen in C57BL/6J mice. ND-fed $Tst^{-/-}$ mice were in a pre-existing diabetogenic state (Figure 1) that does not worsen with HFD feeding (Figures 1H and 1I; Table S1), suggesting gross phenotypic convergence of the two genotypes after HFD feeding. We compared the identity and direction of change of the 188 proteins differentially expressed in ND-fed $Tst^{-/-}$ mice (versus ND-fed C57BL/6J mice; Figure 3A) with proteins that were differentially expressed in response to HFD feeding in C57BL/6J mice (432 proteins; Figure 3A). There was a striking 67% overlap in individual proteins (126) in this comparison (Figure 3A). When we analyzed these two protein signatures for directionally shared pathways, one upregulated KEGG pathway, ''glycine, serine and threonine metabolism'' (Table S7A), and 12 downregulated KEGG pathways, including ''drug metabolism'' and ''endoplasmic reticulum'' (Table S8B), were common to the liver of ND-fed $Tst^{-/-}$ and HFD-fed C57BL/6J mice. Consistent with a pre-existing HFD-like proteome, the dynamic response to HFD in the liver of $Tst^{-/-}$ mice was muted relative to that observed in C57BL6J mice (106 proteins, a 4-fold lower response; Figure 3B). Focusing on the sulfide pathway, MPST and sulfite oxidase (SUOX) were increased by HFD feeding in C57BL/6J and $Tst^{-/-}$ mice (Table S8). The HFD-induced increase in MPST was less pronounced in the liver of $Tst^{-/-}$ mice, likely reflecting that it is already elevated in ND-fed $Tst^{-/-}$ mice. We then considered contrasting rather than congruent proteomics responses arising from TST deficiency versus HFD responses in C57BL/6J mice to illuminate potential novel pathways underlying the otherwise functionally similar diabetogenic hepatic $Tst^{-/-}$ phenotype. 5 KEGG pathways (Table S9A) and 4 GO terms (Table S9B) were regulated oppositely in this comparison. Strikingly, the GO terms were all related to lipid metabolism, which was up-regulated in the HFD response but down-regulated with TST deficiency (Tables S9A and S9B). An organelle-focused protein analysis showed shared upregulation of mitochondrial and endoplasmic reticulum pathways between TST deficiency (Figure 3C, top row) and C57BL/6J HFD responses (Figure 3C, bottom row) but a striking discordance in peroxisomal protein pathways (upregulated by HFD feeding

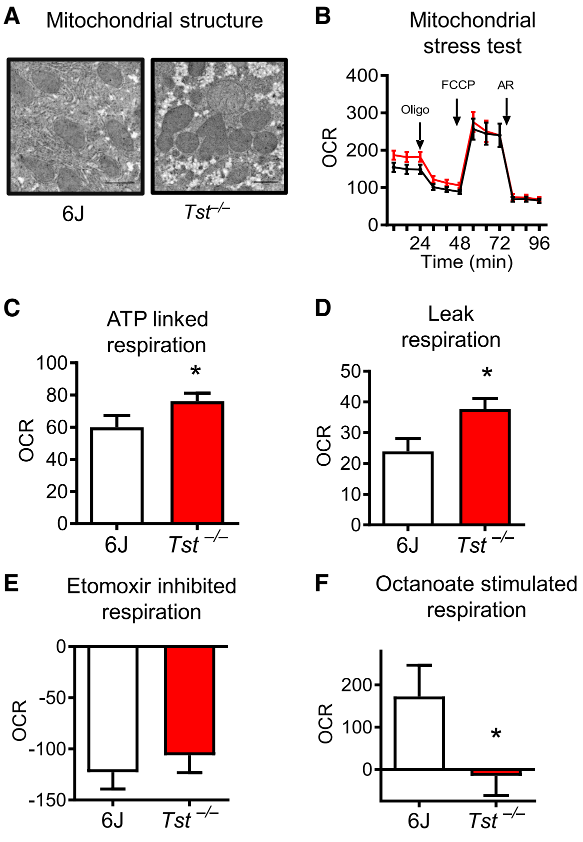

Figure 4. *Tst* deletion results in increased hepatocyte respiration but impaired medium-chain fat respiration

(A) Electron microscopy images of liver, visualizing mitochondria from ND-fed C57BL/6J (n = 4) or *Tst*$^{-/-}$ (n = 4) mice.

(B) Seahorse trace representing the mean oxygen consumption rate (OCR), normalized to protein, by hepatocytes from ND-fed C57BL/6J (n = 6) or *Tst*$^{-/-}$ (n = 6) mice during a mitochondrial stress test.

(C) Respiratory OCR linked to ATP production (oligomycin sensitive) by hepatocytes from ND-fed C57BL/6J (n = 6) or *Tst*$^{-/-}$ (n = 6) mice, calculated from (B).

(D) Respiratory OCR relating to proton leak (oligomycin insensitive) by hepatocytes from ND-fed C57BL/6J (n = 6) or *Tst*$^{-/-}$ (n = 6) mice, calculated from (B).

(E) Reduction of maximal uncoupled respiration following inhibition of LCFA mitochondrial import using etomoxir (8 μM) from ND-fed C57BL/6J (n = 4) or *Tst*$^{-/-}$ (n = 4) mice.

(F) Stimulation of maximal uncoupled respiration following addition of MCFA octanoate (250 μM) from ND-fed C57BL/6J (n = 4) or *Tst*$^{-/-}$ (n = 4) mice.

Data are represented as mean ± SEM. Significance was calculated using an unpaired two-tailed Student's t test (C–F); *$p < 0.05$. See also Figure S7.

and downregulated with TST deficiency) and nuclear proteins (downregulated by HFD feeding and upregulated with TST deficiency; Figure 3C).

The *Tst*$^{-/-}$ liver proteome and persulfidome converge on transamination and lipid oxidation pathways

To assess whether conservation of changes at the protein and post-translational modification levels can illuminate key regulatory hubs driving the hepatic phenotype, we ran a congruence analysis of the proteome and persulfidome. We found that the GO categories "amino acid," "lipid metabolism," and "peroxisome" were regulated significantly at the protein abundance and persulfidation levels in *Tst*$^{-/-}$ mice (Table 3).

Tst$^{-/-}$ hepatocytes exhibit elevated mitochondrial respiration and a defect in medium-chain fatty acid oxidation

Enhanced respiratory sulfide disposal was found from *Tst*$^{-/-}$ hepatocytes, and enrichment of mitochondrial proteins was suggested from the liver proteome of the *Tst*$^{-/-}$ mice (Table S4). We therefore sought to determine whether TST deficiency affected respiratory function and substrate utilization of the hepatocyte. Analysis of electron micrographs prepared from the liver of ND-fed *Tst*$^{-/-}$ mice and C57BL/6J controls showed morphologically normal mitochondria (Figure 4A). Basal respiration, comprising ATP-linked and leak respiration, was significantly higher in hepatocytes from *Tst*$^{-/-}$ mice (Figures 4B–4D). Maximal hepatocyte respiratory capacity and non-respiratory oxygen consumption were similar between genotypes (Figures S7A and S7B). In line with phenotypic convergence following HFD feeding, hepatocyte respiration was comparable between genotypes from HFD-fed mice (Figures S7C–S7H). A unique feature of the liver from *Tst*$^{-/-}$ mice was a decrease in proteins and persulfidation levels of proteins in lipid oxidation pathways. We therefore investigated hepatocyte respiration of lipids. Using a low-pyruvate (100 μM) medium to reveal respiratory dependency on other substrates, we showed that CPT1A-mediated mitochondrial oxidation of endogenous long-chain fatty acids (LCFAs; etomoxir inhibited) was similar between genotypes (Figure 4E). Next we bypassed CPT1A-mediated LCFA transfer and revealed a marked deficit in respiration stimulated by the medium-chain fatty acid octanoate in hepatocytes from *Tst*$^{-/-}$ mice (Figure 4F). A similar experiment adding back pyruvate revealed comparable stimulation of respiration between genotypes (Figure S7I). In amino acid-free medium, combined glutamine-, aspartate-, and alanine-stimulated hepatocyte respiration was comparable between genotypes (Figure S7J).

DISCUSSION

Elevated TST expression in adipose tissue has been identified as a genetic mechanism driving metabolically protective leanness in mice (Morton et al., 2016). Conversely, *Tst*$^{-/-}$ mice exhibited impaired glucose tolerance (Morton et al., 2016). However, *Tst*$^{-/-}$ mice had a subtle adipose tissue phenotype, suggesting a non-adipose origin for impaired glucose homeostasis. We found increased gluconeogenesis, steatosis, and elevated plasma VLDL triglycerides consistent with a predominantly hepatic origin for the diabetogenic phenotype. We cannot rule out a contribution of renal gluconeogenesis to the phenotype, and future work will address this limitation. Unexpectedly, and despite the markedly increased circulating sulfide levels (10-fold), the steady-state sulfide level was normal in the liver of *Tst*$^{-/-}$ mice. Moreover, we found evidence of multiple mechanisms for increased hepatic sulfide disposal, reduced downstream sulfide signaling, and associated underlying molecular links to an apparently diabetogenic phenotype. Our data suggest that the liver of *Tst*$^{-/-}$ mice has overshot in its attempt to

maximize hepatic sulfide removal, leading indirectly to detrimental metabolic consequences. This involves a combination of distinct compartmentalized cellular responses, including increased respiratory sulfide disposal and export of cysteine and GSH. Upregulation of translation and recruitment of MPST to mitochondria of *Tst*$^{-/-}$ mice is observed. This response, in the face of reduced transcription of *Mpst*, suggests a powerful post-transcriptional cellular sulfide-sensing mechanism. Interestingly, if MPST is compensating for TST-mediated sulfide disposal in this context, then it implies a subversion of normal MPST function away from sulfide production (Módis et al., 2013; Szabo et al., 2014; Kimura et al., 2017; Nagahara, 2018). Alternatively, this is a response to a perceived lower-sulfide environment. TST levels were also elevated in the liver of *Mpst*$^{-/-}$ mice, providing further support for a reciprocal compensatory mechanism between these two enzymes (Nagahara et al., 2019).

The unexpected finding of normal hepatic sulfide levels in *Tst*$^{-/-}$ mice led us to discover that the metabolic phenotype we observed was driven by the very mechanisms invoked to maintain sulfide within a normal range rather than sulfide excess per se. Several observations were consistent with this. For example, the major amino acid pathways increased in the liver of *Tst*$^{-/-}$ mice were transaminases involved in metabolism of GSH that support increased export of sulfur equivalents as GSH (and cysteine). These same transaminases support gluconeogenesis by redirecting Krebs cycle intermediates (Rui, 2014; Qian et al., 2015; Sookoian et al., 2016). Reprogramming of amino acid metabolism for sulfide disposal with knockon effects to drive hepatic glucose production are suggested, rather than any change to amino acid-linked mitochondrial respiration in hepatocytes. This is supported by the shift in hepatocyte pyruvate metabolism toward aspartate. In addition, glutathione S-transferases (GST) that inhibit gluconeogenesis (Ghosh Dastidar et al., 2018) were lower in the liver of *Tst*$^{-/-}$ mice. Further, activation of NRF2, which represses gluconeogenesis (Slocum et al., 2016) appears to be lower in the liver of *Tst*$^{-/-}$ mice. Involvement of NRF2 in the *Tst*$^{-/-}$ liver phenotype is further supported by the phenotype of *Nrf2*$^{-/-}$ mice that similarly exhibited steatohepatitis in the absence of insulin resistance (Meakin et al., 2014). However, NRF2 signaling can be complex and dependent on dietary context; *Nrf2*$^{-/-}$ mice showed improved glucose tolerance after HFD feeding (Zhang et al., 2012), suggesting that any contribution of a NRF2 signaling deficit in the liver of the *Tst*$^{-/-}$ mice changes upon HFD feeding. Beyond altered pyruvate flux, we also showed that hepatocytes of *Tst*$^{-/-}$ mice exhibited defective lipid metabolism. Specifically, medium-chain fatty acid (MCFA) oxidation was impaired, associated with selective reduction of the protein and persulfidation levels of lipid catabolic enzymes. This represents a mechanism linking altered sulfide metabolism to lipid oxidation, hepatic lipid accumulation, and dyslipidemia. Consistent with impaired MCFA oxidation defects as one driver of the phenotype, steatosis is observed in medium-chain acyl-CoA dehydrogenase (*Mcad*) $^{-/-}$ mice (Tolwani et al., 2005), and dyslipidemia is found in MCADD-deficient humans (Onkenhout et al., 1995). The data we present add to a growing understanding of the link between sulfide regulating genes and nutrient metabolism that has so far focused on the enzymes of sulfide production. Specifically, we provide support for the importance of the sulfide oxidizing pathway as a regulator of cellular sulfide exposure. Unexpectedly, the data reveal cellular mechanisms that are engaged to homeostatically regulate sulfide disposal and can affect cell energetics and nutrient metabolism.

Our findings may have implications for potentially unexpected side effects of sulfide donor therapeutic agents. In normal mice, *in vivo* sulfide administration for 4 weeks after HFD feeding partially reversed hepatic lipid accumulation invoked by chronic (16 weeks) HFD feeding (Wu et al., 2015). No evidence was provided regarding whether sulfide disposal mechanisms were altered (Wu et al., 2015). This efficacious subchronic sulfide administration regimen contrasts with our genetic model of chronic sulfide elevation as a driver of dysregulated metabolism and NAFLD. Clearly, the normal mice in the Na_2S administration studies had a fully functional SOP, suggesting that the presence of TST is required to achieve the beneficial metabolic effects of Na_2S administration. This is also consistent with the apparently low sulfide signaling status (evidenced by lower persulfidation and NRF2 target protein abundance) in the liver of the *Tst*$^{-/-}$ mice. The benefits of elevated sulfide cannot be realized, perhaps because a major mediator of those effects is missing, and the alternate mechanisms invoked do not fully compensate (e.g., MPST) or actively drive aberrant nutrient metabolism. Comparable studies of glucose and lipid metabolism after manipulation of other sulfide-regulating genes are limited. However, in a contrasting model of reduced sulfide production (*Cth*$^{-/-}$ mice), plasma triglycerides were lowered (Mani et al., 2013), opposite to what we observed with *Tst*$^{-/-}$ mice. The hepatic sulfide disposal status of the *Cth*$^{-/-}$ mouse model is unknown, but our findings predict suppression of the SOP to spare the limited endogenous sulfide produced. Intriguingly, they also predict a knockon effect on nutrient homeostasis because of reduced metabolic demand of the TST/SOP axis. A more direct model informing on the effects of impairment of the sulfide disposal pathway is deficiency of the key mitochondrial SOP enzyme ETHE1. *Ethe1*$^{-/-}$ mice suffer fatal sulfide toxicity (Tiranti et al., 2009); therefore, comparable metabolic studies are lacking. However, one notable observation is that *Ethe1*$^{-/-}$ mice have apparently 10-fold higher liver sulfide exposure than control mice (Tiranti et al., 2009), in contrast to the normalized hepatic sulfide levels of *Tst*$^{-/-}$ mice. Circulating sulfide levels were not reported for comparison, but the presumably relatively lower systemic sulfide levels of *Tst*$^{-/-}$ mice appear to have permitted an effective homeostatic sulfide disposal response in the liver to avoid toxicity, albeit with a metabolic cost. Consequently, the liver of *Tst*$^{-/-}$ mice has a functional and proteomics profile distinct from that of *Ethe1*$^{-/-}$ mice. For example, in the liver of *Tst*$^{-/-}$ and *Ethe1*$^{-/-}$ mice (Hildebrandt et al., 2013), proteins of the GST Mu type (GSTM) and peroxiredoxin (PRDX) families were altered, but sometimes in the opposite direction or with alteration of distinct protein subclasses. A notable difference is also observed in amino acid metabolism. The liver of *Ethe1*$^{-/-}$ mice exhibits increased expression of enzymes of branched-chain amino acid metabolism (Hildebrandt et al., 2013), distinct from the predominantly GSH-related amino acid pathways that are increased in the liver of *Tst*$^{-/-}$ mice. Beyond sulfide, TST may also have distinct cellular roles that affect metabolism,

such as mitoribosomal synthesis, ROS attenuation, and modulation of mitochondrial iron-sulfur clusters (Bonomi et al., 1977; Pagani and Galante, 1983; Nandi and Westley, 1998; Nandi et al., 2000; Smirnov et al., 2010).

Given the pro-diabetogenic liver phenotype in *Tst*$^{-/-}$ mice, its was surprising that insulin signaling in the liver appeared normal and peripheral insulin sensitivity was increased. There are precedents for increased hepatic glucose production independent of insulin resistance, as found in the *Nrf2*$^{-/-}$ mice (Meakin et al., 2014) and as driven by the transcription factor carbohydrate-response element-binding protein (ChREBP) (Uyeda and Repa, 2006; Kim et al., 2016). There is also evidence to support insulin-sensitizing effects of sulfide administration *in vivo* in mice and rats (Feng et al., 2009; Geng et al., 2013; Xue et al., 2013), consistent with sulfide-mediated insulin sensitization of non-hepatic tissues in *Tst*$^{-/-}$ mice. Higher circulating GSH in *Tst*$^{-/-}$ mice may also promote peripheral insulin sensitization (Jain et al., 2014; Lutchmansingh et al., 2018). Clearly, the net balance of glucose production from the liver and its peripheral disposal remain abnormal in *Tst*$^{-/-}$ mice. Indeed, the baseline metabolic phenotype of *Tst*$^{-/-}$ mice resembles in many ways that of a normal mouse fed a HFD, and we showed some overlapping pro-diabetogenic signatures between the liver proteome of *Tst*$^{-/-}$ mice and that of HFD-fed C57BL/6J mice. However, we also found distinct lipid metabolism and peroxisomal protein changes in *Tst*$^{-/-}$ mice. Unlike a HFD state, which is associated with dominant hepatic insulin resistance, the increased hepatic glucose production in ND-fed *Tst*$^{-/-}$ mice occurs despite normal hepatic insulin sensitivity. The significant changes in persulfidation of transaminase and gluconeogenesis proteins suggest that coordinated cross-talk across metabolic pathways underlies this atypical metabolic phenotype.

Sulfide donor therapeutic agents have been proposed as a clinical strategy for improving cardiovascular health (Szabó et al., 2011; Whiteman et al., 2011; Zhang et al., 2018). Elevated endogenous sulfide has also been implicated in the beneficial metabolic effects of caloric restriction (Miller et al., 2005; Hine et al., 2015, 2017, 2018; Shimokawa et al., 2015; Lee et al., 2016). Our results suggest that chronic sulfide elevation may have unintended detrimental consequences, driving liver glucose production and fat accumulation to undesirable levels. Fortunately, this may be limited to cases where SOP proteins are compromised through rare genetic effects, such as TST variants (Billaut-Laden et al., 2006; Libiad et al., 2015). More broadly, a number of drugs or supplements are known to increase cyanide, which may dominantly inhibit TST activity and result in secondary sulfide overexposure. These include nitroprusside (Morris et al., 2017) and amygdalin (Bromley et al., 2005; O'Brien et al., 2005). Indeed, the TST metabolite thiosulfate is commonly co-administered with nitroprusside to prevent cyanide toxicity (Curry et al., 1997). Furthermore, dietary and environmental exposure to cyanogenic compounds (Simeonova et al., 2004), e.g., smoking (Vinnakota et al., 2012) or cyanogenic diets (Kashala-Abotnes et al., 2019), may interfere with normal TST function and could lead to increased sensitivity to sulfide therapeutic agents. In contrast, we have shown that administration of the TST substrate thiosulfate can ameliorate diabetes (Morton et al., 2016), further underlining the potential utility of targeting the SOP in metabolic disease. As with all therapeutic strategies, a careful cost-benefit analysis is required. A comparable case of relevance are the statins, one of the most potent and widely used drugs to prevent atherosclerosis, which also carry a higher risk for diabetes (Swerdlow et al., 2015). The full effect of TST manipulation on opposing metabolic pathways requires further study. Our current study sheds light on the underlying hepatic mechanisms invoked for sulfide disposal that are relevant to current sulfide donor strategies and may inform on routes to reduce their potential metabolic side effects.

Limitations of the study

Although the liver is the site of most (~60%) post-absorptive gluconeogenesis in normal animals within physiological fasting ranges, renal/small intestinal gluconeogenesis begins to substantially contribute to circulating glucose with prolonged fasting/starvation (Sasaki et al., 2017; Mutel et al., 2011; Mithieux et al., 2003; Stumvoll 1998; Owen et al., 1969). We cannot rule out a role of renal or intestinal gluconeogenesis in the diabetogenic phenotype of *Tst*$^{-/-}$ mice. This will be an important area of future work, although liver TST is at least more than 3-fold that of the kidneys, and small intestinal TST is very low (BioGPS; Figure S1A).

STAR★METHODS

Detailed methods are provided in the online version of this paper and include the following:

- KEY RESOURCES TABLE
- RESOURCE AVAILABILITY
  - Lead contact
  - Materials availability
  - Data and code availability
- EXPERIMENTAL MODEL AND SUBJECT DETAILS
  - Experimental animals
  - Hepatocyte preparations
- METHOD DETAILS
  - Pyruvate tolerance test
  - PEPCK activity assay
  - ^{13}C Pyruvate metabolite tracing
  - Plasma lipid analysis
  - Oil Red-O lipid analysis of liver
  - Liver Glycogen measurement
  - Western blotting for protein abundance
  - Insulin tolerance test
  - Euglycemic hyperinsulinemic clamps
  - MBB derivatization of whole blood and plasma
  - Fluorometric quantification of MBB-sulfur species
  - Sulfur metabolite analysis from liver
  - P3 fluorescence detection of sulfide in hepatocytes
  - Quantification of hydrogen sulfide levels using MitoA *in vivo* exomarker
  - Preparation of hepatic mitochondria
  - Amperometric analysis of sulfide disposal
  - Mitochondrial ROS (MitoSOX) measurement in H_2O_2 treated hepatocytes
  - Persulfidation Mass Spec and GO term analysis

- ○ GO enrichment analysis
- ○ Focused analysis of persulfidation in gluconeogenesis proteins
- ○ Persulfidation labeling and western blotting from frozen liver
- ○ Mass spec analysis of liver protein
- ○ GO and KEGG enrichment analysis of proteome data
- ○ Transcription factor enrichment analysis
- ○ NRF2 target identification and proteome analysis
- ○ Electron micrograph imaging
- ○ Seahorse respiratory analysis
- ○ Mitochondrial stress test (MST)
- ○ Octanoate rescue test
- ○ Real time for mRNA analysis
- QUANTIFICATION AND STATISTICAL ANALYSIS

SUPPLEMENTAL INFORMATION

Supplemental information can be found online at https://doi.org/10.1016/j.celrep.2021.109958.

ACKNOWLEDGMENTS

This work was funded by a Wellcome Trust New Investigator Award (100981/Z/13/Z to N.M.M.) and a Diabetes UK grant (17/0005697). R.C.H. was funded by a Medical Research Council Discovery Award (MC-PC-15076). We would like to thank Prof. Ken Olsen and Eric DeLeon (Notre Dame) for advice regarding establishing sulfide measurement by probe.

AUTHOR CONTRIBUTIONS

N.M.M. and R.N.C. conceived the experiments. R.N.C., M.T.G.G., M.E.B.-L., M.L., V.V., B.E., T.L.B., M.B., H.S., S.G.D., N.Z.M.H., C.M., A.T., N.F., and T.G., performed the experiments. R.N.C., M.E.B.-L., P.L.F., V.V., T.L.B., N.Z.M.H., T.S., F.B., T.G., R.C.H., B.S., G.A.G., A.J.F., C.S., R.B., and N.M.M. analyzed and interpreted data and commented on the manuscript. K.H.A. and S.S. generated reagents. C.M. and R.C.H. generated reagents. R.N.C. and N.M.M. wrote the manuscript.

DECLARATION OF INTERESTS

The authors declare no competing interests.

Received: December 16, 2020
Revised: July 6, 2021
Accepted: October 15, 2021
Published: November 9, 2021

REFERENCES

Abe, K., and Kimura, H. (1996). The possible role of hydrogen sulfide as an endogenous neuromodulator. J. Neurosci. *16*, 1066–1071.

Arndt, S., Baeza-Garza, C.D., Logan, A., Rosa, T., Wedmann, R., Prime, T.A., Martin, J.L., Saeb-Parsy, K., Krieg, T., Filipovic, M.R., et al. (2017). Assessment of H_2S *in vivo* using the newly developed mitochondria-targeted mass spectrometry probe MitoA. J. Biol. Chem. *292*, 7761–7773.

Banerjee, R., Chiku, T., Kabil, O., Libiad, M., Motl, N., and Yadav, P.K. (2015). Assay methods for H2S biogenesis and catabolism enzymes. Methods Enzymol. *554*, 189–200.

Billaut-Laden, I., Allorge, D., Crunelle-Thibaut, A., Rat, E., Cauffiez, C., Chevalier, D., Houdret, N., Lo-Guidice, J.M., and Broly, F. (2006). Evidence for a functional genetic polymorphism of the human thiosulfate sulfurtransferase (Rhodanese), a cyanide and H2S detoxification enzyme. Toxicology *225*, 1–11.

Bonomi, F., Pagani, S., Cerletti, P., and Cannella, C. (1977). Rhodanese-Mediated sulfur transfer to succinate dehydrogenase. Eur. J. Biochem. *72*, 17–24.

Bromley, J., Hughes, B.G., Leong, D.C., and Buckley, N.A. (2005). Life-threatening interaction between complementary medicines: cyanide toxicity following ingestion of amygdalin and vitamin C. Ann. Pharmacother. *39*, 1566–1569.

Caron, S., Verrijken, A., Mertens, I., Samanez, C.H., Mautino, G., Haas, J.T., Duran-Sandoval, D., Prawitt, J., Francque, S., Vallez, E., et al. (2011). Transcriptional activation of apolipoprotein CIII expression by glucose may contribute to diabetic dyslipidemia. Arterioscler. Thromb. Vasc. Biol. *31*, 513–519.

Carter, R.N., and Morton, N.M. (2016). Cysteine and hydrogen sulphide in the regulation of metabolism: insights from genetics and pharmacology. J. Pathol. *238*, 321–332.

Chen, X., Jhee, K.H., and Kruger, W.D. (2004). Production of the neuromodulator H2S by cystathionine beta-synthase via the condensation of cysteine and homocysteine. J. Biol. Chem. *279*, 52082–52086.

Consoli, A., Nurjhan, N., Capani, F., and Gerich, J. (1989). Predominant role of gluconeogenesis in increased hepatic glucose production in NIDDM. Diabetes *38*, 550–557.

Cuadrado, A., Rojo, A.I., Wells, G., Hayes, J.D., Cousin, S.P., Rumsey, W.L., Attucks, O.C., Franklin, S., Levonen, A.L., Kensler, T.W., and Dinkova-Kostova, A.T. (2019). Therapeutic targeting of the NRF2 and KEAP1 partnership in chronic diseases. Nat. Rev. Drug Discov. *18*, 295–317.

Curry, S.C., Carlton, M.W., and Raschke, R.A. (1997). Prevention of fetal and maternal cyanide toxicity from nitroprusside with coinfusion of sodium thiosulfate in gravid ewes. Anesth. Analg. *84*, 1121–1126.

Desai, K.M., Chang, T., Untereiner, A., and Wu, L. (2011). Hydrogen sulfide and the metabolic syndrome. Expert Rev. Clin. Pharmacol. *4*, 63–73.

Distrutti, E., Mencarelli, A., Santucci, L., Renga, B., Orlandi, S., Donini, A., Shah, V., and Fiorucci, S. (2008). The methionine connection: homocysteine and hydrogen sulfide exert opposite effects on hepatic microcirculation in rats. Hepatology *47*, 659–667. https://doi.org/10.1002/hep.22037.

Feng, X., Chen, Y., Zhao, J., Tang, C., Jiang, Z., and Geng, B. (2009). Hydrogen sulfide from adipose tissue is a novel insulin resistance regulator. Biochem. Biophys. Res. Commun. *380*, 153–159. https://doi.org/10.1016/j.bbrc.2009.01.059.

Filipovic, M.R., Zivanovic, J., Alvarez, B., and Banerjee, R. (2017). Chemical Biology of H2S Signaling through Persulfidation. Chem. Rev. *118*, 1253–1337.

Fiorucci, S., Antonelli, E., Mencarelli, A., Orlandi, S., Renga, B., Rizzo, G., Distrutti, E., Shah, V., and Morelli, A. (2005). The third gas: H2S regulates perfusion pressure in both the isolated and perfused normal rat liver and in cirrhosis. Hepatology *42*, 539–548.

Gao, X.H., Krokowski, D., Guan, B.J., Bederman, I., Majumder, M., Parisien, M., Diatchenko, L., Kabil, O., Willard, B., Banerjee, R., et al. (2015). Quantitative H2S-mediated protein sulfhydration reveals metabolic reprogramming during the integrated stress response. eLife *4*, e10067.

Geng, B., Cai, B., Liao, F., Zheng, Y., Zeng, Q., Fan, X., Gong, Y., Yang, J., Cui, Q.H., Tang, C., and Xu, G.H. (2013). Increase or decrease hydrogen sulfide exert opposite lipolysis, but reduce global insulin resistance in high fatty diet induced obese mice. PLoS ONE *8*, e73892.

Gerő, D., Torregrossa, R., Perry, A., Waters, A., Le-Trionnaire, S., Whatmore, J.L., Wood, M., and Whiteman, M. (2016). The novel mitochondria-targeted hydrogen sulfide (H2S) donors AP123 and AP39 protect against hyperglycemic injury in microvascular endothelial cells in vitro. Pharmacol. Res. *113*, 186–198.

Ghosh Dastidar, S., Jagatheesan, G., Haberzettl, P., Shah, J., Hill, B.G., Bhatnagar, A., and Conklin, D.J. (2018). Glutathione S -transferase P deficiency induces glucose intolerance via JNK-dependent enhancement of hepatic gluconeogenesis. Am. J. Physiol. Endocrinol. Metab. *315*, E1005–E1018.

Hildebrandt, T.M., and Grieshaber, M.K. (2008). Three enzymatic activities catalyze the oxidation of sulfide to thiosulfate in mammalian and invertebrate mitochondria. FEBS J. *275*, 3352–3361.

CellPress
OPEN ACCESS

Hildebrandt, T.M., Di Meo, I., Zeviani, M., Viscomi, C., and Braun, H.P. (2013). Proteome adaptations in Ethe1-deficient mice indicate a role in lipid catabolism and cytoskeleton organization via post-translational protein modifications. Biosci. Rep. *33*, 575–584.

Hine, C., Harputlugil, E., Zhang, Y., Ruckenstuhl, C., Lee, B.C., Brace, L., Longchamp, A., Treviño-Villarreal, J.H., Mejia, P., Ozaki, C.K., et al. (2015). Endogenous hydrogen sulfide production is essential for dietary restriction benefits. Cell *160*, 132–144.

Hine, C., Kim, H.J., Zhu, Y., Harputlugil, E., Longchamp, A., Matos, M.S., Ramadoss, P., Bauerle, K., Brace, L., Asara, J.M., et al. (2017). Hypothalamic-Pituitary Axis Regulates Hydrogen Sulfide Production. Cell Metab. *25*, 1320–1333.e5.

Hine, C., Zhu, Y., Hollenberg, A.N., and Mitchell, J.R. (2018). Dietary and Endocrine Regulation of Endogenous Hydrogen Sulfide Production: Implications for Longevity. Antioxid. Redox Signal. *28*, 1483–1502.

Jackson, M.R., Melideo, S.L., and Jorns, M.S. (2012). Human sulfide:quinone oxidoreductase catalyzes the first step in hydrogen sulfide metabolism and produces a sulfane sulfur metabolite. Biochemistry *51*, 6804–6815.

Jain, S.K., Micinski, D., Huning, L., Kahlon, G., Bass, P.F., and Levine, S.N. (2014). Vitamin D and L-cysteine levels correlate positively with GSH and negatively with insulin resistance levels in the blood of type 2 diabetic patients. Eur. J. Clin. Nutr. *68*, 1148–1153.

Kabil, O., Motl, N., and Banerjee, R. (2014). H2S and its role in redox signaling. Biochim. Biophys. Acta *1844*, 1355–1366.

Karwi, Q.G., Bice, J.S., and Baxter, G.F. (2018). Pre- and postconditioning the heart with hydrogen sulfide (H_2S) against ischemia/reperfusion injury in vivo: a systematic review and meta-analysis. Basic Res. Cardiol. *113*, 6.

Kashala-Abotnes, E., Okitundu, D., Mumba, D., Boivin, M.J., Tylleskär, T., and Tshala-Katumbay, D. (2019). Konzo: a distinct neurological disease associated with food (cassava) cyanogenic poisoning. Brain Res. Bull. *145*, 87–91.

Kim, M.S., Krawczyk, S.A., Doridot, L., Fowler, A.J., Wang, J.X., Trauger, S.A., Noh, H.L., Kang, H.J., Meissen, J.K., Blatnik, M., et al. (2016). ChREBP regulates fructose-induced glucose production independently of insulin signaling. J. Clin. Invest. *126*, 4372–4386.

Kimura, Y., Koike, S., Shibuya, N., Lefer, D., Ogasawara, Y., and Kimura, H. (2017). 3-Mercaptopyruvate sulfurtransferase produces potential redox regulators cysteine- and glutathione-persulfide (Cys-SSH and GSSH) together with signaling molecules H_2S_2, H_2S_3 and H_2S. Sci. Rep. *7*, 10459.

Koike, S., Nishimoto, S., and Ogasawara, Y. (2017). Cysteine persulfides and polysulfides produced by exchange reactions with H_2S protect SH-SY5Y cells from methylglyoxal-induced toxicity through Nrf2 activation. Redox Biol. *12*, 530–539.

Krishnan, N., Fu, C., Pappin, D.J., and Tonks, N.K. (2011). H2S-Induced sulfhydration of the phosphatase PTP1B and its role in the endoplasmic reticulum stress response. Sci. Signal. *4*, ra86.

Lee, B.C., Kaya, A., and Gladyshev, V.N. (2016). Methionine restriction and lifespan control. Ann. N Y Acad. Sci. *1363*, 116–124.

Lewis, G.F., Carpentier, A., Adeli, K., and Giacca, A. (2002). Disordered fat storage and mobilization in the pathogenesis of insulin resistance and type 2 diabetes. Endocr. Rev. *23*, 201–229.

Libiad, M., Yadav, P.K., Vitvitsky, V., Martinov, M., and Banerjee, R. (2014). Organization of the human mitochondrial hydrogen sulfide oxidation pathway. J. Biol. Chem. *289*, 30901–30910.

Libiad, M., Sriraman, A., and Banerjee, R. (2015). Polymorphic variants of human rhodanese exhibit differences in thermal stability and sulfur transfer kinetics. J. Biol. Chem. *290*, 23579–23588.

Libiad, M., Motl, N., Akey, D.L., Sakamoto, N., Fearon, E.R., Smith, J.L., and Banerjee, R. (2018). Thiosulfate Sulfurtransferase like Domain Containing 1 Protein Interacts with Thioredoxin. J. Biol. Chem. *293*, 2675–2686.

Lutchmansingh, F.K., Hsu, J.W., Bennett, F.I., Badaloo, A.V., McFarlane-Anderson, N., Gordon-Strachan, G.M., Wright-Pascoe, R.A., Jahoor, F., and Boyne, M.S. (2018). Glutathione metabolism in type 2 diabetes and its relationship with microvascular complications and glycemia. PLoS ONE *13*, e0198626.

Mani, S., Li, H., Untereiner, A., Wu, L., Yang, G., Austin, R.C., Dickhout, J.G., Lhoták, Š., Meng, Q.H., and Wang, R. (2013). Decreased endogenous production of hydrogen sulfide accelerates atherosclerosis. Circulation *127*, 2523–2534.

Mani, S., Cao, W., Wu, L., and Wang, R. (2014). Hydrogen sulfide and the liver. Nitric Oxide *41*, 62–71.

Mason, T.M. (1998). The role of factors that regulate the synthesis and secretion of very-low-density lipoprotein by hepatocytes. Crit. Rev. Clin. Lab. Sci. *35*, 461–487.

Meakin, P.J., Chowdhry, S., Sharma, R.S., Ashford, F.B., Walsh, S.V., McCrimmon, R.J., Dinkova-Kostova, A.T., Dillon, J.F., Hayes, J.D., and Ashford, M.L. (2014). Susceptibility of Nrf2-Null Mice to Steatohepatitis and Cirrhosis upon Consumption of a High-Fat Diet Is Associated with Oxidative Stress, Perturbation of the Unfolded Protein Response, and Disturbance in the Expression of Metabolic Enzymes but Not with Insulin Resistance. Mol. Cell. Biol. *34*, 3305–3320.

Mikami, Y., Shibuya, N., Kimura, Y., Nagahara, N., Ogasawara, Y., and Kimura, H. (2011). Thioredoxin and dihydrolipoic acid are required for 3-mercaptopyruvate sulfurtransferase to produce hydrogen sulfide. Biochem. J. *439*, 479–485.

Miller, R.A., Buehner, G., Chang, Y., Harper, J.M., Sigler, R., and Smith-Wheelock, M. (2005). Methionine-deficient diet extends mouse lifespan, slows immune and lens aging, alters glucose, T4, IGF-I and insulin levels, and increases hepatocyte MIF levels and stress resistance. Aging Cell *4*, 119–125.

Mishanina, T.V., Libiad, M., and Banerjee, R. (2015). Biogenesis of reactive sulfur species for signaling by hydrogen sulfide oxidation pathways. Nat. Chem. Biol. *11*, 457–464.

Mithieux, G., Bady, I., Gautier, A., Croset, M., Rajas, F., and Zitoun, C. (2003). Induction of control genes in intestinal gluconeogenesis is sequential during fasting and maximal in diabetes. Am. J. Physiol. Endocrinol. Metab. *286*, E370–E375.

Módis, K., Coletta, C., Erdélyi, K., Papapetropoulos, A., and Szabo, C. (2013). Intramitochondrial hydrogen sulfide production by 3-mercaptopyruvate sulfurtransferase maintains mitochondrial electron flow and supports cellular bioenergetics. FASEB J. *27*, 601–611.

Moreno-Navarrete, J.M., Petrov, P., Serrano, M., Ortega, F., García-Ruiz, E., Oliver, P., Ribot, J., Ricart, W., Palou, A., Bonet, M.L., and Fernández-Real, J.M. (2013). Decreased RB1 mRNA, protein, and activity reflect obesity-induced altered adipogenic capacity in human adipose tissue. Diabetes *62*, 1923–1931.

Morris, A.A., Page, R.L., 2nd, Baumgartner, L.J., Mueller, S.W., MacLaren, R., Fish, D.N., and Kiser, T.H. (2017). Thiocyanate Accumulation in Critically Ill Patients Receiving Nitroprusside Infusions. J. Intensive Care Med. *32*, 547–553.

Morton, N.M., Nelson, Y.B., Michailidou, Z., Di Rollo, E.M., Ramage, L., Hadoke, P.W., Seckl, J.R., Bunger, L., Horvat, S., Kenyon, C.J., and Dunbar, D.R. (2011). A stratified transcriptomics analysis of polygenic fat and lean mouse adipose tissues identifies novel candidate obesity genes. PLoS ONE *6*, e23944.

Morton, N.M., Beltram, J., Carter, R.N., Michailidou, Z., Gorjanc, G., McFadden, C., Barrios-Llerena, M.E., Rodriguez-Cuenca, S., Gibbins, M.T., Aird, R.E., et al. (2016). Genetic identification of thiosulfate sulfurtransferase as an adipocyte-expressed antidiabetic target in mice selected for leanness. Nat. Med. *22*, 771–779.

Mosharov, E., Cranford, M.R., and Banerjee, R. (2000). The quantitatively important relationship between homocysteine metabolism and glutathione synthesis by the transsulfuration pathway and its regulation by redox changes. Biochemistry *39*, 13005–13011.

Mutel, E., Gautier-Stein, A., Abdul-Wahed, A., Amigó-Correig, M., Zitoun, C., Stefanutti, A., Houberdon, I., Tourette, J.A., Mithieux, G., and Rajas, F. (2011). Control of blood glucose in the absence of hepatic glucose production

during prolonged fasting in mice: induction of renal and intestinal gluconeogenesis by glucagon. Diabetes *60*, 3121–3131.

Nagahara, N. (2011). Catalytic site cysteines of thiol enzyme: sulfurtransferases. J. Amino Acids *2011*, 709404.

Nagahara, N. (2018). Multiple role of 3-mercaptopyruvate sulfurtransferase: antioxidative function, H2S and polysulfide production and possible SOx production. Br. J. Pharmacol. *175*, 577–589.

Nagahara, N., Tanaka, M., Tanaka, Y., and Ito, T. (2019). Novel Characterization of Antioxidant Enzyme, 3-Mercaptopyruvate Sulfurtransferase-Knockout Mice: Overexpression of the Evolutionarily-Related Enzyme Rhodanese. Antioxidants *8*, 116.

Nandi, D.L., and Westley, J. (1998). Reduced thioredoxin as a sulfur-acceptor substrate for rhodanese. Int. J. Biochem. Cell Biol. *30*, 973–977.

Nandi, D.L., Horowitz, P.M., and Westley, J. (2000). Rhodanese as a thioredoxin oxidase. Int. J. Biochem. Cell Biol. *32*, 465–473.

Norris, E.J., Culberson, C.R., Narasimhan, S., and Clemens, M.G. (2011). The liver as a central regulator of hydrogen sulfide. Shock *36*, 242–250.

O'Brien, B., Quigg, C., and Leong, T. (2005). Severe cyanide toxicity from 'vitamin supplements'. Eur. J. Emerg. Med. *12*, 257–258.

Onkenhout, W., Venizelos, V., van der Poel, P.F., van den Heuvel, M.P., and Poorthuis, B.J. (1995). Identification and Quantification of Intermediates of Unsaturated Fatty Acid Metabolism in Plasma of Patients with Fatty Acid Oxidation Disorders. Clin. Chem. *41*, 1467–1474.

Owen, O.E., Felig, P., Morgan, A.P., Wahren, J., and Cahill, G.F., Jr. (1969). Liver and kidney metabolism during prolonged starvation. J. Clin. Invest. *48*, 574–583.

Pagani, S., and Galante, Y.M. (1983). Interaction of rhodanese with mitochondrial NADH dehydrogenase. Biochim. Biophys. Acta *742*, 278–284.

Perez-Riverol, Y., Csordas, A., Bai, J., Bernal-Llinares, M., Hewapathirana, S., Kundu, D.J., Inuganti, A., Griss, J., Mayer, G., Eisenacher, M., et al. (2019). The PRIDE database and related tools and resources in 2019: improving support for quantification data. Nucleic Acids Res. *47* (*D1*), D442–D450.

Peters, J.M., Hennuyer, N., Staels, B., Fruchart, J.C., Fievet, C., Gonzalez, F.J., and Auwerx, J. (1997). Alterations in lipoprotein metabolism in peroxisome proliferator-activated receptor α-deficient mice. J. Biol. Chem. *272*, 27307–27312.

Pichette, J., and Gagnon, J. (2016). Implications of Hydrogen Sulfide in Glucose Regulation: How H2S Can Alter Glucose Homeostasis through Metabolic Hormones. Oxid. Med. Cell. Longev. *2016*, 3285074. https://doi.org/10.1155/2016/3285074.

Qian, K., Zhong, S., Xie, K., Yu, D., Yang, R., and Gong, D.W. (2015). Hepatic ALT isoenzymes are elevated in gluconeogenic conditions including diabetes and suppressed by insulin at the protein level. Diabetes Metab. Res. Rev. *31*, 562–571.

Reiffenstein, R. (1992). Toxicology Of Hydrogen Sulfide. Annu. Rev. Pharmacol. Toxicol. *32*, 109–134.

Rooney, J., Oshida, K., Vasani, N., Vallanat, B., Ryan, N., Chorley, B.N., Wang, X., Bell, D.A., Wu, K.C., Aleksunes, L.M., et al. (2018). Activation of Nrf2 in the liver is associated with stress resistance mediated by suppression of the growth hormone-regulated STAT5b transcription factor. PLoS ONE *13*, e0200004.

Rui, L. (2014). Energy metabolism in the liver. Compr. Physiol. *4*, 177–197.

Sasaki, M., Sasako, T., Kubota, N., Sakurai, Y., Takamoto, I., Kubota, T., Inagi, R., Seki, G., Goto, M., Ueki, K., et al. (2017). Dual regulation of gluconeogenesis by insulin and glucose in the proximal tubules of the kidney. Diabetes *66*, 2339–2350.

Sestito, S., Nesi, G., Pi, R., Macchia, M., and Rapposelli, S. (2017). Hydrogen Sulfide: A Worthwhile Tool in the Design of New Multitarget Drugs. Front Chem. *5*, 72.

Shibuya, N., Tanaka, M., Yoshida, M., Ogasawara, Y., Togawa, T., Ishii, K., and Kimura, H. (2009). 3-Mercaptopyruvate sulfurtransferase produces hydrogen sulfide and bound sulfane sulfur in the brain. Antioxid. Redox Signal. *11*, 703–714.

Shimokawa, I., Komatsu, T., Hayashi, N., Kim, S.E., Kawata, T., Park, S., Hayashi, H., Yamaza, H., Chiba, T., and Mori, R. (2015). The life-extending effect of dietary restriction requires Foxo3 in mice. Aging Cell *14*, 707–709, 10.

Simeonova, F.P., and Fishbein, L.; World Health Organization & International Programme on Chemical Safety (2004). Hydrogen cyanide and cyanides : human health aspects. https://apps.who.int/iris/handle/10665/42942.

Singh, S., Padovani, D., Leslie, R.A., Chiku, T., and Banerjee, R. (2009). Relative contributions of cystathionine beta-synthase and gamma-cystathionase to H2S biogenesis via alternative trans-sulfuration reactions. J. Biol. Chem. *284*, 22457–22466.

Singha, S., Kim, D., Moon, H., Wang, T., Kim, K.H., Shin, Y.H., Jung, J., Seo, E., Lee, S.J., and Ahn, K.H. (2015). Toward a selective, sensitive, fast-responsive, and biocompatible two-photon probe for hydrogen sulfide in live cells. Anal. Chem. *87*, 1188–1195.

Slocum, S.L., Skoko, J.J., Wakabayashi, N., Aja, S., Yamamoto, M., Kensler, T.W., and Chartoumpekis, D.V. (2016). Keap1/Nrf2 pathway activation leads to a repressed hepatic gluconeogenic and lipogenic program in mice on a high-fat diet. Arch. Biochem. Biophys. *591*, 57–65.

Smirnov, A., Comte, C., Mager-Heckel, A.M., Addis, V., Krasheninnikov, I.A., Martin, R.P., Entelis, N., and Tarassov, I. (2010). Mitochondrial enzyme rhodanese is essential for 5 S ribosomal RNA import into human mitochondria. J. Biol. Chem. *285*, 30792–30803.

Sookoian, S., Castaño, G.O., Scian, R., Fernández Gianotti, T., Dopazo, H., Rohr, C., Gaj, G., San Martino, J., Sevic, I., Flichman, D., and Pirola, C.J. (2016). Serum aminotransferases in nonalcoholic fatty liver disease are a signature of liver metabolic perturbations at the amino acid and Krebs cycle level. Am. J. Clin. Nutr. *103*, 422–434.

Steele, R., Wall, J.S., De Bodo, R.C., and Altszuler, N. (1956). Measurement of size and turnover rate of body glucose pool by the isotope dilution method. Am. J. Physiol. *187*, 15–24.

Stumvoll, M. (1998). Glucose production by the human kidney–its importance has been underestimated. Nephrol. Dial. Transplant. *13*, 2996–2999.

Swerdlow, D.I., Preiss, D., Kuchenbaecker, K.B., Holmes, M.V., Engmann, J.E., Shah, T., Sofat, R., Stender, S., Johnson, P.C., Scott, R.A., et al.; DIAGRAM Consortium; MAGIC Consortium; InterAct Consortium (2015). HMG-coenzyme A reductase inhibition, type 2 diabetes, and bodyweight: evidence from genetic analysis and randomised trials. Lancet *385*, 351–361.

Szabo, C. (2011). Roles of Hydrogen Sulfide in the Pathogenesis of Diabetes Mellitus and Its Complications. Antioxid. Redox Signal. *17*, 68–80.

Szabó, G., Veres, G., Radovits, T., Gero, D., Módis, K., Miesel-Gröschel, C., Horkay, F., Karck, M., and Szabó, C. (2011). Cardioprotective effects of hydrogen sulfide. Nitric Oxide *25*, 201–210.

Szabo, C., Ransy, C., Módis, K., Andriamihaja, M., Murghes, B., Coletta, C., Olah, G., Yanagi, K., and Bouillaud, F. (2014). Regulation of mitochondrial bioenergetic function by hydrogen sulfide. Part I. Biochemical and physiological mechanisms. Br. J. Pharmacol. *171*, 2099–2122.

Tiranti, V., Viscomi, C., Hildebrandt, T., Di Meo, I., Mineri, R., Tiveron, C., Levitt, M.D., Prelle, A., Fagiolari, G., Rimoldi, M., and Zeviani, M. (2009). Loss of ETHE1, a mitochondrial dioxygenase, causes fatal sulfide toxicity in ethylmalonic encephalopathy. Nat. Med. *15*, 200–205.

Tolwani, R.J., Hamm, D.A., Tian, L., Sharer, J.D., Vockley, J., Rinaldo, P., Matern, D., Schoeb, T.R., and Wood, P.A. (2005). Medium-chain acyl-CoA dehydrogenase deficiency in gene-targeted mice. PLoS Genet. *1*, 205–212.

Tonelli, C., Chio, I.I.C., and Tuveson, D.A. (2018). Transcriptional Regulation by Nrf2. Antioxid. Redox Signal. *29*, 1727–1745.

Uyeda, K., and Repa, J.J. (2006). Carbohydrate response element binding protein, ChREBP, a transcription factor coupling hepatic glucose utilization and lipid synthesis. Cell Metab. *4*, 107–110.

Vinnakota, C.V., Peetha, N.S., Perrizo, M.G., Ferris, D.G., Oda, R.P., Rockwood, G.A., and Logue, B.A. (2012). Comparison of cyanide exposure markers in the biofluids of smokers and non-smokers. Biomarkers *17*, 625–633.

CellPress
OPEN ACCESS

Vitvitsky, V., Thomas, M., Ghorpade, A., Gendelman, H.E., and Banerjee, R. (2006). A functional transsulfuration pathway in the brain links to glutathione homeostasis. J. Biol. Chem. *281*, 35785–35793.

Vitvitsky, V., Yadav, P.K., Kurthen, A., and Banerjee, R. (2015). Sulfide oxidation by a noncanonical pathway in red blood cells generates thiosulfate and polysulfides. J. Biol. Chem. *290*, 8310–8320.

Vitvitsky, V., Yadav, P.K., An, S., Seravalli, J., Cho, U.S., and Banerjee, R. (2017). Structural and mechanistic insights into hemoglobincatalyzed hydrogen sulfide oxidation and the fate of polysulfide products. J. Biol. Chem. *292*, 5584–5592.

Walsh, J., Jenkins, R.E., Wong, M., Olayanju, A., Powell, H., Copple, I., O'Neill, P.M., Goldring, C.E., Kitteringham, N.R., and Park, B.K. (2014). Identification and quantification of the basal and inducible Nrf2-dependent proteomes in mouse liver: biochemical, pharmacological and toxicological implications. J. Proteomics *108*, 171–187.

Wang, R. (2012). Physiological implications of hydrogen sulfide: a whiff exploration that blossomed. Physiol. Rev. *92*, 791–896.

Wedmann, R., Onderka, C., Wei, S., Szijártó, I.A., Miljkovic, J.L., Mitrovic, A., Lange, M., Savitsky, S., Yadav, P.K., Torregrossa, R., et al. (2016). Improved tag-switch method reveals that thioredoxin acts as depersulfidase and controls the intracellular levels of protein persulfidation. Chem. Sci. (Camb.) *7*, 3414–3426.

Whiteman, M., Le Trionnaire, S., Chopra, M., Fox, B., and Whatmore, J. (2011). Emerging role of hydrogen sulfide in health and disease: critical appraisal of biomarkers and pharmacological tools. Clin. Sci. (Lond.) *121*, 459–488.

World Health Organization (2016). Global Report on Diabetes. https://www.who.int/publications/i/item/9789241565257.

Wu, D., Zheng, N., Qi, K., Cheng, H., Sun, Z., Gao, B., Zhang, Y., Pang, W., Huangfu, C., Ji, S., et al. (2015). Exogenous hydrogen sulfide mitigates the fatty liver in obese mice through improving lipid metabolism and antioxidant potential. Med. Gas Res. *5*, 1.

Xie, L., Gu, Y., Wen, M., Zhao, S., Wang, W., Ma, Y., Meng, G., Han, Y., Wang, Y., Liu, G., et al. (2016). Hydrogen sulfide induces Keap1 S-sulfhydration and suppresses diabetes-accelerated atherosclerosis via Nrf2 activation. Diabetes *65*, 3171–3184.

Xue, R., Hao, D.D., Sun, J.P., Li, W.W., Zhao, M.M., Li, X.H., Chen, Y., Zhu, J.H., Ding, Y.J., Liu, J., and Zhu, Y.C. (2013). Hydrogen sulfide treatment promotes glucose uptake by increasing insulin receptor sensitivity and ameliorates kidney lesions in type 2 diabetes. Antioxid. Redox Signal. *19*, 5–23.

Yadav, P.K., Yamada, K., Chiku, T., Koutmos, M., and Banerjee, R. (2013). Structure and kinetic analysis of H2S production by human mercaptopyruvate sulfurtransferase. J. Biol. Chem. *288*, 20002–20013.

Yang, G., Zhao, K., Ju, Y., Mani, S., Cao, Q., Puukila, S., Khaper, N., Wu, L., and Wang, R. (2013). Hydrogen sulfide protects against cellular senescence via S-sulfhydration of Keap1 and activation of Nrf2. Antioxid. Redox Signal. *18*, 1906–1919.

Zhang, Y.K.J., Wu, K.C., Liu, J., and Klaassen, C.D. (2012). Nrf2 deficiency improves glucose tolerance in mice fed a high-fat diet. Toxicol. Appl. Pharmacol. *264*, 305–314.

Zhang, L., Yang, G., Untereiner, A., Ju, Y., Wu, L., and Wang, R. (2013). Hydrogen sulfide impairs glucose utilization and increases gluconeogenesis in hepatocytes. Endocrinology *154*, 114–126.

Zhang, L., Wang, Y., Li, Y., Li, L., Xu, S., Feng, X., and Liu, S. (2018). Hydrogen sulfide (H2S)-releasing compounds: Therapeutic potential in cardiovascular diseases. Front. Pharmacol. *9*, 1066.

STAR★METHODS

KEY RESOURCES TABLE

REAGENT or RESOURCE	SOURCE	IDENTIFIER
Antibodies		
Anti-TST	GeneTex	GTX114858; RRID:AB_10620797
Anti-MPST	Abcam	ab224043
Anti-GOT1	Abcam	ab170950; RRID:AB_170950
Anti-GSTT1	Proteintech	15838-1-AP; RRID:AB_2116344
Anti-MAT1A	Abcam	ab129176; RRID:AB_11145300
Anti-BHMT	Proteintech	15965-1-AP; RRID:AB_2290472
Anti-CSAD	Abcam	ab91016; RRID:AB_10713222
Anti-PPCS	Atlas Antibodies	HPA031361; RRID:AB_10602150
IRDye 800CW Goat anti-Rabbit	Li-Cor	926-32211; RRID:AB_621843
IRDye 680RD Donkey anti-Mouse	Li-Cor	926-68072; RRID:AB_10953628
Anti-B-Actin	Abcam	ab8226; RRID:AB_306371
Anti-CoxIV	Abcam	Ab16056; RRID:AB_443304
Chemicals, peptides, and recombinant proteins		
sodium pyruvate $^{13}C_3$	Sigma-Aldrich	490717
Amyloglucosidase	Roche	ROAMYGLL
Antimycin A	Sigma-Aldrich	A8674
B-glycerophosphate	Sigma-Aldrich	G9422
B-mercaptoethanol	Sigma-Aldrich	444203
Bovine serum albumin, essentially fatty acid free	Sigma-Aldrich	10775835001
Carbonyl cyanide-p-trifluoromethoxyphenylhydrazone (FCCP)	Cayman Chemicals	15218
Oligomycin A	Cayman Chemicals	11342
DL-Carnitine hydrochloride	Sigma-Aldrich	C9500
Collagenase type 1	Worthington Laboratories	LS004194
cOmplete, Mini, EDTA-free Protease Inhibitor Cocktail	Roche	11836153001
Glucose, D-[3-^{3}H]	PerkinElmer	NET331C
Deoxycholic acid	Sigma-Aldrich	D2510
2′-deoxyguanosine 5′-diphosphate, sodium salt	Sigma-Aldrich	D9250
Dithiothreitol	Sigma-Aldrich	43816
Durcupan ACM	Sigma-Aldrich	44610
(+)- Etomoxir sodium salt hydrate	Sigma-Aldrich	E1905
EZ-link Maleimide-PEG2-Biotin	Thermo Fisher Scientific	21901BID
Fetal Calf Serum, Brazilian Origin	SLS Life Science	HYC85
Prestained Protein Marker	Proteintech	PL00001
Glutamax	Thermo Fisher Scientific	35050061
L-Glutamine	Sigma-Aldrich	G7513
Glycine	Sigma-Aldrich	50046
Hematoxylin solution	Abcam	Ab220365
Iodoacetamide	Sigma-Aldrich	I1149
L-cysteine	Sigma-Aldrich	30089
Lead citrate, tribasic trihydrate	Sigma-Aldrich	15326
Malate dehydrogenase, porcine heart	Sigma-Aldrich	442610-M

(Continued on next page)

Continued		
REAGENT or RESOURCE	SOURCE	IDENTIFIER
Skim Milk Powder	Millipore	70166
S-Methyl methanethiosulfonate	Sigma-Aldrich	64306
MitoSOX Red Mitochondrial superoxide indicator	Thermo Fisher Scientific	M36008
NADH, Grade I disodium salt	Roche	10107735001
Pierce NEM (N-ethylmaleimide)	Thermo Fisher Scientific	23030
Sodium octanoate	Sigma-Aldrich	C5038
Oil Red O	Sigma-Aldrich	O0625
Oligomycin A	Sigma-Aldrich	75351
Penicillin Streptomycin	Thermo Fisher Scientific	15140122
PERCOLL 8.5-9.5	Sigma-Aldrich	P1644
Phosphoenol pyruvate	Roche	10108294001
cOmplete protease cocktail inhibitor	Roche	04693159001
Rat tail collagen 1	Sigma-Aldrich	08-115
REVERT total protein stain	LICOR	926-11011
Rotenone	Sigma-Aldrich	R8875
Sequencing grade modified Trypsin	Promega	V5111
Sodium fluoride	Sigma-Aldrich	S7920
Sodium L-lactate	Sigma-Aldrich	71718
Sodium orthovanadate	Sigma-Aldrich	450243
Sodium pyrophosphate	Sigma-Aldrich	221368
Sodium pyruvate	Sigma-Aldrich	P8574
Sodium sulfate	Sigma-Aldrich	S9627
Sodium thiosulfate	Sigma-Aldrich	563188
Sodium sulfide	Sigma-Aldrich	407410
Sulforhodamine B dye	Sigma-Aldrich	230162
Taurine	Sigma-Aldrich	86329
Tetrabutylammonium phosphate	Sigma-Aldrich	86833
Trichloroacetic acid	Sigma-Aldrich	T6399
Triethylammonium bicarbonate	Sigma-Aldrich	18597
Trifluoracetic acid	Sigma-Aldrich	80457
Uranyl acetate	Electron Microscopy Sciences	22400
Urea	Sigma-Aldrich	U5128
XF Seahorse Base Media (DMEM)	Agilent	102353-100
^{14}C-2-deoxyglucose	Perkin Elmer	NEC495A
4-(2-Hydroxyethyl)-1-piperazine propanesulfonic acid	Sigma-Aldrich	1.15230
2,4-nitrofluorobenzene	Sigma-Aldrich	D1529
Critical commercial assays		
Infinity Triglyceride Assay	Thermo Fisher Scientific	TR22421
Infinity Cholesterol Assay	Thermo Fisher Scientific	TR13421
Glucose Hexokinase Assay	Abcam	Ab136957
iTRAQ reagent – 8PLEX	Sigma-Aldrich	4281663
Deposited data		
Proteome	ProteomeXchange	PXD028909
Persulfidome	ProteomeXchange	PXD028909
Experimental models: Cell lines		
Primary hepatocytes	C57BL/6J and *Tst*$^{-/-}$ mice	n/a

(*Continued on next page*)

Continued		
REAGENT or RESOURCE	SOURCE	IDENTIFIER
Experimental models: Organisms/strains		
C57BL/6J (JAX mice strain)	Charles River	Strain code: 632
Tst$^{-/-}$ C57BL/6N mouse (backcrossed for > 10 generations at University of Edinburgh)	University California (Davis) International Mouse Knockout Project	VG13928; model Tst$^{tm1(KOMP)Vlcg}$
Oligonucleotides		
Tst (mouse) FAM gene expression assay 4331182	Thermo Fisher	Mm00726109_m1
Mpst (mouse) FAM gene expression assay 4331182	Thermo Fisher	Mm00460389_m1
Gapdh (mouse) FAM gene expression assay 4331182	Thermo Fisher	Mm99999915_g1
Tbp (mouse) FAM gene expression assay 4331182	Thermo Fisher	Mm0000446973_m1
Software and algorithms		
Microsoft office		N/A
Graph Pad Prism v8, 9 and 10		N/A
Other		
Amersham Hybond P blotting membranes, PVDF	Merck	GE10600021
Microvette CB300 K2E EDTA tubes	Sarstedt	16.444.100
Ultra-0.5 centrifugal filter, 10K cut off	Millipore	UFC501096
Streptavidin-agarose beads	Thermo Scientific	20347
Formvar coated grids	Agar Scientific	AGS138
Standard rodent diet	SDS	RM1
Cornstarch diet	Research Diets	D12383
58% High fat diet	Research Diets	D12331

RESOURCE AVAILABILITY

Lead contact

Further information and requests for resources and reagents should be directed to and will be fulfilled by the lead contact, Nicholas M. Morton (nik.morton@ed.ac.uk).

Materials availability

No other new unique reagents were generated for the production of the data in this paper.

Data and code availability

- Proteomics and persulfidomics root data from the iTraq and persulfidated peptide mass spectrometry experiments have been deposited to the ProteomeXchange Consortium via the PRIDE (Perez-Riverol et al., 2019) partner repository with the dataset identifier ProteomeXchange: PXD028909.
- This paper does not report original code.
- Any additional information required to reanalyse the data reported in this paper is available from the lead contact upon request.

EXPERIMENTAL MODEL AND SUBJECT DETAILS

Experimental animals

All experiments were performed according to guidelines set out by the ethical committees of The University of Edinburgh and Physiogenex S.A.S, Prologue Biotech, Labége, FRANCE. Experiments were carried out within the framework of the Animals (Scientific Procedures) Act (1986) of the United Kingdom Home Office or related laws from the European Union (France). In all studies, animals within genotype cohorts were randomly assigned to diet or intervention groups. All animals were maintained in standard housing with 12 hour light and 12 hour dark cycles (7 a.m. to 7 p.m.) and *ad libitum* access to the appropriate diet. For *in vivo* experiments (pyruvate tolerance

test, insulin tolerance test, euglycaemic clamps), operators and animal handlers were blinded to the data, which was generated by a second individual who was blinded to the treatment regimen until the code was broken. All of the studies used male mice housed in cages of 3-6 individual littermates until intervention. The mice for this study originated from C57BL/6N *Tst*$^{-/-}$ mice (Morton et al., 2016) backcrossed onto the C57BL/6J genetic background for > 10 generations. Mice were placed onto high fat diet D12331, (58% calories from fat, Research Diets, New Brunswick, USA) from between 6-8 weeks of age, for 6-7 weeks prior to testing, and compared to mice maintained on standard low fat diets, RM1 or D12383 (low-fat high-cornstarch, Research Diets, New Brunswick, USA).

Hepatocyte preparations

Mice were killed by CO_2 asphyxiation, followed by cervical dislocation. The chest cavity was opened, the portal vein was cut and the thoracic vena cava was cannulated via the right atrium. The liver was perfused with (37°C) perfusion media (140 mM NaCl, 2.6 mM KCl, 0.28 mM Na_2HPO_4, 5 mM glucose, 10 mM HEPES, 0.5 mM EGTA, pH 7.4), 6 mls/min for 10 min. The liver was then perfused with digestion media (perfusion media, without EGTA, including 5 mM $CaCl_2$, and 100 U/ml collagenase type 1) for 5-7 min. Finally, the liver was perfused with perfusion media for a further 10 min. Cells were extruded from liver into DMEM medium (DMEM, 5.5 mM glucose, 10% FCS, 7 mM glutamine, and penicillin/streptomycin antibiotics), and then passed through a 40 micron filter. Cells were spun twice and washed with medium, at 500 rpm (47 g) for 5 min. Cells were spun through a 50% Percoll pH 8.5-9.5/DMEM solution at 1000 rpm (190 g) for 15 min to remove dead cells and non hepatocytic liver cell types. Hepatocytes collected in the pellet fractions were resuspended in medium and spun twice with washing at 500 rpm (47 g) 5 minutes. Yields and viability were assessed by counting using a haemocytometer, and proportion of trypan blue exclusion respectively. Yields ranged from between $2 \times 10^6 - 1.5 \times 10^7$ viable cells, and viability was above 85%. Unless otherwise stated, hepatocytes were seeded onto collagen coated tissue culture plastic (collagen from rat tails, Sigma), and maintained in DMEM with 5.5 mM glucose, 10% FCS, 7 mM glutamine, and antibiotics).

METHOD DETAILS

Pyruvate tolerance test

Blood glucose was measured from 16 hour fasted mice before (0 time) and following bolus sodium pyruvate administration (i.p. 1.5 mg/g bodyweight). Blood was collected following tail venesection at 0, 15, 30, 60 and 120 minutes after injection. Glucose was measured from blood using a Glucometer (*OneTouch*, Lifescan, Milpitas, USA or *Accu-Chek*, Performa nano, Roche).

PEPCK activity assay

Activity of phosphoenolpyruvate carboxykinase was measured from cytosol samples obtained from frozen liver. Samples were homogenized in 250 mM sucrose, 5 mM HEPES, pH 7.4. and centrifuged at 4°C, 12,000 rpm (17,390 g) for 15 min. Supernatants were ultracentrifuged at 4°C, 60,000 rpm (289,000 g) for 30 min. Activity of PEPCK from cytosolic fractions was inferred in this assay from NADH extinction, linked to the conversion of phosphoenol pyruvate into oxaloacetate in the presence of carbonate, dGDP and $MnCl_2$, and the subsequent conversion of oxaloacetate into malate by adding malate dehydrogenase. Baseline measurements at 340 nM (NADH) were taken for 20 min before adding phosphoenol pyruvate, and the reaction proper was initiated with dGDP. The reaction was then measured for a further 40 min.

^{13}C Pyruvate metabolite tracing

After overnight culture on collagen coated 6-well tissue culture plates, hepatocytes were incubated with 1 mM $^{13}C_3$ labeled pyruvate in serum free DMEM for 60 min. Metabolites were extracted by washing individual wells with ice-cold PBS and addition of cold extraction buffer (50% methanol, 30% acetonitrile, 20% water solution at −20°C or lower). Extracts were clarified and stored at −80°C until required. LC-MS was carried out using a 100 mm x 4.6 mm ZIC-pHILIC column (Merck-Millipore) using a Thermo Ultimate 3000 HPLC inline with a Q Exactive mass spectrometer. A 32 min gradient was developed over the column from 10% buffer A (20 mM ammonium carbonate), 90% buffer B (acetonitrile) to 95% buffer A, 5% buffer B. 10 μL of metabolite extract was applied to the column equilibrated in 5% buffer A, 95% buffer B. Q Exactive data were acquired with polarity switching and standard ESI source and spectrometer settings were applied (typical scan range 75-1050). Metabolites were identified based upon m/z values and retention time matching to standards.

Plasma lipid analysis

Mice were fasted with free access to water for 4 hours prior to cull by decapitation or pentobarbital euthanasia. Trunk blood (decapitation) was collected directly into Sarstedt Microvette CB 300 K2E EGTA containing plasma sample tubes (Sarstedt, Nümbrecht, Germany). Venous blood from the abdominal vena cava (post euthanisation) was collected into a BD Plastipak 1 mL syringe (BD, Madrid, Spain). This was then transferred to Sarstedt EGTA containing sample tubes for centrifugation. Blood samples obtained by either method were centrifuged at 20°C and 5000 rpm (2655 g) for 5 min to obtain plasma samples. Plasma samples were analyzed for cholesterol and triglyceride content by as previously described (Peters et al., 1997). Briefly, samples were subjected to gel filtration chromatography using an integrated Alliance HPLC separations module (e2695, Waters, Milford, US) to separate lipoproteins based on size. Effluent was immediately and continuously mixed with either triglyceride (Infinity Triglyceride, Thermo Scientific, Loughborough, UK) or cholesterol (Infinity Cholesterol, Thermo Scientific, Loughborough, UK) enzymatic colormetric detection

kits at the correct conditions for reaction (as specified in manufacturer's guidance). The optical density was then recorded using a spectrophotometer at the appropriate wavelength and the signal turned into a continuous trace i.e., a lipid profile. By identification of the lipoprotein peaks (based on their time of emergence from the chromatograph) the concentration for each could be calculated.

Oil Red-O lipid analysis of liver

5 μm cryostat cut frozen sections of liver were collected onto Superfrost slides (Thermo), and rinsed with 60% isopropanol. Slides were incubated in freshly prepared staining solution (2.1 mg/ml Oil Red O in 40% isopropanol/water) for 10 – 30 min and rinsed with 60% isopropanol. Slides for representative images were counterstained for nuclei in hematoxylin (Harris) for 1 minute. For image analysis, slides were not counterstained. All slides were then rinsed in running tap water for 2 min, before mounting. Sections were captured using an AxioScan Z1 slide scanner at × 40 magnification and analysis of the proportional area of Oil Red O staining (area of stain/ unit area of section) was performed using ImageJ software (National Institutes of Health), assessed by a blinded assessor.

Liver Glycogen measurement

Frozen liver samples (between 30-90 mg) were heated to 100°C in an Eppendorf tube with 0.3 mls of 30% KOH for 30 min with vigorous shaking at 10-min intervals. Samples were heated for a further 2-3 min after addition of 0.1 mL 1M Na_2SO_4 and 0.8 mL ethanol. Samples were then centrifuged at 4°C at 1011 g for 5 minutes. The supernatant was removed, and the pellet resuspended in distilled H_2O before 0.1 mL 1M Na_2SO_4 and 0.8 mL ethanol were again added, and samples boiled at 100°C for 5 min before centrifugation. This was repeated a final time to wash the sample. The pellet was resuspended in a 10 mg/ml (~1200 U/ml) amyloglucosidase enzyme in 0.3 M sodium acetate (pH 4.8). Samples were then incubated at 50°C for 2 hours. Quantification of samples was then performed using a standard hexokinase based glucose assay (Glucose (HK) Assay Kit, Sigma, GAHK20). The assay was performed following manufacturer's instructions and values calculated by extrapolation from a standard curve after measuring absorbance using a plate spectrophotometer (Molecular Devices OPTImax microplate reader and software, Molecular Devices, Wokingham, UK).

Western blotting for protein abundance

Frozen liver samples (stored −80°C) from mice were homogenized in protein lysis buffer (50 mM Tris, 270 mM sucrose, 50 mM NaF, 1 mM EDTA, 1 mM EGTA, 1% Triton X-100, 10 mM B-glycerophosphate, 5 mM Na Pyrophosphate, 1 mM orthovanadate, 0.1% β-Mercaptoethanol, 1 tablet protease inhibitor cocktail inhibitor, pH 7.4, all Sigma Aldritch). Samples were then centrifuged at 13,200 rpm (18500 g) for 15 min at 4°C and the supernatants aliquoted and stored at −80°C. Protein samples were loaded onto 10% acrylamide/bis-acrylamide gels (30% acrylamide, Sigma Aldritch) and separated by electrophoresis. A colored molecular weight marker was also run on all gels (Full range rainbow molecular weight markers, GE Healthcare). Gels were transferred overnight using a Bio Rad wet transfer system onto Amersham Hybond – P membranes (GE Healthcare). After transfer, for normalization of specific targets to total protein, membranes were stained using the REVERT total protein stain (LICOR), according to manufacturers' instruction. Following stain and wash, lanes of each sample were analyzed using a LI-COR Odyssey scanner (700nm channel). For blots using a house keeping protein for normalization, the total protein stain was not performed, and membranes were transferred directly to blocking. All membranes were blocked in Tris buffered saline with 0.01% tween (TBST, containing 5% skimmed milk powder (Marvel skimmed milk powder) for 1 hour and then rinsed in TBST. Blocked membranes were then incubated with the appropriate primary antibody in TBST containing 5% BSA (Sigma Aldritch) overnight at 4°C. Following three 5 min washes with TBST, secondary antibody incubation for all blots was with an appropriate green or red fluorescent antibody, incubated at room temperature for 2 hours in TBST containing 5% BSA. Membranes were washed three times in TBST then scanned using the LI-COR Odyssey scanner. Odyssey software (LI-COR Biosciences) was used to quantify band intensity. For normalization to a house keeping protein, the individual band intensity of B-actin was used for each sample. Primary antibodies used were; TST, Rabbit, GeneTex, GTX114858, MPST, Rabbit, Abcam, Ab224043, GOT1, Rabbit, Abcam, ab170950, GSTT1, Rabbit, Proteintech, 15838-1-AP, MAT1A, Rabbit, Abcam, ab129176, BHMT Rabbit, Proteintech, 15965-1-AP, CSAD, Rabbit, Abcam, ab91016, PPCS Rabbit, Atlas Antibodies, HPA031361. Secondary antibodies used were; IRDye800CW Goat anti-Rabbit, Li-Cor, 926-32211, IRDye 680RD Donkey anti-Mouse, Li-Cor, 926-68072. For normalization B-Actin, Mouse, Abcam, ab8226 was used for whole tissue, and Cox IV (mitochondrial loading control), Abcam, ab16056 was used for mitochondrial fractions.

Insulin tolerance test

Male C57BL/6J or $Tst^{-/-}$ mice were maintained on standard chow (RM1). Mice were fasted for 4 hours prior to injection i.p. of insulin (1 mU/g bodyweight, NovoRapid 100U/ml, Novo Nordisk). Tail venesection blood samples were taken prior to, and 15, 30, 60 and 120 minutes post injection. Blood glucose was measured from samples using a Glucometer (Accu-Check, Performa Nano, Roche). Blood glucose was plotted across time to evaluate net glucose accumulation in blood.

Euglycemic hyperinsulinemic clamps

Male C57BL/6J or $Tst^{-/-}$ mice were maintained on standard diet (RM1 (E) 801492, SDS) or high fat diet for 6 weeks (58% fat, D12331, Research Diets). Prior to performing the hyperinsulinemic euglycemic clamp an indwelling catheter was placed into the femoral vein under isoflurane anesthesia, sealed under the back skin, and glued onto the top of the skull. Clamps were performed 5-6 days after recovery from catheterization. Mice were fasted 6 hours prior to a basal blood sample was taken for glucose and insulin. Mice then

received a bolus of D-[3-3H] glucose (30 μCi) and perfused with 3H-glucose (30 μCi/kg/min at 2 μl/min) for 210 min (which covers the basal phase and hyperinsulinemic clamp). At steady state (60 min after start of perfusion), 5 μl of blood was collected and glycemia measured from tail tip every 10 min over 30 min for ^{3}H-radioactivity analysis for determination of whole body glucose turnover glycolysis and glycogen synthesis rate in the basal state. 90 min after start of perfusion, the hyperinsulinemic clamp starts by co-perfusion with insulin 8 mU/kg/min for the clamped phase over 120 min. Blood glucose was assessed every 10 minutes, and glucose infusion adjusted until steady state blood glucose (120 mg/dl ± 10 mg/dl) was achieved. 5 μl of blood was collected from, tail tip every 10 min for ^{3}H- radioactivity analysis. At 150 min after the start of perfusion, a bolus of ^{14}C-2-deoxyglucose (25 μCi) was perfused to evaluate tissue specific uptake. At the end of the perfusion (210 min), blood is collected from the retro-orbital sinus to measure plasma insulin and mice sacrificed by i.v. injection of pentobarbital and cervical dislocation. Tissues (Inguinal WAT, Epididymal WAT, Soleus muscle, Extensor digitorum longus muscle, Vastus lateralis muscle, Tibialis anterior muscle, Heart apex, Liver) were removed by dissection and flash frozen in liquid nitrogen (stored −80°C until measured). Tracers were used to calculate various aspects of glucose metabolism (Steele et al., 1956; Carter and Morton, 2016). Parameters measured or calculated include body weight, glucose infusion rate, whole body turnover, hepatic glucose production, whole body glycolytic rate, whole body glycogen synthesis rate, and tissue glucose utilization.

MBB derivatization of whole blood and plasma

Whole blood was taken after cull of mice, from trunk (following decapitation), or portal vein (following CO_2 euthanasia). EDTA-plasma was obtained from trunk blood following decapitation and collected onto ice. Blood for plasma was centrifuged within 15 min of collection for 5 min at 5000 rpm (2655 g) at 4°C. Blood and plasma samples (15-50 μl) were derivatized with monobromobimane by addition of 200 μL of 80 mM EPPS (4-(2-Hydroxyethyl)-1-piperazine propanesulfonic acid, 8 mM DTPA (diethylenetriaminepentaacetic acid) pH 8.0, 50% acetonitrile, 2.3 mM monobromobimane. Reaction vials were capped tightly and vortexed for 1 minute and incubated protected from light at room temperature for 30 min. 1 mL ethyl acetate was added, the tube capped and vortexed for 1 min and incubated protected from light for 10 min. The reaction vials were centrifuged at 1800 rpm (350 g) for 7 min to separate aqueous and organic layers. The organic layer was collected from each extraction, transferred to a 1.5 mL brown glass vial and the solvent was evaporated completely under a nitrogen stream. Acetonitrile (200 μL) was added to each vial, and the solvent was again evaporated to remove any traces of ethyl acetate. Dried MBB-derivatives were stored at −20°C until analyzed.

Fluorometric quantification of MBB-sulfur species

MBB-sulfur species (sulfide, thiosulfate, reduced glutathione, and cysteine) in samples was quantified by HPLC separation and detection with a fluorescence detector. The dried MBB derivatives were re-suspended in 50 μL of Buffer A (10 mM tetrabutylammonium phosphate aqueous, 10% methanol, 45 mM acetic acid adjusted to pH 3.4). The entire sample was transferred to an HPLC autosampler vial with a 200 μL glass sample insert, and the vial was closed with a penetrable cap. 20 μL of the sample was injected onto a C8 reverse-phase column (LiChrospher 60 RP-select B 5 μm 4.0 × 125 mm LiChroCART 125-4, Merck KGaA) and a guard column (LiChroCART 10-2, Superspher 60 RP-select B cartridge) on an Ultimate 3000 UHPLC+ focused system (Thermo Scientific). MBB derivatives were eluted with a linear gradient from 10% buffer B (10 mM tetrabutylammonium phosphate in methanol, 10% water, 45 mM acetic acid) to 100% buffer B over 30 min. The eluent was analyzed by fluorescence (λex = 380 nm, λem = 480 nm).

Sulfur metabolite analysis from liver

Livers from mice were removed promptly following decapitation (within 2 min), and frozen on powdered dry ice. Frozen tissue was pulverized and derivatized with either 2,4-dinitrofluorobenzene for detecting GSH or monobromobimane for detecting sulfide and thiosulfate as described previously (Mosharov et al., 2000; Vitvitsky et al., 2006, 2015).

P3 fluorescence detection of sulfide in hepatocytes

Hepatocytes were seeded in glass bottomed, collagen coated wells (0.75 cm^2, 12,500 hepatocytes per well) and cultured in DMEM with 5.5 mM glucose, 10% FCS, 4 mM glutamax or 7 mM glutamine, and antibiotics overnight. P3 H_2S reactive probe (Singha et al., 2015) was added to wells at 10 μM in serum free DMEM for 30 min, prior to gentle washing with Krebs phosphate buffered saline (pH 7.4). Plates were measured using the TECAN fluorescence plate reader, following excitation at 375 nm and detection at 510 nm. No-cell control wells were used for subtracting from the cell containing values. Corrected fluorescence emission data was normalized to protein as estimated by sulforhodamine dye. Briefly, after the run cells were fixed with 10% trichloroacetic acid overnight at 4°C. Cells were washed 9 times with tap water, and air-dried. Cells were incubated with 200 μL of 0.4% Sulforhodamine dye/1% acetic acid for 1 hour at room temperature. Stain was removed, and washed 4 times with 1% acetic acid, and then air-dried. Stain was dissolved in 200 μL of 10 mM Tris pH 10.5 for 30 min, and 100 μL was measured by colorimetric absorbance spectroscopy at 540 nm. After subtracting a baseline absorbance from blank controls, the absorbance was used to normalize the fluorescence data from each well.

Quantification of hydrogen sulfide levels using MitoA *in vivo* exomarker

MitoA and MitoN were quantified in mouse blood using LC-MS/MS. Mice received a tail vein IV injection of 50 nM MitoA in 0.9% saline (100 μL). MitoA was given 1.5 hr to distribute into mitochondria. Mice were culled by decapitation 90 minutes after administration.

Liver was excised and flash frozen in liquid nitrogen. MitoA and MitoN were extracted from tissue by homogenization of liver (50 mg) enriched with 5 pg d15-MitoN (95% ACN, 210 μL) which was used as an internal standard (IS). Homogenates were centrifuged (16,000 g, 10 min, RT) and the supernatant was transferred to a clean tube and stored on ice. The pellet was re-extracted (95% CAN, 210 μL), spun down again (16,000 g, 10 min, rT) and the supernatants were combined and incubated at 4°C for 30 mins. Calibration standards comprise MitoA and MitoN standards ranging from 0.01 to 10 pg in 500 μL 95% ACN. 500 μL of the supernatants and calibration standards were loaded onto an ISOLUTE PLD+ protein and phospholipid removal plate (Biotage, Sweden). Samples and standards were pulled through the plate under vacuum into a 2 mL deep-well 96-well plate. Wells were dried completely at 40°C under N_2 and resuspended in 100 uL 20% ACN, 0.1% FA. The plate was shaken at (250 rpm, 20 min) to ensure reconstitution. Liquid chromatography-Mass Spectrometry was performed on an I-class Acquity LC system-Xevo TQS triple quadrupole mass spectrometer (Waters, Warrington, UK). Samples were kept at 10°C and injected onto an Acquity UPLC BEH C18 column fitted with a 0.2 μm filter (1 × 50 mm, 1.7 μm, Waters). Chromatographic separation of MitoA and MitoN was achieved using mobile phase A composition: water:ACN, (95:5, 0.1% FA), mobile phase B: ACN:water (90:10, 0.1% FA). LC mobile phases were infused at 200 μL/min using the gradient: 0– 0.3 min, 5% B; 0.3–3 min, 5%–100% B; 3– 4 min, 100% B, 4.0– 4.10, 100%–5% B; 4.10– 4.60 min, 5% B. MS/MS analysis was performed under positive ion mode (Source spray voltage, 3.2 kV; cone voltage, 125 V; ion source temperature, 100°C). Curtain and collision gas were nitrogen and argon, respectively. Analytes were detected by multiple reaction monitoring (MRM). MitoA undergoes neutral loss of N2 to a nitrene (precursor ion). For quantification the following transitions were used: MitoA, *m/z* 437.2 →183.1; MitoN, *m/z* 439.2 → 120.0; d15-MitoN, 454.2 → 177.1 m/z. MassLynx 4.1 software was used to integrate the peak area of the analytes MitoA, MitoN and the d15-MitoN internal standard. Response was calculated by normalizing sample peak areas to the IS peak area. By comparison of sample responses to calibration standard responses the mass of each analyte in the tissue sample was calculated. The mass of analyte was normalized to the mass of tissue homogenizer and MitoN/MitoA ratio was calculated.

Preparation of hepatic mitochondria

Fresh liver was taken from mice, and homogenized in 250 mM sucrose, 10 mM HEPES, 1 mM EGTA. 0.5% fatty acid free bovine serum albumin (BSA) pH 7.4 at 4°C, with seven passes of a loose glass Dounce homogenizer (Type A). Homogenates were centrifuged in glass tubes at 2900 rpm (1000 g) for 10 min in a pre-chilled 4°C Beckman centrifuge (JA-20 Fixed angle rotor). The supernatant was then centrifuged in glass tubes at 8500 rpm (8700 g) for 10 min at 4°C. The supernatant was aspirated and any visible lipid was carefully removed from the sides of the tubes. The pellet was washed with 5 mL of mIR-05 buffer (0.5 mM EGTA, 3 mM $MgCl_2$, 20 mM taurine, 10 mM KH_2PO_4, 20 mM HEPES, 110 mM sucrose, 1 mg/ml fatty acid free BSA, pH 7.2), and centrifuged at 8500 rpm (8700 g) for 10 min at 4°C. After aspiration and removal of visible lipid, the pellet was suspended in 1ml of mIR-05 buffer and kept on ice until used. All measurements were taken within two hours of preparation. Protein concentration was determined using the DC-Protein Assay (BioRad) as per manufacturers instruction.

Amperometric analysis of sulfide disposal

Hepatocytes were prepared as described, and kept at room temperature in DMEM with 5.5 mM glucose, 10% FCS, 4 mM glutamax or 7 mM glutamine, and antibiotics at a concentration of 4×10^6 per ml. Mitochondria were prepared as described, and maintained on ice in mIR-05 buffer until use. All samples were analyzed within 4 hours of preparation. Sulfide ($H_2S_{(g)}$) was measured (with and without samples) in a 2ml volume plastic chamber, to which an amperometric sensor was inserted, sealed with a rubber O-ring. Voltage measurements from the sensor (linear relationship to $H_2S_{(g)}$ concentration) were recorded using a TBR4100 Gas radical analyzer (World Precision Instruments). A gas permeable membrane covered the sensor, and the outer glass sensor compartment was filled with H_2S detection fluid (World Precision Instruments). All measurements of $H_2S_{(g)}$ from standards and samples were recorded as voltage by the amperometric sensor at ambient temperature. Mitochondrial measurements (and standards) were taken in serum free mIR-05 buffer. Hepatocyte measurements (and standards) were taken in serum-free, bicarbonate-free DMEM, buffered with 25 mM HEPES (pH 7.4), with 5 mM glucose, 2 mM glutamax and 2 mM pyruvate. Sulfide was added to buffer in the form of Na_2S, predicted to equilibriate according to its Pka at this pH to about 1/3 of sulfide as $H_2S_{(g)}$ 2/3 as HS^-. The probes selectivity to $H_2S_{(g)}$ (versus HS^-) was confirmed with standards by demonstrating predicted signal amplification to a maximum following acidification of media to pH < 5 (approx. 100% $H_2S_{(g)}$/0% HS^-), and signal compression to a minimum following alkalinisation of standard to pH > 10 (Approx 0% $H_2S_{(g)}$/100% HS^-/S^{2-}). A final re-acidification recovered the signal to near maximal levels. Standard curves for calculating experimental measurements were prepared using freshly made Na_2S solutions ranging from 0.25 – 20 μM (corresponding to approximately 170 nm – 6.7 μM $H_2S_{(g)}$). $H_2S_{(g)}$ disposal was measured by recording the extrapolated $H_2S_{(g)}$ concentration after 10 min incubation with samples. A baseline without sample was taken for 5 min, and then after sample addition (400,000 hepatocytes, or 1.6 – 2.0 mg of mitochondrial prep), another 5 min baseline with sample was taken. In all experiments, no detectable increase in signal (limit of detection 0.25 μM Na_2S) was observed during incubation of hepatocyte or mitochondrial samples from either genotype. Following addition of 10 μM Na_2S the (voltage) signal was recorded over a period of 10 min. Disposal rates were calculated after subtraction of a baseline disposal rate in media alone, over a 10 min period, performed each day of experimentation. Sample disposal rates were in the range of 5-20 higher than baseline disposal rate confirming good signal to noise. To determine the rate of disposal that is dependent upon respiration, a fresh aliquot of the same sample was prepared as before, but 5 min after addition of sample to chamber, Antimycin A (2.5 μM, dose titrated) was added. After a further 5 minutes, 10 μM Na_2S was added and a disposal rate (after subtraction to sample free baseline rate) was again calculated. The respiratory (Antimycin sensitive/complex III dependent) sulfide

disposal rate of samples was calculated as the difference between the nieve sample rate and the Antimycin inhibited rate. After each measurement, the sample was removed, and centrifuged to collect cells or mitochondria for a final protein assessment (DC-Protein Assay, Bio-Rad) for the purposes of normalization.

Mitochondrial ROS (MitoSOX) measurement in H_2O_2 treated hepatocytes

Hepatocytes were seeded overnight onto 96-well collagen coated plates. Cells were exposed to a range of concentrations of H_2O_2 (0.125 - 8 μM) for 2 hours. Following 3 washes with PBS, cells were incubated with MitoSOX Red mitochondrial superoxide indicator (Thermo Fisher) for 10 mins prior to three further washes. Measurement of fluorescence was carried out in a fluorescence detector plate reader (TECAN), using 510 nm for excitation and 580 nm for emission detection. Data from each well was normalized to sulfurhodamine dye protein stain.

Persulfidation Mass Spec and GO term analysis

Livers from mice were removed promptly following decapitation (within 2 min), and snap frozen in liquid nitrogen. The persulfide proteome analysis using the BTA method was conducted as described previously (Gao et al., 2015). Briefly, 100-150 mg of frozen liver tissue was pulverized and lysed on ice in RIPA buffer (100 mM Tris, pH 7.5, 150 mM NaCl, 2mM EDTA, 1% Triton X-100, 25 mM deoxycholic acid, 2 tablets/ 100 mL of cOmplete, Mini, EDTA-free Protease Inhibitor Cocktail (Roche)). The lysates were centrifuged at 14,000 g for 10 min at 4°C and protein concentrations were determined using the Bradford reagent (BioRad). Supernatant containing 6 mg of protein was incubated with 100 μM NEM-biotin (Pierce) for 60 min at room temperature after which the proteins were precipitated with cold acetone (1:4 v/v) for 1 h at −20°C, followed by a centrifugation at 14,000 g for 10 min at 4°C. The precipitated protein was re-suspended in a denaturing buffer containing 7 M urea, 1% SDS, 150 mM NaCl, 100 mM Tris, pH 7.5. Then, the samples were diluted 10-fold with trypsin reaction buffer (1 mM $CaCl_2$, 100 mM Tris pH 7.5) and incubated overnight with sequencing grade modified trypsin (1:50 trypsin:protein) (Promega) at 30°C. The digestion products were mixed with streptavidin-agarose beads (Thermo-Scientific) and incubated at 4° overnight, followed by ten washes with the wash buffer (0.1% SDS, 100 mM Tris, pH 7.5, 600 mM NaCl, 1 mM EDTA, 1% Triton X-100). The streptavidin-agarose bound peptides were incubated with elution buffer (100 mM Tris, pH 7.5, 150 mM NaCl, 1 mM EDTA, 30 mM DTT) for 1 hr at room temperature. Persulfidated peptides were eluted by centrifugation and derivatized with 40 mM iodoacetamide for 2 hr at room temperature in the dark. The samples were then passed through a desalting column (Pierce). LC-MS/MS analysis was carried out using an LTQ-Orbitrap Elite mass spectrometer (Thermo-Fisher) coupled to an Ultimate 3000 high-performance liquid chromatography system. The alkylated peptides were loaded onto a 75 μm desalting column, C18 reverse phase resin (Dionex), and eluted onto a Dionex 15 cm x 75 μm id Acclaim Pepmap C18, 2 μm, 100 Å reverse-phase chromatography column using a gradient of 2%–80% buffer B (5% water, 95% acetonitrile, 0.1% formic acid) in buffer A (0.1% formic acid). The peptides were eluted onto the mass spectrometer at a flow rate of 300 nl/min and the spray voltage was set to 1.9 kV.

GO enrichment analysis

In order to identify differentially persulfidated proteins between the C57BL/6J and *Tst*$^{-/-}$ samples, we compared the abundances of persulfidated fragments in appropriately treated mass spectrometry datasets to the estimated overall abundance of the corresponding parent proteins in standard label-free quantitation experiments. For each observed persulfidated fragment in each experimental replicate, we calculated the persulfidation rate as the $\log_2$ ratio of the count of that persulfidated fragment to the median count of that fragment across all experimental replicates. The observed counts for the *Tst*$^{-/-}$ replicates were additionally scaled (prior to log transformation) by the ratio of abundances of the parent protein between the C57BL/6J and *Tst*$^{-/-}$ cells to normalize for differential protein abundance across conditions. For each peptide we then assigned an approximate average $\log_2$ fold change in persulfidation rate between the C57BL/6J and *Tst*$^{-/-}$ conditions. If a persulfidated peptide was identified in at least two biological replicates of one condition and none in the other, we assigned a $\log_2$ fold change of ± 5.0 as placeholder values indicating a high confidence change; peptides with only one observation in one condition and none in the other were omitted from our analysis. Having thus obtained estimates for the magnitude of changes in persulfidation rate of each detectable peptide, we then performed gene ontology term enrichment analysis using the estimated $\log_2$ fold changes. We consolidated the peptide-level data to protein-level data by taking the largest magnitude change in persulfidation levels across all peptides from a given protein, and then used the iPAGE program [https://dx.doi.org/10.1016/j.molcel.2009.11.016] to identify GO terms with significant mutual information with the profile of persulfidation rates. Arguments to iPAGE were "—max_p=0.1 –minr=0.3 –ebins=9 –exptype=continuous," indicated that the data were discretized into nine equally populated bins prior to analysis, and that default hypergeometric p value and information content thresholds were relaxed to maximize sensitivity.

Focused analysis of persulfidation in gluconeogenesis proteins

The gluconeogenesis pathway was selected for a focused analysis of the persulfidation rate of all cysteine sites detected in the mass spectrometry data as described above. All peptides from proteins present in the persulfidation dataset used for GO enrichment analysis, that are defined by the GO term gluconeogenesis (GO 0006094) were included, these were Pgk1, Gpi1, Fbp1 and Tpi1 (22 peptides). The $\log_2$ rate ratio of persulfidation (*Tst*$^{-/-}$ /6J) of all of these 22 peptides was compared first to the entire mass spectrometry dataset for $\log_2$ rate ratio of persulfidation (1245 peptides after removal of ambiguous peptides, peptides with a P-diff of 0, and the 22 gluconeogenesis peptides). A Mann-Whitney non parametric t test was used to detect significance. A second analysis was

performed with the gluconeogenesis pathway. For this analysis, all log_2 rate ratio's were given a positive sign to indicate the magnitude of change in the $Tst^{-/-}$ relative to 6J, independent to the direction of change. A Mann-Whitney non parametric t test was then performed to determine if the magnitude of change in persulfidation in the gluconeogenesis pathway was significantly higher than that of the overall the dataset.

Persulfidation labeling and western blotting from frozen liver

80-120 mg of frozen liver samples were homogenized on ice using a 2 mL glass Dounce homogenizer (Kimble), in 500 μl buffer (7 M urea, 100 mM Tris pH 7.5, 150 mM NaCl, 1 mM EDTA, 1% Triton X-100, 1% deoxycholic acid; supplemented with cOmplete Protease Inhibitor Cocktail (Roche)). Initial disruption of tissue was achieved with three passes, using the loose (Type A) pestle, and after 5 min incubation on ice; complete homogenization was achieved with 9 passes using the tight fit (Type B) pestle. Homogenates were centrifuged at 5000 rpm (2655 g) for 5 min at 4°C. Protein concentrations of supernatants were determined using the *DC* BCA protein assay (Bio-Rad). Protein (6 mg) from each sample, was made up to 1 mL with phosphate buffered saline (pH 8.0). Freshly prepared EZ-link Maleimide PEG Biotin EZ-linker (Thermo Fisher 21902BID), was added to samples to 100 μM, and incubated for 1 hour at room temperature with gentle mixing. Excess maleimide linker was removed from samples by acetone precipitation (3 volumes) at −20°C for 1 h, followed by centrifugation at 12000 rpm (17390 g) for 10 min at 4°C. Protein pellets were washed with ice-cold acetone and then dissolved in 250 μl of 50 mM Tris pH 8.0, 100 mM NaCl, 1 mM EDTA, 1% SDS. To each sample, 750 μl of RIPA buffer (100 mM Tris pH 7.5, 150 mM NaCl, 1 mM EDTA, 1% Triton X-100, 1% deoxycholic acid) was then added. An aliquot (20 μl) was taken from each sample for estimation of total input protein for normalization (described below). The remainder of the samples were split into duplicates and incubated with gentle mixing, overnight at 4°C with 320 μl of pre-washed streptavidin agarose beads (Thermo Fisher 20347). Beads were washed 10 times with 0.8 mL washing buffer (30 mM Tris pH 7.5, 600 mM NaCl, 1 mM EDTA, 1% Triton X-100, 0.1% SDS), followed by one wash with phosphate buffered saline (pH 7.4). Beads were then centrifuged for 1 min at 1000 rpm (106 g) to dry. Elution of the duplicate samples for western blot analysis was performed by adding 300 μl of elution buffer (30 mM Tris pH 7.5, 150 mM NaCl). For each sample pair, one duplicate was eluted in buffer supplemented with 10 mM DTT and the other without DTT. The beads were incubated with the elution buffer for 1 hour at RT; and centrifuged for 1 min at 1000 rpm (106 g) to collect the eluate. Each eluted sample was concentrated to 20 ul using an Ultra-0.5 Centrifugal Filter Device, 10 K cut-off (Amicon), as per the manufacturer's instructions. Eluted samples, and input protein samples were loaded in their entirety onto SDS-PAGE gels and transferred overnight at 4°C by western blotting onto PVDF membrane. Total protein from each lane on the membrane was estimated after staining with REVERT total protein stain (LICOR) according to the manufacturer's instructions. Briefly, following overnight transfer and after rinsing the blot with water, the membranes were incubated with REVERT total protein stain for 5 min, and rinsed twice with wash solution. Blots were then imaged in the 700 nm channel with an Odyssey imaging system (LICOR). Each lane was measured for its total integrated fluorescence intensity to obtain an estimate of the total protein in each lane. Measurements from no-DTT eluted sample lanes were subtracted from DTT eluted sample lanes. Similar fluorescence measurements of input total protein lanes were used to normalize the eluted sample measurements, and this was used as a measure of relative protein-persulfidation rate.

Mass spec analysis of liver protein

Sample preparation; $Tst^{-/-}$ and wild-type (C57BL/6J) mouse strains were fed either high-fat (58% fat) or normal (low fat) diet. Livers from mice were removed following decapitation, and snap frozen in liquid nitrogen. Four biological replicates from the 4 conditions were used to isolated proteins and performed protein quantitation using iTRAQ 8plex. Liver tissue was homogenized using 1 mL of 8 M urea with HEPES buffer pH 8.0. The protein concentration was determined using the Bio-Rad RC DC protein assay kit (Bio-Rad, Hercules, CA, USA). One hundred micro grams of protein from each of the samples were reduced with THP (Tris(hydroxypropyl)phosphine), alkylated with MMTS (methyl methanethiosulfonate) in 500 mM triethylammonium bicarbonate (TEAB, pH 8.5), trypsin digested and subsequently label with iTRAQ 8plex accordingly to the manufacturer's instructions. *Electrostatic Repulsion-Hydrophilic Interaction Chromatography (ERLIC) Peptide fractionation;* Peptide fractionation was performed using a pH gradient. Labeled peptides were dissolved in 100 μL of buffer A (100 mM formic acid, 25% acetonitrile, pH 3.0), followed by fractionation in a 2.6 × 200 mm, 5 μm, 200 Å PolySulfethyl A column (Poly LC Inc., Columbia, MD), using an Ultimate 3000 UHPLC+ focused (Thermo-Fisher Scientific) system, operating at a flow rate of 0.2 ml/min. Twenty minutes of isocratic buffer A were followed by a linear gradient from 0% to 100% buffer B (100 mM ammonium formate, 25% acetonitrile, pH 6.0) over 20 min and then a final linear gradient from 0% to 100% buffer C (600 mM ammonium acetate, 25% acetonitrile, pH 6.0) over 10 min. A total of 22 fractions (1-min intervals) were collected. All fractions were lyophilized and stored at −20°C. *Nanoflow Liquid Chromatography Tandem Mass Spectrometry;* NanoLC MS/MS analysis was performed using an on-line system consisting of a nano-pump UltiMate 3000 UHPLC binary HPLC system (Dionex, ThermoFisher) coupled with Q-Exactive mass spectrometer (ThermoFisher, San Jose, CA. iTRAQ-labeled peptides were resuspended in 2% ACN, 0.1% formic acid (20 μL) and 6 μL injected into a pre-column 300 μm × 5 mm (Acclaim PepMap, 5 μm particle size). After loading, peptides were eluted to a capillary column 75 μm × 50 cm (Acclaim Pepmap, 3 μm particle size). Peptides were eluted into the MS, at a flow rate of 300 nL/min, using a 90 min gradient from 0% to 35% mobile phase B. Mobile phase A was 2.5% acetonitrile with 0.1% formic acid in H_2O and mobile phase B was 90% acetonitrile with 0.025% trifluoroacetic acid and 0.1% formic acid. The mass spectrometer was operated in data-dependent mode, with a single MS scan in the orbitrap (400-2000 m/z at 70 000 resolution at 200 m/z in profile mode); automatic gain control (AGC) was set to accumulate 4×10^5 ions, with a maximum

injection time of 50 ms. MS/MS scans were performed in the orbitrap at 17 500 resolution. Ions selected for MS/MS scan were fragmented using higher energy collision dissociation (HCD) at normalized collision energy of 38% with an isolation window of 0.7 m/z. MS2 spectra were acquired with a fixed first m/z of 100. The intensity threshold for fragmentation was set to 50 000 and included charge states 2+ to 7+. A dynamic exclusion of 60 s was applied with a mass tolerance of 10 ppm. *Data Analysis;* Raw files were converted to MGF files and searched against the mouse UniProt database (81033 sequences, released on March 2014) using MASCOT Version 2.4 (Matrix Science Ltd, UK). Search parameters were peptide mass tolerance of 10 ppm, and MS/MS tolerance of 0.05 amu allowing 2 missed cleavage. iTRAQ8plex (N-term) and iTRAQ8plex (K) were set as fixed modification, and acetyl (Protein N-term), Methylthio (C) and Oxidation (M) were allowed as variable modification. Peptide assignments with ion score cut-off of 20 and a significance threshold of $\rho < 0.05$ were exported to Excel for further analysis. Data are available from the ProteomeXchange with identifier PXD028909.

GO and KEGG enrichment analysis of proteome data

The data generated from the initial mass spectrometric analysis of iTRAQ labeled peptides from the 16 liver samples was analyzed by FIOS. A total of 16 samples were QC analyzed using the arrayQualityMetrics Bioconductor package to identify sub-standard and/or outlier samples. No samples were identified as outliers. All samples passed the manual and automated quality control based on three metrics (MAplot, Boxplot and Heatmap). The exploratory analysis using PCA showed that the samples clustered perfectly into four groups based on the factor Group (representing four genotype-diet combinations). The first PC captures the main source of variation in the dataset and is showing a separation of the samples based on diet, where high-fat diet and control diet samples separate. The second PC shows a separation between genotypes (Tst KO and WT). The hierarchical clustering and PCA plot both show a clear separation based on the iTRAQ labels. This is expected as the iTRAQ labels are confounded with the Groups. While the observed separation of the samples into groups is most likely due to the underlying biological differences, any technical variations (potentially introduced during the wet lab processing) could be masked. The log2 ratio data were subsequently normalized within arrays using loess, followed by normalization between samples using the Gquantile method. A total of 4 single and/or multi-factor comparisons, using statistical approaches, were performed. The contrast "Tst KO vs WT mice (High-fat diet)" was analyzed at a cut-off (unadjusted) p value < 0.01. Due to the known bias in fold-change magnitudes of the iTRAQ technology, no fold-change cut-off was applied to the significant differentially abundant proteins. With this threshold 551 proteins were differentially abundant in at least one of the comparisons. The contrast "High-fat diet vs Control diet (WT)" had the most DAPs (432) while the contrast " *Tst*$^{-/-}$ versus 6J mice (High-fat diet)" had the least DAPs (83). Noticeably, the TST protein showed the strongest downregulation for both of the contrasts comparing *Tst*$^{-/-}$ to 6J mice, consistent with gene deficiency and the fold change compression effect of iTRAQ. The full dataset (4,322 identified proteins) was filtered to remove proteins having less than two detected peptides (on average across all 16 samples); leaving 1,654 proteins for downstream analysis. Exploratory analysis using principal component analysis (PCA) showed that the dataset separated into four distinct groups based on the genotype-diet combinations along the two first principal components (PCs). These 1,654 proteins were used for enrichment analysis for GO terms and KEGG pathways. Individual proteins were considered of interest if they were found significantly different ($p < 0.01$) between selected pairwise comparisons. The four comparisons were *Tst*$^{-/-}$ normal diet versus C57BL/6J normal diet, C57BL/6J high fat diet versus C57BL/6J normal diet, *Tst*$^{-/-}$ high fat diet versus *Tst*$^{-/-}$ normal diet, and *Tst*$^{-/-}$ high fat diet versus C57B/6J high fat diet. Normalized mean abundance of proteins was expressed as Log2 fold change ratios for each comparison.

Transcription factor enrichment analysis

43 upregulated proteins were selected for analysis of their promoter sequences (selected on basis of P value < 0.05; adjusted for comparison of diet and genotype). 67 control proteins were selected from the proteome data on the basis of their equivalence of abundance between C57BL/6J and *Tst*$^{-/-}$. We used a QIAGEN hosted/SABiosciences mouse database of promoter located transcription factor binding sites. 34 transcription factors were chosen to analyze, based on either their a-priori prevalence in the promoter of *Tst*$^{-/-}$ upregulated proteins (present in the promoters of more than 50% of the upregulated proteins) or on their links to either sulfide or nutrient metabolism. The proportion of genes containing a TFBS was calculated for the upregulated set (43) and the control set (67). The ratio of upregulated to control was then calculated. The number of genes with and without the presence of the TFBS were analyzed for establishing statistical difference (Upregulated versus Control), using a Fisher Exact test ($p < 0.05$).

NRF2 target identification and proteome analysis

NRF2 target genes of the mouse liver were compiled from the following reviews (Cuadrado et al., 2019; Tonelli et al., 2018; Rooney et al., 2018; Walsh et al., 2014). 106 genes were identified as target genes (upregulated at mRNA or protein level following NRF2 activation). 47 of these target genes were represented in our liver proteome, and each protein was checked for relative expression between *Tst*$^{-/-}$ and 6J (on normal diet, threshold of $p < 0.01$). 10 of the 47 target genes were lower in abundance in the *Tst*$^{-/-}$ proteome, 37 unchanged, with none upregulated. To analyze whether this was statistically significant, we compared this to the percentage of proteins upregulated, downregulated or unchanged in the proteome database. 5.86% of proteins were upregulated, 5.62% downregulated, and 88.6% unchanged in the full database (1654 proteins total). Expected (mean) numbers of proteins from a hypothetical set of 47 proteins, predict rounded values of 3 upregulated, 3 downregulated and 42 unchanged. We used these as a reference to the actual data for NRF2 target proteins; 0 upregulated, 10 downregulated and 37 unchanged. A Freeman-Halton

Fisher Exact test was used for analysis of significance, and a significant difference between predicted and actual distribution was found ($P_A = 0.039$, $P_B = 0.047$).

Electron micrograph imaging

Liver tissue for transmission electron microscopy was prepared following immersion fixation in 0.1 M PB buffer (pH 7.4, EM-grade) containing 4% paraformaldehyde and 2.5% glutaraldehyde. 1mm tissue blocks were post-fixed in 1% osmium tetroxide in 0.1 M PB for 45 min before dehydration through an ascending series of ethanol solutions and propylene oxide. Tissue blocks were then embedded in Durcupan before ultrathin sections (~60/70 nm) were cut and collected on formvar-coated grids (Agar Scientific, UK), stained with uranyl acetate and lead citrate in an LKB Ultrostainer and then quantitatively assessed in a Philips CM12 transmission electron microscope (TEM).

Seahorse respiratory analysis

Primary hepatocytes (C57BL/6J and *Tst*$^{-/-}$ mice) were seeded immediately following purification onto collagen coated V7 Seahorse 24-well cell culture microplates (Agilent Technologies), in 200 μL medium (DMEM, 5.5 mM glucose, 10% FCS, 7 mM glutamine, and penicillin/streptomycin antibiotics), for culture in a 5% CO_2 37°C incubator. Experiments were performed between 22-28 hours following collection from mice. Optimization experiments determined the optimal seeding density, which was then standardized at 10,000/well. Optimization for drugs and compounds used in Seahorse experiments were performed separately with hepatocytes for each genotype and dietary regime (normal or 58% high fat). This established the doses of drugs for respiratory manipulation, which were the same for both genotypes and diets; oligomycin (2 μM), FCCP (0.5 μM), and antimycin/rotenone (1 μM/0.2 μM). In all experiments, overnight media from cells was replaced, after two washes (0.75 ml), with 525 μL of run media and incubated for 30 mins at 37°C (without CO_2), prior to entry into the Seahorse XFe24 Extracellular Flux Analyzer (Agilent). The analyzer was operated using Wave software (Agilent), and all oxygen consumption rate (OCR) data was normalized to protein using the Sulforhodamine B stain (described above). Data from each biological replicate was averaged from between 4-10 replicate wells, to produce a single value at each measurement time for each biological replicate. Respiratory parameters were calculated as described below for each biological replicate, and this data was used for statistical analysis of genotype effects.

Mitochondrial stress test (MST)

Run media for the MST was Seahorse assay media (Agilent), supplemented with 10 mM glucose, 2 mM sodium pyruvate, pH 7.35 ± 0.5 at 37°C). Most measurements were made using 3 min mixing, 2 min wait, 3 min measure. Measurements following addition of FCCP to hepatocytes from high fat fed mice were measured using 4 min mix, 2 min wait, 2 min measure. Three measurements were taken basally, and three measurements taken after injection of each drug (in sequence; oligomycin for inhibiting ATP-linked respiration, FCCP for eliciting maximal uncoupled respiration, antimycin/rotenone for inhibiting the respiratory electron chain). Respiratory parameters for each biological replicate were calculated from the mean normalized OCR as follows. *Basal respiration* was calculated by subtracting the third OCR measurement following injection of antimycin/rotenone (12th measurement) from the third basal OCR measurement (3rd measurement). *ATP linked respiration* was calculated by subtracting the third OCR measurement following the injection of oligomycin (6th measurement) from the third basal OCR measurement (3rd measurement). *Maximum (uncoupled) respiration* was calculated by subtracting the third OCR measurement after injection of antimycin/rotenone (12th measurement) from the first measurement (peak OCR) following injection of FCCP (7th measurement). *Proton leak respiration* was calculated by subtracting the third OCR measurement after injection of antimycin/rotenone (12th measurement) from the third measurement following the injection of oligomycin (6th measurement). *Non-respiratory OCR* was taken from the third measurement after the addition of antimycin/rotenone (12th measurement).

Octanoate rescue test

To investigate lipid respiratory metabolism, hepatocytes were prepared, seeded and cultured overnight as above. Run media for the Octanoate rescue was Seahorse assay media (Agilent), supplemented with 5 mM glucose, 0.1 mM sodium pyruvate, 1 mM sodium lactate, and 0.5 mM carnitine pH 7.35 ± 0.5 at 37°C). All measurements were made using 3 min mixing, 2 min wait, 3 min measure. After washing cells into run media, and 30 min before entry into the analyzer, half of the wells from each genotype were incubated with 8 μM etomoxir (or DMSO vehicle) to block carnitine dependent import of long chain fatty acids into the mitochondria. Three basal measurements were taken prior to injection of oligomycin, two measurements were taken prior to FCCP, two measurements were taken prior to injection of sodium octanoate (250 μM), three measurements taken prior to antimycin/rotenone followed by two final measurements. Standard respiratory parameters were calculated analogous to the above description for the standard mitochondrial stress test, except using the second measurement following injection of drug when only 2 measurements were taken. Dependency upon endogenous fatty acids for supporting uncoupled respiration (*Etomoxir inhibited respiration*) was calculated for each biological replicate using the maximal respiration prior to the addition of octanoate. Maximal respiration was calculated as the 6th measurement (peak FCCP OCR) – 12th measurement (lowest Antimycin/Rotenone OCR). The mean maximal respiration from the etomoxir treated wells was subtracted from the mean maximal respiration of the vehicle treated wells to calculate the Etomoxir inhibited respiration (long chain fatty acid dependency) for that biological replicate. Octanoate stimulation of respiration (*Octanoate stimulated*

respiration), was calculated for each vehicle well by subtracting the second OCR measurement after injection of FCCP (7th measurement) from the third measurement after injection of octanoate (10th measurement).

Real time for mRNA analysis

RNA extraction, cDNA synthesis and real-time PCR were performed as described (Morton et al., 2011; Moreno-Navarrete et al., 2013). Probes were mouse *Mpst*, Mm00460389_m1, *Tst*, Mm00726109_m1; *Gapdh* (internal control), Mm99999915_g1; and *Tbp* (internal control), Mm0000446973_m1.

QUANTIFICATION AND STATISTICAL ANALYSIS

For analytes, bioenergetics, fluorescent probes, gene expression, and protein levels, generally group sizes of 6 were calculated to allow detection of differences in these variable parameters to a threshold of 15% (there is sufficient power to detect smaller differences in certain parameters with this cohort size) with a power of at least 0.8. In some studies, limitations in animal numbers, or fewer remaining samples from larger group sizes resulting from their use for multiple end-points, precluded the desired minimum of n = 6 per group. Protein or mRNA differences in validation studies with 2 parameters (e.g., diet with line or genotype) were analyzed using 2-way ANOVA for line and diet effects followed, where appropriate, by post hoc Tukey tests or Holm-Sidak multiple comparison tests using Sigmastat version 3.5 (Systat Software) or Prism (Graphpad Software). For simple 2 condition comparisons, t test was used. For simple control versus treated (including different treatments or concentration response curves) data were analyzed by 1-way ANOVA. For longitudinal measures (e.g., PTT, ITT, bodyweight gain) repeated-measures (RM) ANOVA was used and multiple comparisons determined. For all main *in vivo* studies, a blinding strategy was used where the operator (e.g., for injections of glucose, or administration of drug) was blind to the genotype of the subject during the experiment. Similarly, for analysis of images (e.g., oil-red O staining) the scorer was blind to genotype and the data coded, with the code broken by a second individual. Downstream analysis of e.g., tissue mRNA and protein content was not generally blinded to allow appropriate data arrangement on e.g., representative western blots. For clamp studies, mean ± standard error of mean (sem) will be presented, statistical analysis will use t test to investigate differences of genotype on each diet (2 independent experiments, normal diet, and high fat diet, are not compared directly to each other). Statistical significance and the number (n) of subjects or samples for analysis are indicated in the figure legends.

Cell Reports, Volume *37*

Supplemental information

The hepatic compensatory response to elevated systemic sulfide promotes diabetes

Roderick N. Carter, Matthew T.G. Gibbins, Martin E. Barrios-Llerena, Stephen E. Wilkie, Peter L. Freddolino, Marouane Libiad, Victor Vitvitsky, Barry Emerson, Thierry Le Bihan, Madara Brice, Huizhong Su, Scott G. Denham, Natalie Z.M. Homer, Clare Mc Fadden, Anne Tailleux, Nourdine Faresse, Thierry Sulpice, Francois Briand, Tom Gillingwater, Kyo Han Ahn, Subhankar Singha, Claire McMaster, Richard C. Hartley, Bart Staels, Gillian A. Gray, Andrew J. Finch, Colin Selman, Ruma Banerjee, and Nicholas M. Morton

Figure S1. *Tst* mRNA tissue expression profile in C57BL/6J mice and the metabolic fates of $^{13}C_3$ pyruvate in hepatocytes from *Tst*$^{-/-}$ mice. Related to Figure 1

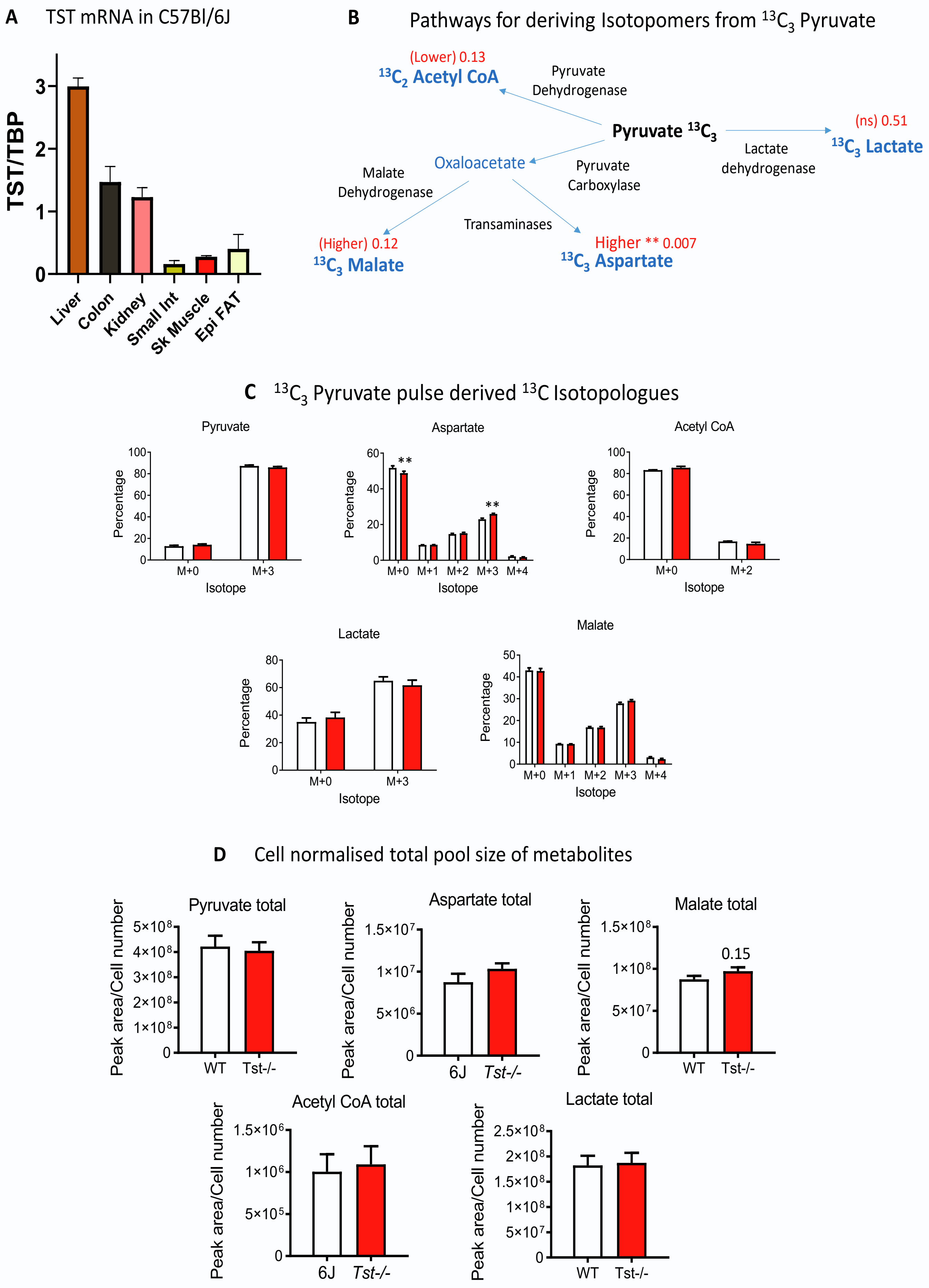

Figure S1. *Tst* mRNA tissue expression profile in C57BL/6J mice and the metabolic fates of $^{13}C_3$ pyruvate in hepatocytes from *Tst*$^{-/-}$ mice. Related to Figure 1 **(A)** Histogram showing *Tst* mRNA level across liver, colon, kidney, small intestine, skeletal muscle and epididymal fat from male C57BL/6J mice measured by realtime-PCR and normalised to *Tbp* mRNA **(B)** Diagram representing metabolites derived from pyruvate. Oxaloacetate was not detected but is indicated as an intermediate to production of malate or aspartate. The isotopologue shown on the diagram represents to most abundant detected following pulse with $^{13}C_3$ pyruvate. In red is the direction of change in hepatocytes of *Tst*$^{-/-}$ mice, with significance or P-values (when less than 0.2) from t-tests**.** (**C**) Histogram showing isotopologues derived from $^{13}C_3$ pyruvate from C57Bl/6J (white bars, n = 5) and *Tst*$^{-/-}$ (red bars n = 4) cultured hepatocytes. Data represents the amount of isotopologues detected by mass spectrometry, as a percentage of the total detected metabolite (total includes unlabelled ^{12}C and all detected ^{13}C isotopologues). Counts were first normalised to cell number. **(D)** Histograms showing the total pool size of each metabolite. Data is cell normalised mass spec counts from all isotopologues of the given metabolite, including the relevant unlabelled ^{12}C species. Data are represented as mean ± SEM. Each metabolite was analysed using a t-test, ** indicates that $P < 0.01$. P-values less than 0.2 are also indicated for showing potential trends.

Figure S2. Insulin–regulated metabolic parameters in liver and plasma of *Tst*$^{-/-}$ mice. Related to Figure 1.

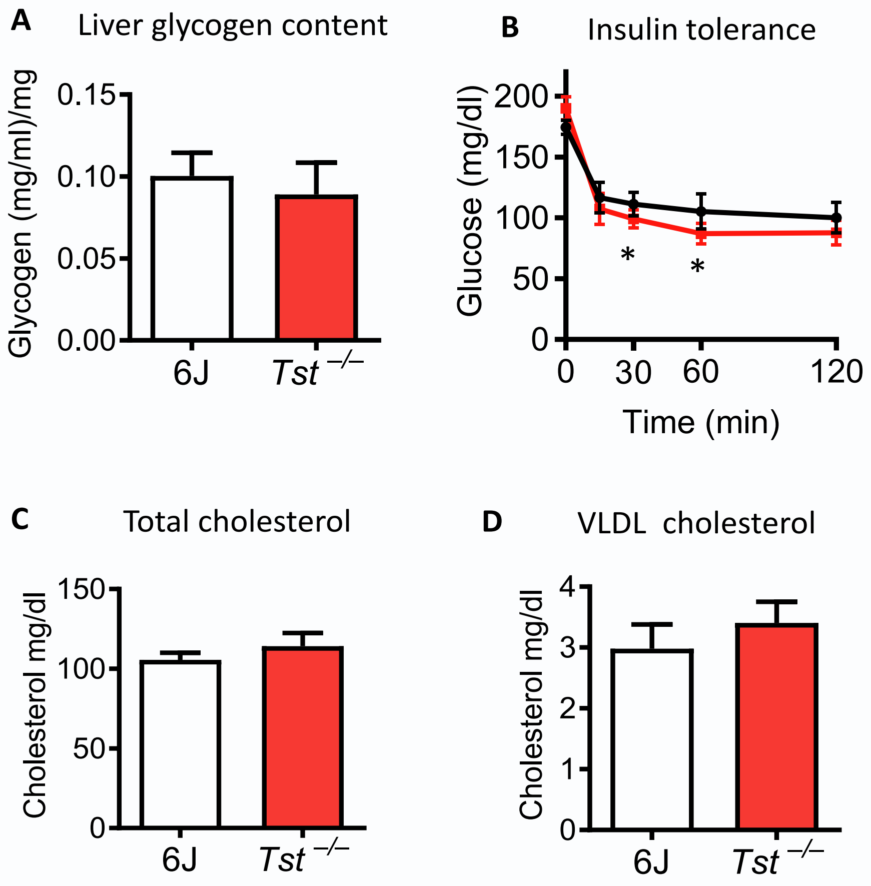

Figure S2. Insulin–regulated metabolic parameters in liver and plasma of *Tst*$^{-/-}$ mice. Related to Figure 1. (**A**) Glycogen measured from whole liver from normal diet-fed 4 hour fasted C57Bl/6J (6J; white bar, n = 5), and *Tst*$^{-/-}$ (red bar, n = 5) mice. Data are represented as mean ±SEM. (**B**) Plasma glucose (mg/dl), over 120 minutes following insulin administration (i.p., 1mU/g) in normal diet-fed 4 hour fasted C57Bl/6J (black line, n = 8) and *Tst*$^{-/-}$ (red line, n = 7) mice. (**C**) HPLC quantified total plasma cholesterol in normal diet-fed 4 hour fasted C57Bl/6J (white bar, n = 6) and *Tst*$^{-/-}$ (red bar, n = 6) mice. (**D**) HPLC quantified VLDL plasma cholesterol in normal diet-fed 4 hour fasted C57Bl/6J (white bar, n = 6), and *Tst*$^{-/-}$ (red bar, n = 6) mice. For (**B**) a Repeated Measures analysis demonstrated a significant effect of time (****) and an interaction between time and genotype (*). T-tests revealed that the decrement of glucose from baseline at 30 and 60 minutes after insulin was greater in the *Tst*$^{-/-}$ (*).

Figure S3. Hepatocytes from *Tst*$^{-/-}$ mice resist hydrogen peroxide induced mitochondrial reactive species accumulation. Related to Figure 2 and Table 1

A

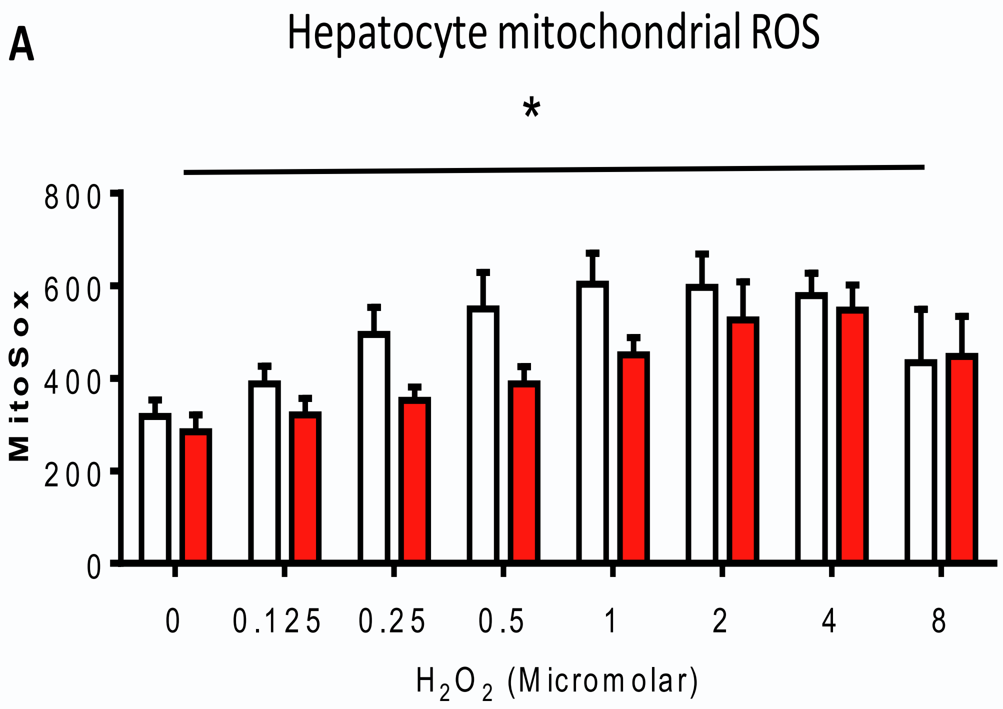

Figure S3. Hepatocytes from *Tst*$^{-/-}$ mice resist hydrogen peroxide induced mitochondrial reactive species accumulation. Related to Figure 2 and Table 1 **(A)** Mitochondrial reactive oxygen species measured from primary hepatocytes by MitoSox fluorescence from C57Bl/6J (white bars, n = 7) and *Tst*$^{-/-}$ (red bars, n = 7). Cells were exposed to a range of doses of H_2O_2 prior to MitoSox incubation and fluorescent detection. Data are represented as mean ±SEM. Significance was calculated using 2-WAY ANOVA for H_2O_2 dose and genotype. A significant effect of genotype is represented above the histogram with a *. H_2O_2 was significant to $P < 0.001$ (not represented on the histogram).

Figure S4. Persulfidation in the gluconeogenesis pathway is significantly different to global persulfidation patterns in the liver of the *Tst*$^{-/-}$ mice. Related to Figure 2 and Table 3.

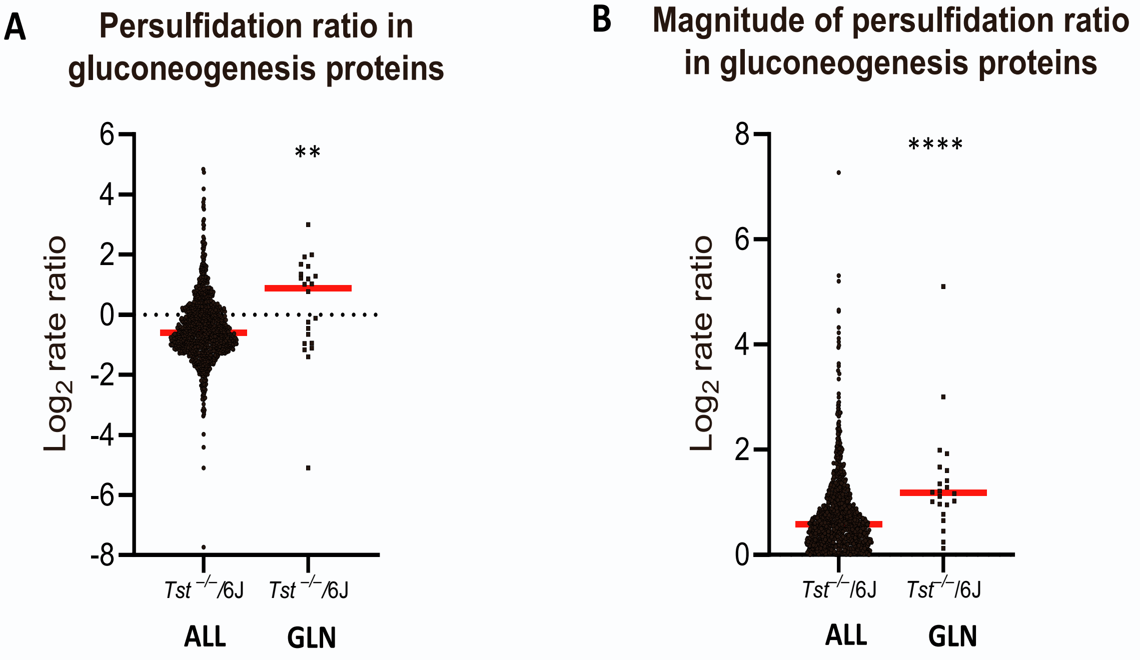

Figure S4. Persulfidation in the gluconeogenesis pathway is significantly different to global persulfidation patterns in the liver of the *Tst*$^{-/-}$ mice. Related to Figure 1, Figure 2 and Table 3. (**A**) Beeswarm plots showing the persulfidation $\log_2$ rate ratio (*Tst*$^{-/-}$ divided by 6J) for peptides in the entire data set (ALL), alongside the $\log_2$ rate ratio for peptides corresponding to proteins of gluconeogenesis (GLN). (**B**) Beeswarm plots showing the magnitude of the $\log_2$ rate ratio (independent to direction of change), for peptides in the entire data set (ALL), alongside the $\log_2$ rate ratios for peptides corresponding to proteins of gluconeogenesis (GLN). Data are represented as individual peptide $\log_2$ rate ratio values, with the median represented as a red line. Significance was calculated using the Mann-Whitney U non parametric T-test. ** $P < 0.01$, **** $P < 0.0001$.

Figure S5. Validation of proteomic profiles by select western blot is exemplified by increased mitochondrial MPST. Related to Figure 3 and Table 3.

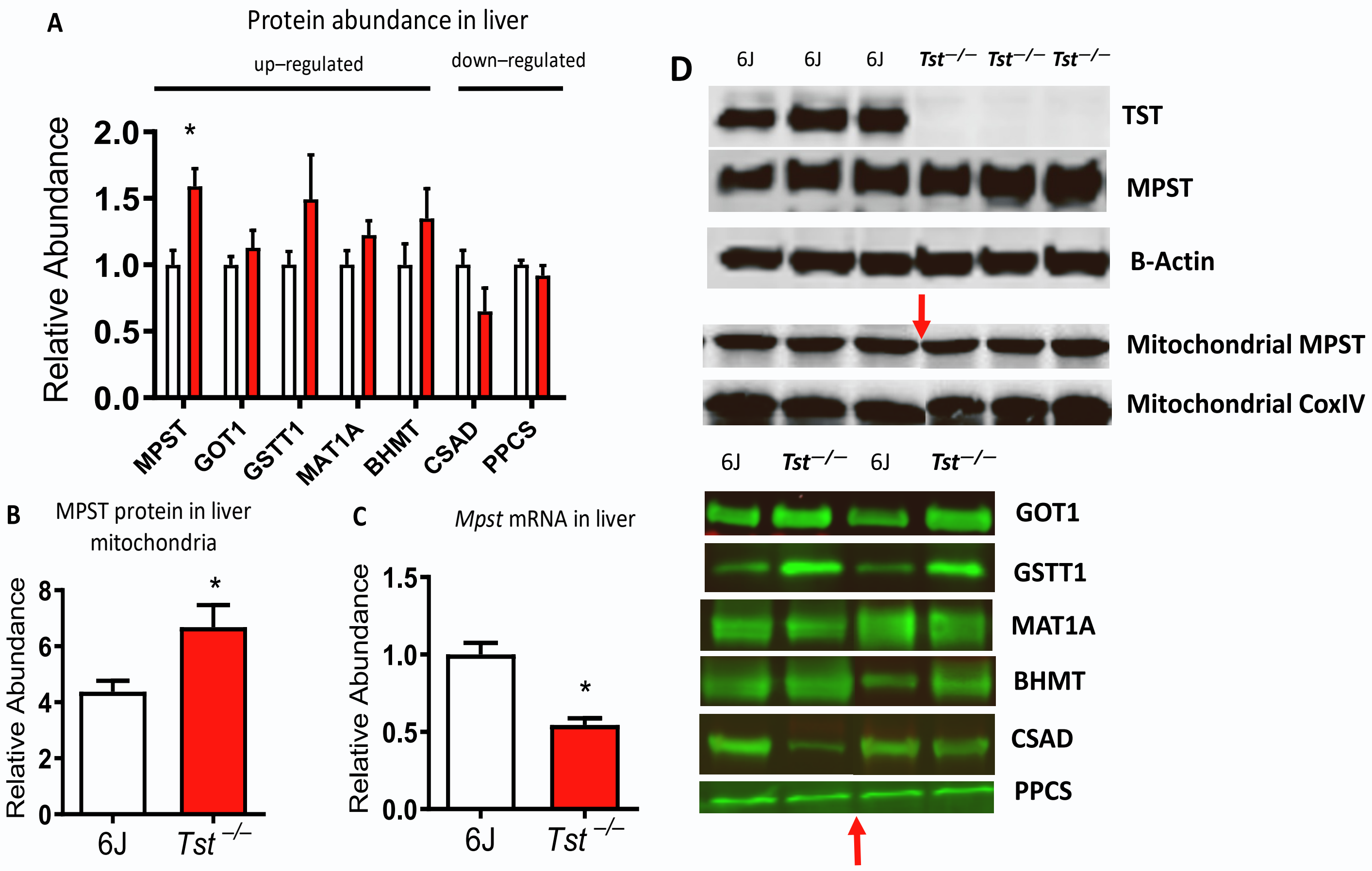

Figure S5. Validation of proteomic profiles by select western blot is exemplified by increased mitochondrial MPST. Related to Figure 2 and Table 3. (**A**) Quantification of western blots for a range of proteins found significantly up or down-regulated in the liver proteome of normal diet-fed 4 hour fasted C57Bl/6J (6J; white bar, n = 4-6) and *Tst*$^{-/-}$ (red bar, n = 4-6) mice. (**B**) Quantification of western blots for MPST from isolated liver mitochondria of normal diet-fed 4 hour fasted C57Bl/6J (white bar, n = 6), and *Tst*$^{-/-}$ (red bar, n = 6) mice. (**C**) *Mpst* mRNA quantified by real time PCR from liver of normal diet-fed C57Bl/6J (6J; white bar, n = 6) and *Tst*$^{-/-}$ (red bar, n = 6) mice. (**D**) Representative blots from LICOR imaging for the data quantified in (**A**). Red arrow indicates where superfluous lanes have been removed to simplify visualisation of genotype comparisons (GOT1, MAT1A, BHMT CSAD, mitochondrial MPST and COXIV). Data are represented as mean ±SEM. Significance was calculated using un-paired two-tailed student's t-test. * $P < 0.05$, ** $P < 0.01$, *** $P < 0.001$.

Figure S6. Hepatic proteins enriched in *Tst* $^{-/-}$ mice show under-representation of NRF2 promoter binding sites. Related to Figure 3 and Table 3.

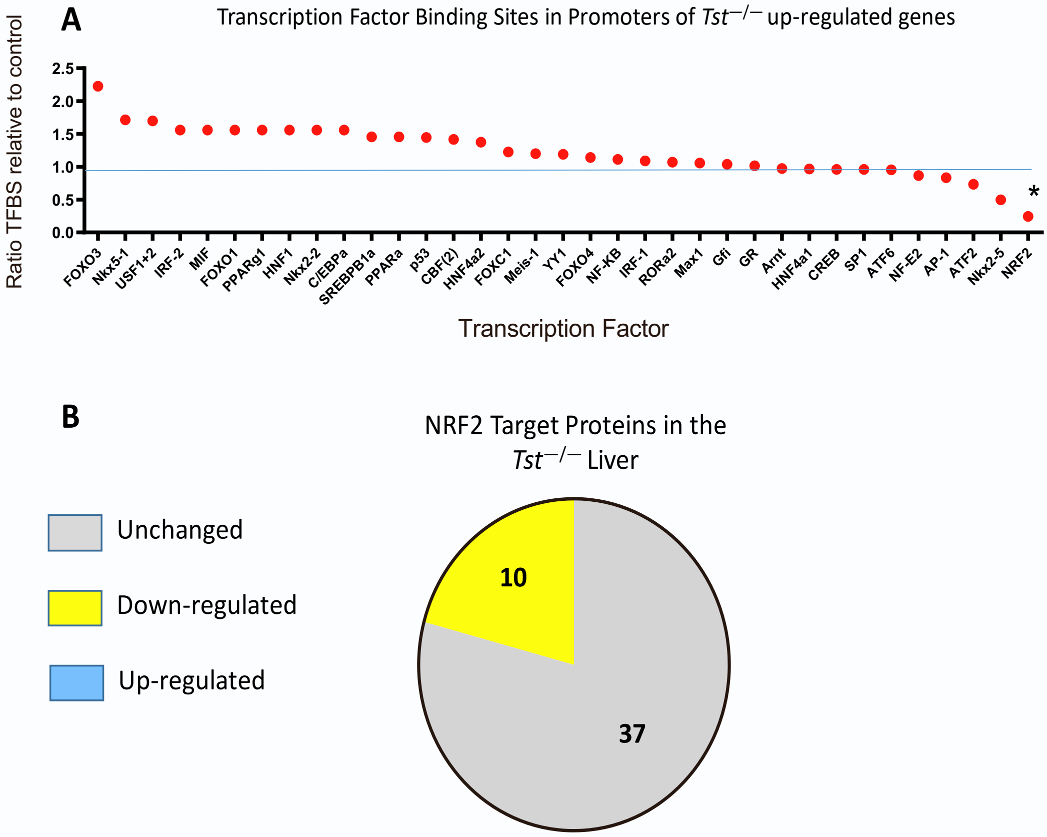

Figure S6. Hepatic proteins enriched in *Tst* $^{-/-}$ mice show under-representation of NRF2 promoter binding sites. Related to Figure 3 and Table 3. (**A**) abundance in *Tst*$^{-/-}$ liver compared to a control set of proteins that are unchanged between 6J and *Tst*$^{-/-}$. The proportion of genes containing a promoter binding site from proteins increased in *Tst*$^{-/-}$ was divided by the proportion of genes containing a binding site from a control set of genes. (**B**) Pie charts representing the number of NRF2-target proteins whose abundance is increased (blue), decreased (yellow) or unchanged (grey) in the Tst$^{-/-}$ liver. Significance of transcription factor enrichment analysis was calculated using a Fishers Exact test. * $P < 0.05$. Significance for NRF2 target abundance was performed with the Freeman-Halton Fishers Exact Test.

Figure S7. Hepatocyte respiration after high–fat feeding or after amino acid or pyruvate challenge is comparable between C57Bl/6J and *Tst*$^{-/-}$ mice *in vitro*. Related to Figure 4.

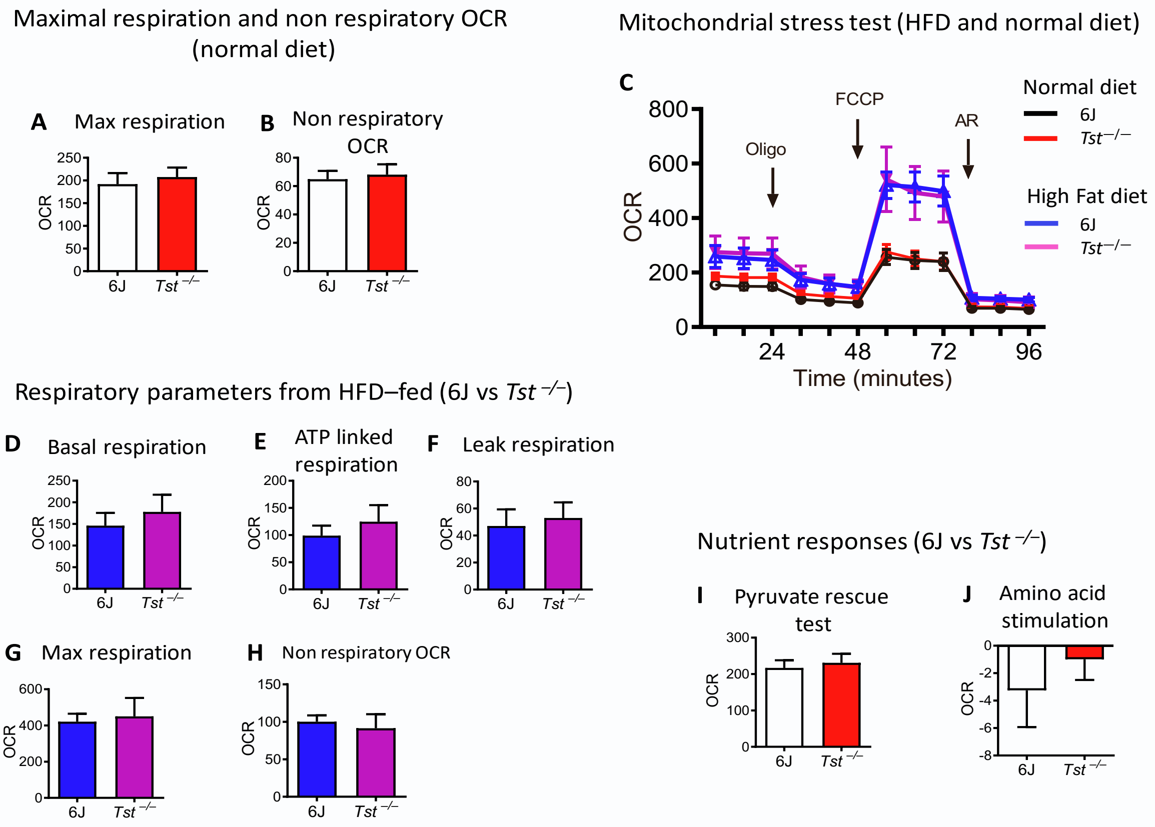

Figure S7. Hepatocyte respiration after high–fat feeding or after amino acid or pyruvate challenge is comparable between C57Bl/6J and *Tst*$^{-/-}$ mice *in vitro*. Related to Figure 4. (**A**) Maximal respiratory OCR elicited by uncoupling with FCCP, by hepatocytes from normal diet-fed C57Bl/6J (n = 6) or *Tst*$^{-/-}$ (n = 6) mice, calculated from Figure 3B. (**B**) Non-respiratory OCR remaining following the inhibition of respiration with antimycin and rotenone, by hepatocytes from normal diet-fed C57Bl/6J (n = 6) or *Tst*$^{-/-}$ (n = 6) mice, calculated from Figure 3B. (**C**) Seahorse trace representing the mean oxygen consumption rate (OCR), normalised to protein, by hepatocytes from normal diet-fed (n = 6/genotype), and high fat diet-fed (n = 4/genotype) C57Bl/6J and *Tst*$^{-/-}$mice during a mitochondrial stress test. (**D**)Basal respiratory OCR linked to ATP production (antimycin/rotenone sensitive) by hepatocytes from high fat diet-fed C57Bl/6J (n = 4) or *Tst*$^{-/-}$ (n = 4) mice, calculated from Figure S4C. (**E**) Respiratory OCR linked to ATP production (oligomycin sensitive) by hepatocytes from high fat diet-fed C57Bl/6J (n = 4) or *Tst*$^{-/-}$ (n = 4) mice, calculated from Figure S4C. (**F**) Respiratory OCR relating to proton leak (oligomycin insensitive) by hepatocytes from high fat diet-fed C57Bl/6J (n = 4) or *Tst*$^{-/-}$ (n = 4) mice, calculated from Figure S4C. (**G**) Maximal respiratory OCR elicited by uncoupling with FCCP, by hepatocytes from high fat diet-fed C57Bl/6J (n = 4) or *Tst*$^{-/-}$ (n = 4) mice, calculated from Figure S4C. (**H**) Non-respiratory OCR remaining following the inhibition of respiration with antimycin and rotenone, by hepatocytes high fat diet-fed C57Bl/6J (n = 4) or *Tst*$^{-/-}$ (n = 4) mice, calculated from Figure S4C. (**I**) Stimulation of maximal uncoupled respiration following addition of pyruvate (2mM), from normal diet-fed C57Bl/6J (n = 4) or *Tst*$^{-/-}$ (n = 4) mice. (**J**) Stimulation of maximal uncoupled respiration following addition of aspartate (1mM) and glutamax (1mM), by hepatocytes from normal diet-fed C57Bl/6J (n = 1) or *Tst*$^{-/-}$ (n = 1) mice. Data are represented as mean ±SEM. Significance was calculated using an unpaired two tailed, student's t-test. * P < 0.05.

Table S1. Parameters during the euglycemic hyperinsulinemic clamp

(A) Parameters during the basal (pre clamp) experiment (60-90 minutes post tracer)

Parameter	6J chow	*Tst*$^{-/-}$ chow	6J HFD	*Tst*$^{-/-}$ HFD	Genotype	Diet
Fasted Glucose 60 min (mg/dl)	116.35± 14.93	135.72± 7.22	146.50± 3.89	167.13± 9.68	*	**
Glycolysis (mg/kg/min)	11.63 ± 1.60	11.12 ± 0.62	12.89 ± 0.57	12.61 ± 0.62	ns	ns (0.09)
Glycogen synthesis (mg/kg/min)	21.48 ± 2.06	19.02 ± 2.04	15.08 ± 2.76	16.28 ± 2.50	ns	****

(B) Measurements and parameters during the clamp experiment (160-210 minutes post tracer)

Parameter	6J chow	*Tst*$^{-/-}$ chow	Genotype (chow)	6J HFD	*Tst*$^{-/-}$ HFD	Genotype (HFD)
Glucose 160 min (mg/dl)	108.0 ± 5.0	121.0 ± 10.1	ns	120.1± 3.7	125.6 ± 10.3	ns
Glucose 170 min (mg/dl)	120.3 ± 6.4	129.8 ± 5.8	ns	118.1 ± 4.1	143.9 ± 14.0	ns (0.08)
Glucose 180 min (mg/dl)	115.0 ± 5.5	125.5 ± 2.5	ns (0.08)	125.9 ± 2.9	126.7 ± 6.8	ns
Glucose 190 min (mg/dl)	124.0 ± 1.0	131.0 ± 4.6	ns	121.5 ± 4.7	116.1 ± 3.7	ns
Glucose 200 min (mg/dl)	121.0 ± 5.0	125.3 ± 5.8	ns	121.9 ± 3.0	115.7 ± 5.3	ns
Glucose 210 min (mg/dl)	113.0 ± 7.8	123.5 ± 4.6	ns	119.4 ± 4.0	116.9 ± 2.7	ns
Glucose IR 160-210 min (mg/kg/min)	84.9 ± 4.2	85.0 ± 2.6	ns	70.26 ± 4.83	69.95 ± 4.96	ns
Glucose IR 160 (mg/kg/min)	82.3 ± 5.1	87.1 ± 3.2	ns	68.8 ± 4.2	68.9 ± 5.1	ns
Glucose IR 170 (mg/kg/min)	85.2 ± 4.9	89.0 ± 3.0	ns	70.0 ± 5.1	74.6 ± 4.4	ns
Glucose IR 180 (mg/kg/min)	84.7 ± 4.4	83.5 ± 4.2	ns	70.6 ± 5.1	70.7 ± 6.2	ns
Glucose IR 190 (mg/kg/min)	85.2 ± 4.0	86.1 ± 2.1	ns	69.9 ± 4.8	69.1 ± 5.7	ns
Glucose IR 200 (mg/kg/min)	85.2 ± 4.0	84.8 ± 2.4	ns	70.4 ± 4.7	71.2 ± 4.5	ns
Glucose IR 210 (mg/kg/min)	85.2 ± 4.0	84.5 ± 2.4	ns	70.4 ± 4.7	71.2 ± 4.5	ns
Turnover (mg/kg/min)	85.97 ± 3.52	94.50 ± 3.87	ns	73.61 ± 5.07	63.08 ± 7.10	ns
Hepatic Glucose Prod. (mg/kg/min)	1.10 ± 5.31	9.92 ± 9.15	ns	3.326 ± 4.03	-6.83 ± 7.93	ns
Glycolysis (mg/kg/min)	45.90 ± 2.218	48.37 ± 2.05	ns	42.85 ± 1.48	36.42 ± 4.62	ns (0.19)
Glycogen synthesis (mg/kg/min)	40.06 ± 4.44	46.12 ± 2.68	ns	30.76 ± 5.36	26.66 ± 4.49	ns
Integral Glucose (dpm.min/mg)	$3.6e^{7} \pm 1.4e^{6}$	$2.95e^{7} \pm 1.2e^{6}$	*	$1.88e^{7} \pm 1.4e^{6}$	$1.7e^{7} \pm 1.6e^{6}$	ns
IWAT glucose utilization (ng/mg.min)	14.21 ± 4.05	18.62 ± 2.04	ns	4.53 ± 1.07	6.64 ± 1.00	ns
EWAT glucose utilization (ng/mg.min)	7.523 ± 4.39	7.21 ± 2.18	ns	2.94 ± 0.56	3.64 ± 0.39	ns
VL glucose utilization (ng/mg.min)	33.77 ± 2.98	38.66 ± 1.70	ns (0.17)	49.65 ± 9.19	47.42 ± 9.19	ns
EDL glucose utilization (ng/mg.min)	35.79 ± 11.09	40.86 ± 10.25	ns	68.79 ± 8.65	61.29 ± 11.80	ns
Soleus glucose utilization (ng/mg.min)	99.85 ± 12.38	123.60 ± 13.90	ns (0.13)	220.8 ± 24.45	198.5 ± 32.92	ns
Tibialis glucose utilization (ng/mg.min)	47.25 ± 7.22	53.67 ± 4.40	ns	74.39 ± 6.46	80.16 ± 6.09	ns
Heart glucose utilization (ng/mg.min)	161.40 ± 7.65	198.00 ± 14.09	ns (0.13)	226.6 ± 51.01	262.5 ± 23.29	ns
Liver glucose utilization (ng/mg.min)	3.53 ± 0.56	3.57 ± 0.63	ns	3.378 ± 0.39	3.58 ± 0.46	ns
End Clamp Insulin (µU/ml)	133.6 ± 7.03	126.4 ± 5.06	ns	147.2 ± 9.4	124.4 ± 10.9	ns

*** P < 0.05, ** P < 0.01, *** P < 0.001, **** P < 0.0001**

Table S1. Parameters during the euglycemic hyperinsulinemic clamp. Related to Figure 1. Metabolic parameters measured during continuous trace infusion but prior to clamp (**A**) and during maintenance of euglycemia and hyperinsulinemia (**B**) from C57Bl/6J (chow-fed, n = 3, hfd-fed, n = 8) and *Tst*$^{-/-}$ (chow-fed, n = 6, hfd-fed, n = 7) mice. Data are represented as mean ±SEM. Significance for the basal experiment (**A**) was

calculated using a 2-WAY ANOVA for *genotype* and *diet*. Significance for the clamp (**B**) was calculated for each diet separately using T-tests for *genotype*. * P < 0.05, ** P < 0.01, *** P < 0.001, **** P < 0.0001

**Table S2. Hydrogen sulfide disposal by hepatocytes and mitochondria (Amperometry)**			
nmoles/min/mg protein	**C57Bl/6J**	***Tst*$^{-/-}$**	**Significance**
Hepatocytes	3.88 +/- 0.095	4.15 +/- 0.345	**ns**
Hepatocytes (Respiratory)	1.97 +/- 0.176	2.91 +/- 0.288	*
Hepatocytes (Non-respiratory)	1.91 +/- 0.181	1.24 +/- 0.117	*
Liver Mitochondria	0.65 +/- 0.095	1.23 +/- 0.129	*
Liver Mitochondria (Respiratory)	0.26 +/- 0.060	0.50 +/- 0.080	*

*** P < 0.05**

Table S2. *Tst* deletion results in increased respiratory H_2S disposal by hepatocytes. Related to Figure 2 and Table 1. H_2S disposal rates (measured by gas selective amperometry following addition of 10 µM Na_2S) of hepatocytes (n = 6/genotype), or isolated liver mitochondria (n = 7/genotype) of ND-fed C57Bl/6J and *Tst*$^{-/-}$mice. Rates of H_2S disposal were measured with and without respiratory inhibition following addition of Antimycin (2µM). Antimycin insensitive disposal rates are referred to as non-respiratory. The Antimycin sensitive disposal rates are referred to as respiratory. Data are represented as mean ±SEM. Significance was calculated using paired two-tailed student's t-test. * P < 0.05.

Table S4. Sulfide metabolism proteins in liver proteome (*Tst*$^{-/-}$ vs C57Bl/6J, ND-fed)

Feature ID	Name		Fold Change	Significance
Q3UW66	MPST	Mercaptopyruvate sulfurtrasferase	1.27	**
Q8R086	SUOX	Sulfite Oxidase	1.06	Ns
Q3UDS4	SQOR	Sulfide quinone reductase-like	1.06	Ns
Q91WT9	CBS	Cystathionine beta-synthase	1.03	Ns
Q9DCM0	ETHE1	Ethylmalonic encephalopathy 1	-1.01	Ns
Q8VCNS	CTH	Cystathionine gamma-lyase	-1.02	Ns

*** Raw P < 0.05, ** Adjusted P < 0.05**

Table S4. *Tst* deletion selectively regulates MPST in the sulfide pathway of normal diet–fed mice. Related to Figure 3 and Table 3. Relative peptide abundance of proteins of the sulfide production and disposal pathway from the liver proteome of normal diet (ND) fed mice. 'Fold Change' indicates the relative abundance of the protein in *Tst*$^{-/-}$ relative to C57Bl/6J.

**Table S5. GO terms - Nutrient metabolism; reduced in ND *Tst*$^{-/-}$ liver (*Tst*$^{-/-}$ vs C57Bl/6J liver, ND-fed)**			
**GO-ID**	**Name**	**Genes**	**Significance**
0006629	Lipid metabolic process	19	**
0006631	Fatty acid beta-oxidation	7	**
0003995	Acyl-CoA dehydrogenase activity	3	*
0047617	Acyl-CoA hydrolase activity	2	*

*** P < 0.05, ** P < 0.01**

Table S5. *Tst* Deletion results in reduction of selective fatty acid specific GO terms. Related to Figure 3 and Table 3. Significant GO terms (glucose or lipid related) represented by proteins that are less abundant in the ND-fed *Tst*$^{-/-}$ liver compared with ND-fed C57Bl/6J. 'Genes' indicates the number of genes in the *Tst*$^{-/-}$ that represent the changes driving the GO term.

Table S6 Insulin regulated proteins in *Tst*$^{-/-}$ and C57Bl/6J mice

(A) Abundance of peptides of insulin–induced proteins (*Tst*$^{-/-}$ vs C57Bl/6J, ND-fed)

Feature ID	Name	Fold change	Significance
Q3UGT1	CPT1A	1.03	Ns
P19096	FASN	-1.04	Ns
Q3UDA8	CPT2	-1.05	Ns
Q3V2G1	APOA1	-1.10	Ns
Q5SVI5	GCK	-1.14	*

(B) Abundance of peptides of insulin–suppressed proteins (*Tst*$^{-/-}$ vs C57Bl/6J, ND-fed)

Feature ID	Name	Fold change	Significance
QO5421	CYP2E1	1.13	**
Q9D6M3	SLC25AA2	-1.03	Ns
Q8CI37	PCK1	-1.09	Ns
Q3UJ70	HMGCS1	-1.09	Ns
O08601	MTTP	-1.20	**

(C) Abundance of peptides of insulin–induced proteins (*Tst*$^{-/-}$ vs C57Bl/6J, High Fat-fed)

Feature ID	Name	Fold change	Significance
Q3UGT1	CPT1A	1.03	Ns
P19096	FASN	-1.04	Ns
Q3UDA8	CPT2	-1.05	Ns
Q3V2G1	APOA1	-1.1	Ns
Q5SVI5	GCK	-1.14	*

(D) Abundance of peptides of insulin–suppressed proteins (*Tst*$^{-/-}$ vs C57Bl/6J, High Fat-fed)

Feature ID	Name	Fold change	Significance
QO5421	CYP2E1	-1.05	Ns
Q9D6M3	SLC25AA2	-1.01	Ns
Q8CI37	PCK1	1.09	Ns
Q3UJ70	HMGCS1	-1.04	Ns
O08601	MTTP	1.09	*

*** Raw P < 0.05, ** Adjusted P < 0.05**

Table S6. Proteins regulated by insulin are broadly comparable in expression between *Tst*$^{-/-}$ and C57Bl/6J. Related to Figure 3. Relative abundance in proteins that are known to be induced (A) or suppressed

(B) by insulin in the liver, from the liver proteome of normal diet fed mice. 'Fold Change' indicates the relative abundance of the protein in *Tst*$^{-/-}$ relative to C57Bl/6J. Relative abundance in proteins that are known to be induced (C) or suppressed (D) by insulin in the liver, from the liver proteome of high fat diet fed mice. 'Fold Change' indicates the relative abundance of the protein in *Tst*$^{-/-}$ relative to C57Bl/6J.

Table S7. KEGG pathways shared by high fat feeding and TST deletion

Entry	Name	Comparison	Significance
**A. Shared up-regulated pathways**			
00260	Glycine, serine and threonine metabolism	*Tst*$^{-/-}$ vs 6J	**
		HFD vs ND	**
**B. Shared down-regulated pathways**			
00980	Metabolism of xenobiotics by cytochrome P450	*Tst*$^{-/-}$ vs 6J	****
		HFD vs ND	*
00982	Drug metabolism – cytochrome P450	*Tst*$^{-/-}$ vs 6J	****
		HFD vs ND	*
04142	Lysosome	*Tst*$^{-/-}$ vs 6J	**
		HFD vs ND	****
04390	Hippo signaling pathway	*Tst*$^{-/-}$ vs 6J	**
		HFD vs ND	****
05215	Prostate cancer	*Tst*$^{-/-}$ vs 6J	**
		HFD vs ND	*
04024	cAMP signaling pathway	*Tst*$^{-/-}$ vs 6J	*
		HFD vs 6J	*
04141	Protein processing endoplasmic reticulum	*Tst*$^{-/-}$ vs 6J	*
		HFD vs 6J	**
05211	Renal cell carcinoma	*Tst*$^{-/-}$ vs 6J	*
		HFD vs 6J	*
04722	Neurotrophin signaling pathway	*Tst*$^{-/-}$ vs 6J	*
		HFD vs 6J	*
04110	Cell cycle	*Tst*$^{-/-}$ vs 6J	*
		HFD vs 6J	**
04918	Thyroid hormone synthesis	*Tst*$^{-/-}$ vs 6J	*
		HFD vs 6J	***
04612	Antigen processing and presentation	*Tst*$^{-/-}$ vs 6J	*
		HFD vs 6J	**

*** P < 0.05, ** P < 0.01, *** P < 0.001 **** P < 0.0001**

Table S7. KEGG Pathways shared by high fat feeding and TST deletion. Related to Figure 3. (**A**) KEGG pathways that are significantly up-regulated in the same direction by both high fat diet (HFD vs ND), and *Tst* deletion (*Tst*$^{-/-}$ vs C57Bl/6J). (**B**) KEGG pathways that are significantly down-regulated in the same direction by both high fat diet (HFD vs ND), and *Tst* deletion (*Tst*$^{-/-}$ vs C57Bl/6J). 'Comparison' indicates the two groups being compared.

Table S8. Effect of high fat feeding on sulfide pathway proteins (High fat diet vs ND-fed)					
**Feature ID**	**Name**	**Fold change in 6J**	**Significance**	**Fold change in *Tst*$^{-/-}$**	**Significance**
Q3UW66	MPST	1.35	**	1.15	*
Q8R086	SUOX	1.21	**	1.23	**
Q545S0	TST	1.19	Ns	n/a	n/a
Q8VCNS	CTH	-1.04	Ns	-1.06	Ns
Q9DCM0	ETHE1	-1.05	Ns	1.03	Ns
Q3UDS4	SQOR	-1.08	Ns	1.01	Ns
Q91WT9	CBS	-1.10	*	-1.06	Ns

*** Raw P < 0.05, ** Adjusted P < 0.05**

Table S8. Effect of high fat feeding on the sulfide pathway of C57Bl/6J and *Tst*$^{-/-}$ mice. Related to Figure 3. Protein abundances of the sulfide production and disposal pathway from the liver proteome of C57Bl/6J and *Tst*$^{-/-}$ mice. 'Fold Change' indicates the relative abundance of the protein in high fat diet fed mice relative to normal diet fed mice, shown separately for each genotype.

Table S9. Pathways in *Tst*$^{-/-}$ that are regulated oppositely to high fat feeding

Entry	Name	Comparison	Direction
**A**	**KEGG Pathways**		
00980	Metabolism of xenobiotics by cytochrome P450	*Tst*$^{-/-}$ vs 6J	Decreased
		HFD vs ND	Increased
00983	Drug metabolism – other enzymes	*Tst*$^{-/-}$ vs 6J	Decreased
		HFD vs ND	Increased
00053	Ascorbate and aldarate metabolism	*Tst*$^{-/-}$ vs 6J	Decreased
		HFD vs ND	Increased
00040	Pentose and glucoronate interconversions	*Tst*$^{-/-}$ vs 6J	Decreased
		HFD vs ND	Increased
00830	Retinol metabolism	*Tst*$^{-/-}$ vs 6J	Decreased
		HFD vs ND	Increased
**B**	**GO Terms**		
0006629	Lipid metabolic process	*Tst*$^{-/-}$ vs 6J	Decreased
		HFD vs ND	Increased
0006631	Fatty acid beta-oxidation	*Tst*$^{-/-}$ vs 6J	Decreased
		HFD vs ND	Increased
0003995	Acyl-CoA dehydrogenase activity	*Tst*$^{-/-}$ vs 6J	Decreased
		HFD vs ND	Increased
0047617	Acyl-CoA hydrolase activity	*Tst*$^{-/-}$ vs 6J	Decreased
		HFD vs ND	Increased

Table S9. KEGG pathways and GO terms that are regulated in the opposite direction by high fat feeding compared to Tst deletion. Related to Figure 3. (**A**) KEGG pathways that are regulated in the opposite direction by high fat diet (HFD-fed C57Bl/6J vs ND-fed C57Bl/6J), to *Tst* deletion (ND-fed *Tst*$^{-/-}$ vs C57Bl/6J). (**B**) GO terms that are regulated in the opposite direction by high fat diet (HFD-fed C57Bl/6J vs ND-fed C57Bl/6J), to *Tst* deletion (ND-fed *Tst*$^{-/-}$ vs C57Bl/6J). 'Comparison' Indicates the two groups being compared. 'Direction' indicates whether the protein abundance is decreased or increased in the first group relative to the second.
